# Synthesis of silylbut-1-en-3-ynes and buta-1,3-dienes as building blocks *via* hydrosilylation of 1,4-bis(trimethylsilyl)buta-1,3-diyne

**DOI:** 10.1038/s41598-024-82198-w

**Published:** 2024-12-16

**Authors:** Kinga Stefanowska-Kątna, Jędrzej Walkowiak, Adrian Franczyk

**Affiliations:** Center for Advanced Technologies, Adam Mickiewicz University, Uniwersytetu Poznańskiego 10, Poznań, 61-614 Poland

**Keywords:** 1,4-Bis(trimethylsilyl)buta-1,3-diyne, Hydrosilylation, But-1-en-3-ynes, 1,3-dienes, Building blocks, Catalysis, Inorganic chemistry, Organic chemistry, Chemical synthesis

## Abstract

The selective and efficient synthesis of (*E*)-1,2,4-trisilylbut-1-en-3-ynes obtained *via* Pt-catalyzed hydrosilylation of 1,4-bis(trimethylsilyl)buta-1,3-diyne is described. Optimized reaction conditions (Pt(PPh_3_)_4_) or Pt_2_(dvs)_3_, 100 °C, toluene, 18 h) yielded compounds with high isolation yields (76–95%). The modification of (*E*)-1,2,4-trisilylbut-1-en-3-ynes was further tested in protodesilylation, halodesilylation, hydrosilylation, and Pd-based cross-coupling reactions, resulting in a broad spectrum of new products. These compounds represent new families of buta-1,3-dienes, (*E*,*E*)-2,3-bissilylbuta-1,3-dienes, (*E*,*E*)-1,2,3-trisilylbuta-1,3-dienes, and (*E*,*E*)-1,2,3-trisilylbuta-1,3-dienes, which are ideally suited to silicon-based transformations and are perfect models for the investigation of the reactivity of one silyl group in the presence of another.

**Supplementary Information:**

The online version contains supplementary material available at 10.1038/s41598-024-82198-w.

## Introduction

The 1,3-diynes represent an important and highly reactive group of compounds providing *fine chemicals* and advanced materials by various chemical transformations. One of the simplest and most readily available representatives of this group is 1,4-bis(trimethylsilyl)buta-1,3-diyne (**1**). It can be synthesized by the silylation of butadiyne^1^ or the oxidative coupling of trimethylsilylacetylene^2,3^. **1** is a commercially available, thermally (mp = 107–109 ℃) and air-stable crystalline compound which is used in the synthesis of a broad spectrum of molecules with conjugated unsaturated carbon-carbon bonds due to the easy replacement of the silyl groups with different functions. For example, 1,4-bis(trimethylsilyl)buta-1,3-diyne has been applied in the synthesis of (*E*)-2-methyl-1,4-bis(trimethylsilyl)-1-buten-3-ynes *via* a carbometallation process^4^, glycosylated oligo(ethylene)s using the Negishi reaction^5^, and (±) falcarinol, a member of the polyacetylene class of fatty alcohols^6^. In general, functional reagents with conjugated double (C = C) and triple (C ≡ C) carbon-carbon bonds can offer key motifs found in numerous natural products or drugs with a wide spectrum of biological activity^7–9^.

**Fig. 1:**
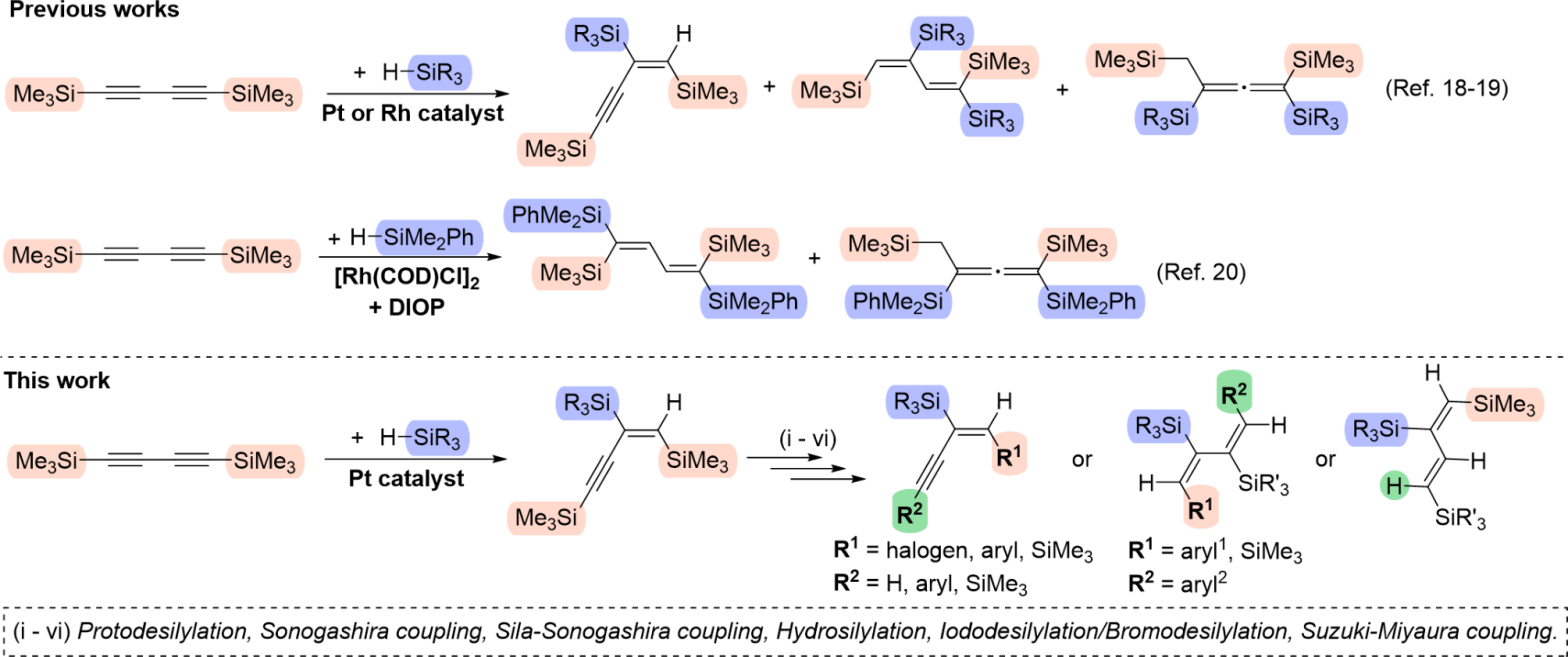
Catalytic hydrosilylation of 1,4-bis(trimethylsilyl)buta-1,3-diyne.

The hydrosilylation of 1,3-diynes is the most documented of all the hydroelementation processes of these compounds^10–12^. The formation of silylated 1-en-3-ynes, 1,3-dienes, allenes, polymers, or cyclic compounds can vary based on the catalyst, reagents, their concentrations and ratios, and the process conditions, often resulting in a complex mixture of products. The detailed optimization of these factors is crucial if the desired product is to be obtained selectively. The scientific literature describes numerous examples of hydrosilylation processes involving 1,3-diynes, which have also been developed in our group^10,11,13–17^. However, only three studies have described the hydrosilylation of 1,4-bis(trimethylsilyl)buta-1,3-diyne (Fig. 1)^18–20^. Hiyama’s group reported the hydrosilylation of 1,4-bis(trimethylsilyl)buta-1,3-diyne with platinum and rhodium catalysts, which proceeded stepwise to give first 2-silyl-1,4-bis(trimethylsilyl)but-1-en-3-ynes and then 1,3-disilyl-1,4-bis(trimethylsilyl)buta-1,2-dienes in high yield^18,19^. The allene was produced using H_2_PtCl_6_ and HSiEt_3_ (3–4 eq., 80 ℃, 0.5 h), while for the bulkier silane HSi(*i*-Pr)_3_, 2-silyl-1,4-bis(trimethylsilyl)but-1-en-3-yne was formed. In other cases, a mixture of allenes and enynes was obtained. Surprisingly, the use of HSiMe_2_Cl led to the formation of bishydrosilylated product where the silyl groups were attached to C(1) and C(3) atoms, 1,3-bis(chlorodimethylsilyl)-1,4-bis(trimethylsilyl)buta-1,2-diene. On the other hand, the use of Pt(PPh_3_)_4_ catalyst led to the formation of monohydrosilylated product. The rhodium complex RhCl(PPh_3_)_3_ gave a mixture of but-1-en-3-yne and an allene. However, after a longer reaction time, the allene became the exclusive product. The authors discussed the fact that the first addition of silane leads to the *cis*-but-1-en-3-yne with hydrogen attached to C(1) and silicon to C(2). The second addition of silane leads to buta-1,3-dienes *via* another 1,2-addition to the C ≡ C bond of but-1-en-3-yne but with a different regioselectivity, or to an allene *via* 1,4-addition. Tillack et al. described the Rh and Ni-catalyzed hydrosilylation of 1,4-bis(trimethylsilyl)buta-1,3-diyne to chiral allenes in the presence of chiral phosphine ligands^20^. Two bissilylation products were found in the reaction of **1** with HSiMe_2_Ph in the presence of [Rh(COD)Cl]_2_ and DIOP (DIOP - (−)-2,3-*O*-*iso*propylidene-2,3-dihydroxy-1,4-bis(diphenylphosphino)butane): (*E*,* E*)-1,4-bis(dimethylphenylsilyl)-1,4-bis(trimethylsilyl)buta-1,3-diene and an allene. The authors disproved the thesis of the two-step mechanism of the hydrosilylation process. They observed only *cis-*addition of silanes in the reaction. Whether the silyl groups are added at the inner or outer C-atoms depends on both the substrates and the catalyst.

Herein, we would like to use the recent achievement in the field of 1,3-diynes hydrosilylation and take it one step further by modifying the silyl groups in the resulting building blocks. These studies show the possibility of applying these reagents in the synthesis of compounds with conjugated C = C or C = C and C ≡ C bonds. The obtained products are perfect models for investigating the reactivity of silyl groups, including one in the presence of another, finally resulting in 3-en-1-yl and 1,3-butadienyl derivatives with different surrounding C = C bonds.

## Results and discussion

Based on our experience^13–15,17,21–26^ and the literature screening^10,12,18–20,27−36^, we selected six commercially available catalysts (Pt(PPh_3_)_4_, Pt_2_(dvs)_3_, PtO_2_, H_2_PtCl_6_, Pt(en)Cl_2_, [Ir(cod)Cl]_2_) to test their potential in the selective mono- and bishydrosilylation of C ≡ C triple bonds of 1,4-bis(trimethylsilyl)buta-1,3-diyne (**1**) (Table S1-S5). A wide spectrum of silanes (**2a**-**j**) were tested. For each silane, the catalysts and reaction conditions were selected separately.

**Table 1:** Synthesis of 1,2,4-trisilylbut-1-en-3-ynes (**3a**-**j**) by the hydrosilylation of **1** with silanes **2a**-**j**.


Entry	Silane SiR_3_**(2)**	Catalyst	[1]: [2]: [Pt]	Conv. of 2 [%]	Selectivity of 3/4/other [%]
1	HSiMe_2_Bn (**2a**)	Pt(PPh_3_)_4_	1:1:0.01	> 99	100/0/0
2	HSiEt_3_ (**2b**)	Pt(PPh_3_)_4_	1:1:0.01	> 99	100/0/0
3	HSi(*i*-Pr)_3_ (**2c**)	Pt(PPh_3_)_4_	1:1:0.01	88	85/3/0
4	HSi(*n*-Oct)_3_ (**2d**)	Pt(PPh_3_)_4_	1:1:0.01	> 99	100/0/0
5	HSi(OEt)_3_ (**2e**)	Pt(PPh_3_)_4_	1:1:0.01	94	88/0/6
6	HSiMe(OSiMe_3_)_2_ (**2f**)	Pt(PPh_3_)_4_	1:1:0.01	> 99	100/0/0
7	HSi(OSiMe_3_)_3_ (**2 g**)	Pt(PPh_3_)_4_	1:1:0.01	97	95/2/0
8	H_2_SiPh_2_ (**2 h**)	Pt_2_(dvs)_3_	1:1:0.0001	83	81/2/0
9	HSiPh_3_ (**2i**)	Pt(PPh_3_)_4_	1:1:0.01	> 99	100/0/0
10	HSiMe_2_Cl (**2j**)	Pt_2_(dvs)_3_	1:2:0.0008	> 99	100/0/0

Reaction conditions: m_**1**_ = 0.1 g, 4 mL of toluene, 18 h, argon. The conversion of reagents was determined by^1^H NMR spectroscopy and GC-MS. The selectivity was determined by^1^H, ^13^C, and ^29^Si NMR spectroscopy. Dvs − 1,3-divinyl-1,1,3,3-tetramethyldisiloxane.

For the synthesis of products **3a-g** and **3i** the highest yields were obtained when Pt(PPh_3_)_4_ was applied as the catalyst (Table 1, entries 1–7, 9). The products **3a-b**,** 3d**,** 3f** and **3i** were selectively synthesized, while the selectivity of **3c**, **3e** and **3g** reached 85%, 88% and 95%, respectively. Additionally, lower yields for the hydrosilylation of **1** with triisopropylsilane (**2c**), triethoxysilane (**2e**) and tris(trimethylsiloxy)silane (**2g**) (88%, 94% and 97%, respectively) were noticed compared to the reactions with **2a-b**,** 2d**,** 2f** and **2i** (> 99%). The Pt(PPh_3_)_4_ catalyst was ineffective in synthesizing products **3 h** and **3j**, leading to a mixture of products (Table S4, entry 2; Table S5, entry 7).

**Fig. 2:**
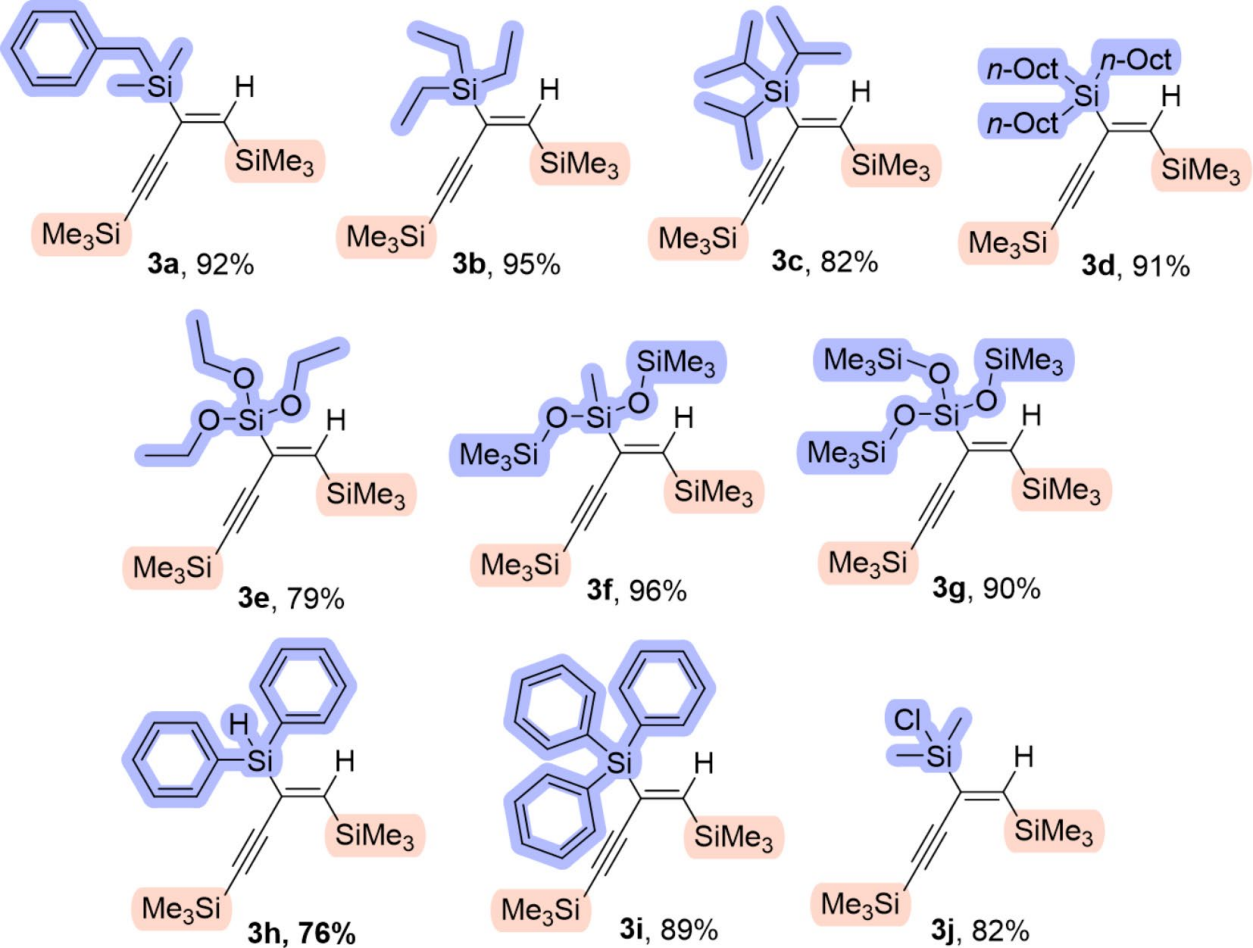
Structural formulas of obtained compounds with the isolated yields indicated.

On the other hand, Karstedt’s catalyst proved to be a better choice for these reactions (Table 1, entries 8, 10). Product **3j** was obtained selectively, while product **3h** was obtained with 81% selectivity. The optimized reaction conditions for the hydrosilylation of 1,4-bis(trimethylsilyl)buta-1,3-diyne (**1**) with silanes **2a-j** involved a temperature of 100 °C, toluene as a solvent, and a duration of 18 h. Stoichiometric amounts of reagents were used for the synthesis of compounds **3a-i**, while a two-fold excess was applied when silane **2j** was used. The structures of the obtained products (**3a-j**) with indicated isolated yields (76–95%) are presented in Fig. 2. Compounds **3a**, and **3d-j** were synthesized and characterized for the first time. The products incorporate different silyl groups in their structures, carefully selected to enable typical Si-based transformations.

The application of hexachloroplatinic acid (H_2_PtCl_6_), used in previously reported studies for the synthesis of bisadducts or allenes^18–20^, in the hydrosilylation of **1** with HSiEt_3_ (**2b**) and HSiMe_2_Cl (**2j**) resulted in a complex mixture of products, from which it was impossible to isolate specific compounds (Table S2, entries 5–7; Table S5, entries 8–9). Our attempts to obtain products of bishydrosilylation using the methods we developed for aryl- and alkyl substituted 1,3-diynes were also unsuccessful under all tested conditions^14,17^. In all cases, a mixture of products was formed.

**Fig. 3:**
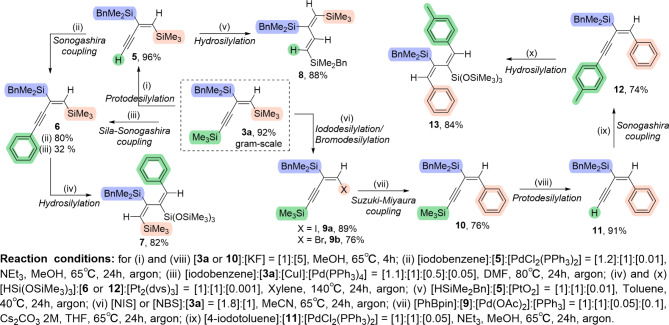
Synthesis of compounds **5**–**13** by the application of **3a** as a building block.

The presence of the C = C and C ≡ C bonds and three silyl moieties makes products **3a-j** extremely attractive building blocks in organic synthesis. To verify this, the catalytic transformations of **3a** were demonstrated (Fig. 3).

Due to differences in the reactivity of the silyl groups in **3a**, the investigation of the reactivity of one silyl group in the presence of another was possible. The C_sp_-SiMe_3_ group can be selectively utilized in the sila-Sonogashira reaction or protodesilylation followed by the Sonogashira coupling sequence, giving unreported product **6** with isolated yields 32–80%, respectively. Compound **6** is the first example of a 1-en-3-yne with two silyl groups around the C = C bond and an aryl group connected to C ≡ C bond. In the direct hydrosilylation of non-symmetric Ar-C ≡ C-C ≡ C-SiMe_3_, the addition of silane is favored by the C ≡ C bond with the aryl group.

Subsequently, the obtained product **6** was transformed *via* a hydrosilylation reaction with HSi(OSiMe_3_)_3_ (**2g**) into product **7**, a buta-1,3-diene with four different substituents at each C = C double bond.

It is noteworthy that the silyl group (Si(OSiMe₃)₃) was added to the C(3) carbon, consistent with our observations in the hydrosilylation of buta-1,3-diynes with alkyl and/or aryl substituents^17^. Compound **7**, possessing three different silyl groups in its structure, can be considered a multifunctional building block. Reduction of the C ≡ C bond by the Si-H group introduces two reactive functionalities into the molecular structure and enables the design of the geometry surrounding the second C = C bond. Notably, compound **7** is the first example of a buta-1,3-diene with two different silyl groups attached to the internal carbon atoms, while the aryl and SiMe_3_ groups are bonded to the external carbon atoms. Compound **7** cannot be obtained through direct hydrosilylation of non-symmetric Ar-C ≡ C-C ≡ C-SiMe_3_, as the second addition of silane to the 1-en-3-yne was unsuccessful in all previously reported protocols.

The reactivity of product **5**, obtained through a selective protodesilylation of **3a**, was examined in a hydrosilylation reaction. The process involved the *syn*-addition of HSiMe_2_Bn (**2a**) to the C ≡ C bond, resulting in product **8** with an isolated yield of 88%. The obtained product represents a second example of the family of buta-1,3-dienes possessing three different silyl groups: (*E*,*E*)-1,2,3-trisilylbuta-1,3-dienes.

The C_sp2_-SiMe_3_ group in **3a** was modified through an iododesilylation or bromodesilylation process to produce product **9a** or **9b** with a high isolated yield (89% and 76%, respectively). This enabled the application of the Suzuki-Miyaura coupling, leading to the formation of product **10**. Compound **10** is an analog of **7**, where the silyl group is attached to another C ≡ C bond. This demonstrates an alternative modification route for the selective functionalization of both C = C bonds obtained from the reduction of the C ≡ C bonds originally present in the structure of buta-1,3-diyne **1**.

1-En-3-yne **12** was obtained through Sonogashira coupling of compound **11** with 4-iodotoluene. Compound **12** contains two different internal aryl groups in its structure. It is important to note that the selective synthesis of such a compound cannot be achieved through direct hydrosilylation of non-symmetric 1,3-diynes, as the addition occurs equally at both C ≡ C bonds. This compound represents the first reported example of a 1-aryl_1_-4-aryl_2_-2-silyl-1-en-3-yne.

Product **12**, through a hydrosilylation reaction, led to the formation of buta-1,3-diene **13** with two different aryl groups attached to the external carbon atoms and two different silyl groups bonded to the internal carbon atoms. Such a compound has not been obtained by any other method so far. This example of the transformation route confirms that compounds **3a-j** are extremely useful building blocks in organic synthesis. In the structure of the final product **13** from compound **3a**, only the SiMe₂Bn group and the proton remain unchanged.

Attempts to synthesize buta-1,3-diene by reacting **3a** with HSiMe_2_Bn (**2a**) under the same reaction conditions used for the synthesis of compounds **7** and **13** (Pt_2_(dvs)_3_, xylene, 140 °C, argon) but with either a longer reaction time (up to 96 h) or a 40-fold increase in the amount of catalyst resulted in a mixture of products. At best, the bishydrosilylated product was obtained with only 35% selectivity. However, due to the complexity of the mixture, it was not possible to isolate the product. This proves that the selective synthesis of 1,3-dienes with an SiMe_3_ group in the structure using buta-1,3-diyne **1** as a substrate is only possible through at least a three-step reaction, where, in the second step, one SiMe_3_ group is replaced with another substituent, such as a proton or phenyl.

It is worth highlighting that products **5**–**13** are new and previously unreported compounds. They represent reactive building blocks that can be modified through a series of different reactions, making them suitable for applications in the synthesis of many natural products or drugs with a broad spectrum of biological activity^7–9^.

## Conclusions

In summary, we successfully obtained a series of (*E*)-1,2,4-trisilylbut-1-en-3-ynes (**3a-j**) by *cis*-hydrosilylation of 1,4-bis(trimethylsilyl)buta-1,3-diyne (**1**) with silanes (**2a-j**). Based on the detailed optimization of reaction conditions, the platinum catalysts (Pt(PPh_3_)_3_ and Pt_2_(dvs)_3_) were chosen as the most selective and effective. The processes were carried out using a stoichiometric ratio of reagents, except for the synthesis of **3j**, where a 2-fold excess of silane was applied, at 100 ℃, in toluene for 18 h. The resulting products **3a-j** were obtained with isolated yields ranging from 76 to 95%. Compounds **3a** and **3d**-**j** were synthesized and characterized for the first time. Surprisingly, the synthesis of buta-1,3-dienes *via* the hydrosilylation of **1** with silane **2b** or **2j** as well as the hydrosilylation of **3a** with **2a**, catalyzed by hexachloroplatinic acid (H_2_PtCl_6_) and carried out under conditions described in the literature^18–20^, led to a complex mixture of products.

The obtained products **3a-j** are known as attractive building blocks for the synthesis of more complex molecules. Their reactivity was demonstrated using the example of the modification of **3a**, which was obtained on a gram scale. Through a series of catalytic transformations, including protodesilylation, halodesilylation, hydrosilylation, and Pd-based cross-coupling reactions, ten new compounds (**5**–**13**) with high isolated yields (74–96%) were obtained. Compounds **7**, **8** and **13** are examples of new buta-1,3-diene families with two or three different silyl groups in their structures: (*E*,*E*)-2,3-bissilylbuta-1,3-dienes (**13**), (*E*,*E*)-1,2,3-trisilylbuta-1,3-dienes (**7**), and (*E*,*E*)-1,2,3-trisilylbuta-1,3-dienes (**8**). Products **7** and **13** possess different substituents around each of two C = C bonds.

In this study, we have demonstrated that through hydrosilylation and other well-described catalytic processes from the literature, using commercially available reagents and catalysts, it is possible to synthesize a wide spectrum of functional but-1-en-3-ynes and buta-1,3-dienes, whose structures can be freely decorated with various functional groups. The obtained products are highly suited to silicon-based transformations and are perfect models for further investigation of the reactivity of one silyl group in the presence of another.

## Methods

The reactions were carried out in an argon atmosphere using Schlenk line techniques^37^.

### General procedure for the synthesis of products 3a-j

The 1,4-bis(trimethylsilyl)buta-1,3-diyne (0.1 g, 0.514 mmol, **1**) and appropriate solid catalyst (PtO_2_ or Pt(PPh_3_)_4_ or Pt(en)Cl_2_ (en = 1,2-diaminoethane) or [Ir(cod)Cl]_2_ (cod = 1,5-cyclooctadiene) were added to a Schlenk flask with a Rotaflo^®^ stopcock equipped with a magnetic stirrer and dried under vacuum (25 °C, 10^− 3^ mbar) for 15–20 min. Then, the flask was quickly flushed with argon, and anhydrous and degassed solvent (toluene (4 mL), xylene (4 mL) or MeCN (1 mL)) was added. Subsequently, an appropriate silane **2a-j** (0.514 mmol – 5.14 mmol) was added, and the reaction mixture was heated to 40–140 °C or kept at room temperature and stirred for 0.5–72 h. For the reactions in the presence of Pt_2_(dvs)_3_ (dvs = 1,1,3,3-tetramethyl-1,3-divinyldisiloxane), the catalyst was added to the reaction mixture in the last step before it was heated. Afterwards, the crude reaction mixture was analyzed by GC-MS and ^1^H NMR and purified by flash chromatography, according to the procedure described in subsection 2.6 in ESI. The remaining details regarding the synthesis of appropriate products are included in Tables S1-S5.

### Procedure for the gram-scale synthesis of product 3a

The 1,4-bis(trimethylsilyl)buta-1,3-diyne (1 g, 5.14 mmol, **1**) and Pt(PPh_3_)_4_ (0.064 g, 0.05 mmol) were added to a Schlenk flask with a Rotaflo^®^ stopcock equipped with a magnetic stirrer and dried under vacuum (25 ℃, 10^− 3^ mbar) for 15–20 min. Then, the flask was quickly flushed with argon, and anhydrous and degassed toluene (20 mL) was added. Next, benzyldimethylsilane (**2a**) (815 µl, 5.14 mmol) was added and the reaction mixture was heated to 100 ℃ and stirred for 18 h. Afterwards, the crude reaction mixture was analyzed by GC-MS and ^1^H NMR and purified by flash chromatography, according to the procedure described in subsection 2.6 in ESI.

## Electronic supplementary material

Below is the link to the electronic supplementary material.

Supplementary Material 1

## Data Availability

All data generated or analyzed during this study are included in this published article and its supplementary information file.
