# Supplementary material for: Synthesis of silylbut-1-en-3-ynes and buta-1,3-dienes as building blocks via hydrosilylation of 1,4-bis(trimethylsilyl)buta-1,3-diyne
Source: Sci Rep. 2024 Dec 16;14:30499. doi: 10.1038/s41598-024-82198-w (PMC11649804; doi:10.1038/s41598-024-82198-w)
Supplement: Supplementary file 1 — Supplementary Material 1 [file 41598_2024_82198_MOESM1_ESM.pdf]

Supporting Information

Synthesis of silylbut-1-en-3-ynes and buta-1,3-dienes as building blocks *via* hydrosilylation of 1,4-bis(trimethylsilyl)buta-1,3-diyne

Kinga Stefanowska-Kątna[a], Jędrzej Walkowiak[a] and Adrian Franczyk[a,*]

[a]Center for Advanced Technologies, Adam Mickiewicz University, Uniwersytetu Poznańskiego 10, Poznań 61-614, Poland

E-mail: adrian.franczyk@amu.edu.pl

Table of Contents

1. Synthesis of products **3a-b**, **3g-h**, **3j** – optimization of the reaction conditions 2
2. General information 7
   2.1 Materials 7
   2.2 NMR analyses 7
   2.3 GC-MS analysis 7
   2.4 Elemental analyses 7
   2.5 FT-IR analysis 7
   2.6 Products purification 7
3. General procedures for product **3a** functionalization 8
   3.1 Protodesilylation (for products **5** and **11**) 8
   3.2 Sonogashira coupling (for products **6** and **12**) 8
   3.3 Sila-Sonogashira coupling (for product **6**) 8
   3.4 Hydrosilylation (for products **7** and **13**) 8
   3.5 Hydrosilylation (for product **8**) 9
   3.6 Iododesilylation (for product **9**) 9
   3.7 Suzuki-Miyaura coupling (for product **10**) 9
4. Products characterization 10

1. Synthesis of products 3a-b, 3g-h, 3j– optimization of the reaction conditions

Table S1. Synthesis of 3a by hydrosilylation of 1,4-bis(trimethylsilyl)buta-1,3-diyne (1) with benzyldimethylsilane (2a).

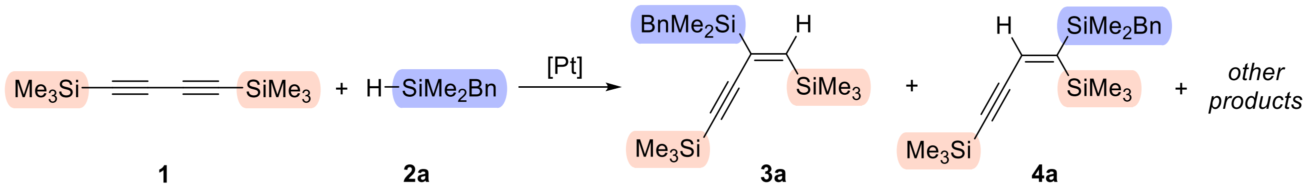

Entry	[1]:[2a]:[Pt]	Catalyst	Solvent	Time [h]	Temperature [°C]	Conversion of Si-H [%]	Selectivity of 3a/4a/other products [%]
**1.**	**1:1:0.01**	**$Pt(PPh_3)_4$**	**Toluene**	**24**	**100**	**>99**	**100/0/0**
2.	1:2:0.0008	$Pt_2(dvs)_3$	Toluene	24	100	>99	72/0/28
3.	1:2:0.0008	$Pt_2(dvs)_3$	Xylene	24	140	>99	56/0/44
4.	1:4:0.008	$Pt_2(dvs)_3$	Xylene	24	140	>99	31/0/69
5.	1:2:0.01	$Pt(NH_2CH_2Cl)_2$	Xylene	72	140	>99	16/0/36
6.	1:1:0.02	$[Ir(cod)Cl]_2$	MeCN[a]	24	rt	75	59/0/16

Reaction conditions: $m_1/V_{Tol./Xylene}$ = 0.025 g/ml, argon; [a]m_1/V_{MeCN} = 0.1 g/ml, argon; dvs - 1,3-divinyl-1,1,3,3-tetramethyldisiloxane; Conversions of reagents were determined by ^1H NMR spectroscopy and GC MS. The selectivity was determined by ^1H, ^{13}C and ^{29}Si NMR spectroscopy. The best results are bolded and included in the manuscript.

Table S2. Synthesis of 3b by hydrosilylation of 1,4-bis(trimethylsilyl)buta-1,3-diyne (1) with triethylsilane (2b).

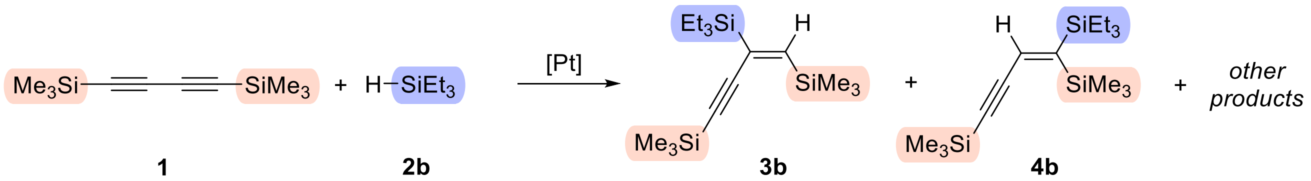

Entry	[1]:[2b]:[Pt]	Catalyst	Solvent	Time [h]	Temperature [°C]	Conversion of Si-H [%]	Selectivity of 3b/4b/other products [%]
**1.**	**1:1:0.01**	**$Pt(PPh_3)_4$**	**Toluene**	**24**	**100**	**>99**	**100/0/0**
2.	1:1:0.0008	$Pt_2(dvs)_3$	Toluene	24	rt	93	76/0/17
3.	1:2:0.0008	$Pt_2(dvs)_3$	Toluene	24	100	>99	48/4/48
4.	1:2:0.0008	$Pt_2(dvs)_3$	Xylene	24	140	>99	0/0/100
5.	1:4:0.002	H_2PtCl_6/*i*-PrOH	-	0,5	80	>99	15/1/84
6.	1:4:0.002	H_2PtCl_6/*i*-PrOH	-	0,5	100	>99	29/0/71
7.	1:4:0.002	H_2PtCl_6/*i*-PrOH	-	0,5	80	>99	19/0/81
8.	1:1:0.02	$[Ir(cod)Cl]_2$	MeCN[a]	24	rt	75	complex mixture

Reaction conditions: $m_1/V_{Tol./Xylene}$ = 0.025 g/ml, argon; [a]m_1/V_{MeCN} = 0.1 g/ml, argon; dvs - 1,3-divinyl-1,1,3,3-tetramethyldisiloxane; Conversions of reagents were determined by ^1H NMR spectroscopy and GC MS. The selectivity was determined by ^1H, ^{13}C and ^{29}Si NMR spectroscopy. The best results are bolded and included in the manuscript.

Table S3. Synthesis of 3g by hydrosilylation of 1,4-bis(trimethylsilyl)buta-1,3-diyne (1) with tris(trimethylsiloxy)silane (2g).

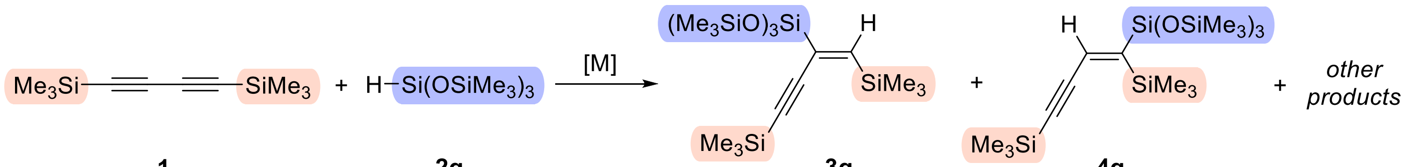

1 2g 3g 4g

Entry	[1]:[2g]:[M]	Catalyst	Solvent	Time [h]	Temperature [°C]	Conversion of Si-H [%]	Selectivity of 3g/4g/other products [%]
**1.**	**1:1:0.01**	**$Pt(PPh_3)_4$**	**Toluene**	**24**	**100**	**97**	**95/2/0/0**
2.	1:1:0.02	$[Ir(cod)Cl]_2$	MeCN[a]	24	rt	6	6/0/0/0
3.	1:1:0.0008	$Pt_2(dvs)_3$	Toluene	24	rt	8	8/0/0/0
4.	1:1:0.0008	$Pt_2(dvs)_3$	Toluene	24	100	69	25/0/0/44
5.	1:2:0.01	$Pt(NH_2CH_2Cl)_2$	Xylene	72	140	>99	26/0/37/37

Reaction conditions: $m_1/V_{Tol./Xylene}$ = 0.025 g/ml; [a]m_1/V_{MeCN} = 0.1 g/ml, argon; dvs - 1,3-divinyl-1,1,3,3-tetramethyldisiloxane; cod- cyclooctadiene; Conversions of reagents were determined by ^1H NMR spectroscopy and GC MS. The selectivity was determined by ^1H, ^{13}C and ^{29}Si NMR spectroscopy. The best results are bolded and included in the manuscript.

Table S4. Synthesis of 3h by hydrosilylation of 1,4-bis(trimethylsilyl)buta-1,3-diyne (1) with diphenylsilane (2h).

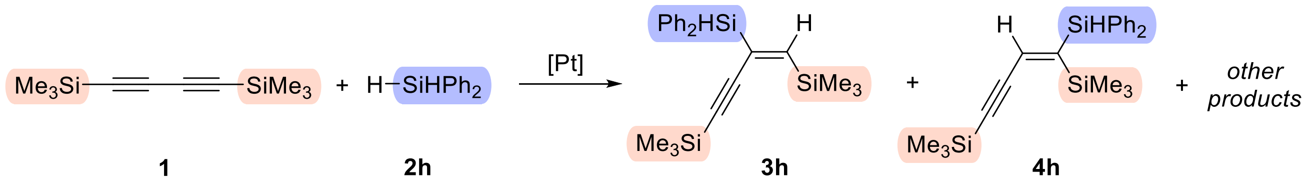

Entry	[1]:[2h]:[Pt]	Catalyst	Solvent	Time [h]	Temperature [°C]	Conversion of Si-H [%]	Selectivity of 3h/4h/other products [%]
**1.**	**1:1:0.0001**	**$Pt_2(dvs)_3$**	**Toluene**	**24**	**100**	**>99**	**94/0/0/6**
2.	1:1:0.01	$Pt(PPh_3)_4$	Toluene	24	100	75	12/0/0/63
3.	1:1:0.02	$[Ir(cod)Cl]_2$	MeCN[a]	24	rt	0	-

Reaction conditions: $m_1/V_{Tol.}$ = 0.025 g/ml, argon; [a]m_1/V_{MeCN} = 0.1 g/ml; dvs - 1,3-divinyl-1,1,3,3-tetramethyldisiloxane; Conversions of reagents were determined by ^{1}H NMR spectroscopy and GC MS. The selectivity was determined by ^{1}H, ^{13}C and ^{29}Si NMR spectroscopy. The best results are bolded and included in the manuscript.

Table S5. Synthesis of 3j by hydrosilylation of 1,4-bis(trimethylsilyl)buta-1,3-diyne (1) with chlorodimethylsilane (2j).

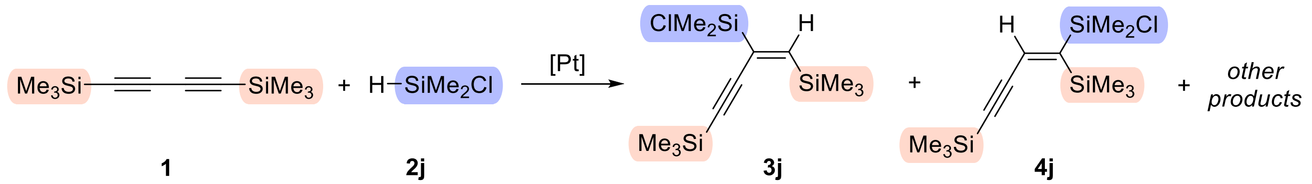

Entry	[1]:[2j]:[Pt]	Catalyst	Solvent	Time [h]	Temperature [°C]	Conversion of Si-H [%]	Selectivity of 3j/4j/other products [%]
**1.**	**1:2:0.0008**	**$Pt_2(dvs)_3$**	**Toluene**	**24**	**100**	>99	100/0/0
2.	1:1:0.0008	$Pt_2(dvs)_3$	Toluene	24	100	32	32/0/0
3.	1:2:0.0008	$Pt_2(dvs)_3$	Xylene	24	140	89	72/17/0
4.	1:4:0.0008	$Pt_2(dvs)_3$	Xylene	24	140	>99	58/6/36
5.	1:8:0.0008	$Pt_2(dvs)_3$	Xylene	24	140	>99	Complex mixture
6.	1:1:0.2	PtO_2	Toluene	24	40	0	-
7.	1:1:0.01	$Pt(PPh_3)_4$	Toluene	24	100	>99	Complex mixture
8.	1:2:0.002	H_2PtCl_6/*i*-PrOH	-	24	80	>99	72/0/28
9.	1:10:0.002	H_2PtCl_6/*i*-PrOH	-	24	100	>99	Complex mixture
10.	1:2:0.02	$[Ir(cod)Cl]_2$	MeCN[a]	24	rt	91	91/0/0

Reaction conditions: $m_1/V_{Tol./Xylene} = 0.025$ g/ml, argon; [a]$m_1/V_{MeCN} = 0.1$ g/ml; dvs - 1,3-divinyl-1,1,3,3-tetramethyldisiloxane; Conversions of reagents were determined by ^{1}H NMR spectroscopy and GC MS. The selectivity was determined by ^{1}H, ^{13}C and ^{29}Si NMR spectroscopy. The best results are bolded and included in the manuscript.

2. General information

2.1 Materials

1,4-Bis(trimethylsilyl)buta-1,3-diyne (98%, Sigma-Aldrich), Benzyldimethylsilane (98%, Fluorochem), Triethylsilane (97%, Sigma-Aldrich), Triethoxysilane (95%, Sigma-Aldrich), Tris(trimethylsiloxy)silane (≥98%, Sigma-Aldrich), Diphenylsilane (97%, Sigma-Aldrich), Chlorodimethylsilane (98%, Sigma-Aldrich), Triisopropylsilane (98%, Sigma-Aldrich), Trioctylsilane (95%, Sigma-Aldrich), 1,1,1,3,5,5,5-Heptamethyltrisiloxane (97%, Sigma-Aldrich), Triphenylsilane (97%, Sigma-Aldrich), 4-iodotoluene (99%, Sigma-Aldrich), iodobenzene (98%, Sigma-Aldrich), N-Iodosuccinimide (95%, Sigma-Aldrich), N-Bromosuccinimide (≥99%, Sigma-Aldrich), Phenylboronic acid pinacol ester (97%, Sigma-Aldrich), Copper(I) iodine (≥98%, Sigma-Aldrich), Triethylamine (≥99%, Sigma-Aldrich), Triphenylphosphine (≥99%, Sigma-Aldrich), Bis(triphenylphosphine)palladium (II) dichloride (98%, Sigma-Aldrich), Tetrakis(triphenylphosphine)palladium (0) (99%, Sigma-Aldrich), Palladium(II) acetate (98%, Angene), Cesium carbonate (≥99%, Sigma-Aldrich), Platinum(0)-1,3-divinyl-1,1,3,3-tetramethyldisiloxane (Karstedt's catalyst, solution in xylene, Pt 2%, Sigma-Aldrich), Platinum(IV) oxide (surface area ≥ 75m^2/g, Sigma-Aldrich), Tetrakis(triphenylphosphine)platinum(0) (97%, Sigma-Aldrich), Dichloro(ethylenediamine)platinum(II) (99%, Sigma-Aldrich), Chlorobis(cyclooctene)iridium(I)dimer (97%, Sigma-Aldrich), Hexachloroplatinic(IV) acid hexahydrate (~40% Pt) (Sigma-Aldrich). Chloroform-d (99.96 atom% D, Sigma-Aldrich), Methanol (≥99.8%, anhydrous, Sigma-Aldrich), Acetonitrile (99.8%, anhydrous, Sigma-Aldrich), Toluene, N,N-Dimethylformamide were dried using standard procedures, deoxygenated and stored over molecular sieves 4 Å under argon atmosphere (toluene, N,N-dimethylformamide). Xylene, *n*-hexane, ethyl acetate, 2-propanol were purchased from Avantor Performance Materials Poland. Argon (99,999%) was purchased from Linde. Silica gel (MN-Kieselgel 60, 0.04-0.063 mm (230-400 mesh ASTM; Sigma-Aldrich)) was used as received.

2.2 NMR analyses

^{1}H, ^{13}C, and ^{29}Si NMR spectra were recorded at 25°C on a Bruker Ultra Shield 300 MHz and Bruker Ascend 400 MHz NANOBAY spectrometers. $CDCl_3$ was used as a solvent and for internal deuterium lock. Chemical shifts are reported in ppm with reference to the residual portion solvent peak for ^{1}H and ^{13}C NMR, to TMS for ^{29}Si NMR. The multiplicities were reported as follow: singlet (s), doublet (d), doublet of doublets (dd), triplet (t) and multiplet (m).

2.3 GC-MS analysis

GC-MS analyses were performed on a Bruker 450-GC with a 30 m Varian DB-5 0.25 mm capillary column and a Scion SQ-MS mass spectrometry detector. Two temperature programs were used a) 80°C (3 min), 10°C/min, 250°C (30 min), b) 150°C (3 min), 10°C/min, 280°C (44.5 min).

2.4 Elemental analyses

Elemental analyses were carried out on a Vario EL III analyzer. The content of hydrogen and carbon was obtained as data in percentage.

2.5 FT-IR analysis

FT-IR spectra were measured on a Nicolet iS50 FT-IR spectrometer (Thermo Scientific) equipped with a built-in ATR accessory with ATR diamond unit. In all experiments, 16 scans at a resolution of 2 cm^{-1} were used to record the spectra.

2.6 Products purification

The products were purified on silica by flash chromatography (Biotage IsoleraOne chromatograph) with UV detector (λ_1 = 255 nm, λ_2= 280 nm). Purification details: cartridge 10 g, flow rate: 8 mL/min, length: 10 CV (CV = column volume), phase: hexane/ethyl acetate (step 1: hexane 100% by 4 CV, step 2:

gradient 10%/CV by 4 CV, step 3: hexane 50% by 2 CV). Products were characterized by GC-MS or ^{1}H, ^{13}C, ^{29}Si NMR, FT-IR analyses.

3. General procedures for product 3a functionalization

3.1 Protodesilylation (for products 5 and 11)

(*E*)-(2-(Benzyldimethylsilyl)but-1-en-3-yne-1,4-diyl)bis(trimethylsilane) (0.3 g, 0.87 mmol, **3a**) or (*E*)-benzyldimethyl(1-phenyl-4-(trimethylsilyl)but-1-en-3-yn-2-yl)silane (0.205 g, 0.59 mmol, **10**) and potassium fluoride (0.253 g, 4.35 mmol, for **3a**), potassium fluoride (0.172 g, 2.95 mmol, for **10**), and 20 mL (for **3a**) or 14 mL (for **10**) of methanol were placed in a round bottom flask equipped with a stirring bar under an air atmosphere. Subsequently, the reaction mixture was stirred and heated to 65°C for 4h. After this time the solvent was removed under vacuum. The crude residue was dissolved in hexane and filtrated through a silica. The product was characterized by GC-MS, FT-IR, EA, ^{1}H, ^{13}C and ^{29}Si NMR analyses. Procedure according to the reference [1].

3.2 Sonogashira coupling (for product 6)

$PdCl_2(PPh_3)_2$ (0.006 g, 0.0084 mmol), (*E*)-benzyldimethyl(1-(trimethylsilyl)but-1-en-3-yn-2-yl)silane (0.227 g, 0.84 mmol, **5**) and iodobenzene (0.206 g, 1.00 mmol) were added to a Schlenk flask with a Rotaflo® stopcock equipped with a magnetic stirrer and dried under vacuum (25°C, 10^{-3} mbar) for 15–20 minutes. Then, the flask was flushed quickly with argon, and anhydrous and degassed MeOH (9 mL) and triethylamine (2.8 mL) were added. Subsequently, the reaction mixture was stirred and heated to 65°C for 24h. The crude reaction mixture was analyzed by GC-MS and ^{1}H NMR analyzes and purified according to the procedure in 2.6 subsection. The product was characterized by GC-MS, FT-IR, EA, ^{1}H, ^{13}C and ^{29}Si NMR analyses. Procedure according to the reference [1].

3.3 Sonogashira coupling (for product 12)

$PdCl_2(PPh_3)_2$ (0.009 g, 0.013 mmol), (*E*)-benzyldimethyl(1-phenylbut-1-en-3-yn-2-yl)silane (0.07 g, 0.25 mmol, **11**), and 4-iodotoluene (0.054 g, 0.25 mmol) were added to a Schlenk flask with a Rotaflo® stopcock equipped with a magnetic stirrer and dried under vacuum (25°C, 10^{-3} mbar) for 15–20 minutes. Then, the flask was flushed quickly with argon, and anhydrous and degassed MeOH (2 mL) and triethylamine (0.2 mL) were added. Subsequently, the reaction mixture was stirred and heated to 65°C for 24h. The crude reaction mixture was analyzed by GC-MS and ^{1}H NMR analyzes and purified according to the procedure in 2.6 subsection. The product was characterized by GC-MS, FT-IR, EA, ^{1}H, ^{13}C and ^{29}Si NMR analyses. Procedure according to the reference [1].

3.4 Sila-Sonogashira coupling (for product 6)

$Pd(PPh_3)_4$ (0.017 g, 0.0145 mmol), CuI (0.0282 g, 0.145 mmol), (*E*)-(2-(Benzyldimethylsilyl)but-1-en-3-yne-1,4-diyl)bis(trimethylsilane) (0.1 g, 0.29 mmol, **3a**) and iodobenzene (0.066 g, 0.32 mmol) were added to a Schlenk flask with a Rotaflo® stopcock equipped with a magnetic stirrer and dried under vacuum (25°C, 10^{-3} mbar) for 15–20 minutes. Then, the flask was flushed quickly with argon, and anhydrous and degassed DMF (3 mL) was added. Subsequently, the reaction mixture was stirred and heated to 80°C for 24h. The crude reaction mixture was analyzed by GC-MS and ^{1}H NMR analyzes. Procedure according to the reference [2].

3.5 Hydrosilylation (for products 7 and 13)

(*E*)-Benzyldimethyl(4-phenyl-1-(trimethylsilyl)but-1-en-3-yn-2-yl)silane (0.232 g, 0.67 mmol, **6**) or (*E*)-benzyl(4-(4-methoxyphenyl)-1-phenylbut-1-en-3-yn-2-yl)dimethylsilane (0.04 g, 0.11 mmol, **12**) and tris(trimethylsiloxy)silane (233.3 μl, 0.67 mmol for **7**), (38 μl, 0.11 mmol for **13**) were added to a Schlenk flask with a Rotaflo® stopcock equipped with a magnetic stirrer and dried under vacuum (25°C, 10^{-3} mbar) for 15–20 minutes. Then, the flask was flushed quickly with argon, and degassed xylene (3 mL

for **6**), (0.5 mL for **13**) and Karstedt's catalyst (7.5 µl for **6**), (1.25 µl for **13**) were added. Subsequently, the reaction mixture was stirred and heated to 140°C for 24h. The crude reaction mixture was analyzed by GC-MS and ^{1}H NMR analyzes and purified according to the procedure in 2.6 subsection. The product was characterized by GC-MS, FT-IR, EA, ^{1}H, ^{13}C and ^{29}Si NMR analyses. Procedure according to the reference [3].

3.6 Hydrosilylation (for product 8)

(*E*)-Benzyldimethyl(1-(trimethylsilyl)but-1-en-3-yn-2-yl)silane (0.100 g, 0.37 mmol, **5**) and PtO_2 (0.001g, 0.01 mol of Pt/mol of SiH) were added to a Schlenk flask with a Rotaflo® stopcock equipped with a magnetic stirrer and dried under vacuum (25°C, 10^{-3} mbar) for 15–20 minutes. Then, the flask was flushed quickly with argon, and anhydrous and degassed toluene (4 mL) and benzyldimethylsilane (59,8 µl, 0.37 mmol) were added. Subsequently, the reaction mixture was stirred and heated to 40°C for 24h. The crude reaction mixture was analyzed by GC-MS and ^{1}H NMR analyzes and purified according to the procedure in 2.6 subsection. The product was characterized by GC-MS, FT-IR, EA, ^{1}H, ^{13}C and ^{29}Si NMR analyses. Procedure according to the reference [3].

3.7 Iododesilylation/Bromodesilylation (for product 9a and 9b)

(*E*)-(2-(Benzyldimethylsilyl)but-1-en-3-yne-1,4-diyl)bis(trimethylsilane) (0.3 g, 0.87 mmol, **3a**) and *N*-iodosuccinimide (0.371 g, 1.57 mmol, for **9a**) or *N*-bromosuccinimide (0.279 g, 1.57 mmol, for **9b**) were added to a Schlenk flask with a Rotaflo® stopcock equipped with a magnetic stirrer and dried under vacuum (25°C, 10^{-3} mbar) for 15–20 minutes. Then, the flask was flushed quickly with argon, and anhydrous and degassed MeCN (18 mL) was added. Subsequently, the reaction mixture was stirred and heated to 65°C for 24h. The crude reaction mixture was analyzed by GC-MS and ^{1}H NMR analyzes and purified according to the procedure in 2.6 subsection. The product was characterized by GC-MS, FT-IR, EA, ^{1}H, ^{13}C and ^{29}Si NMR analyses. Procedure according to the reference [1].

3.8 Suzuki-Miyaura coupling (for product 10)

$[Pd(OAc)_2]$ (0.009 g, 0.0387 mmol), PPh_3 (0.02 g, 0.0774 mmol), (*E*)-benzyl(1-iodo-4-(trimethylsilyl)but-1-en-3-yn-2-yl)dimethylsilane (0.309 g, 0.774 mmol, **9**), phenylboronic acid pinacol ester (0.163 g, 0.774 mmol) were added to a Schlenk flask with a Rotaflo® stopcock equipped with a magnetic stirrer and dried under vacuum (25°C, 10^{-3} mbar) for 15–20 minutes. Then, the flask was flushed quickly with argon, and anhydrous and degassed THF (8 mL) and aqueous solution of Cs_2CO_3 (3M, 8 mL) were added. Subsequently, the reaction mixture was stirred and heated to 65°C for 24h. The crude reaction mixture was analyzed by GC-MS and ^{1}H NMR analyzes and purified according to the procedure in 2.6 subsection. The product was characterized by GC-MS, FT-IR, EA, ^{1}H, ^{13}C and ^{29}Si NMR analyses. Procedure according to the reference [4].

4. Products characterization

(*E*)-(2-(Benzyldimethylsilyl)but-1-en-3-yne-1,4-diyl)bis(trimethylsilane) (3a) – New compound

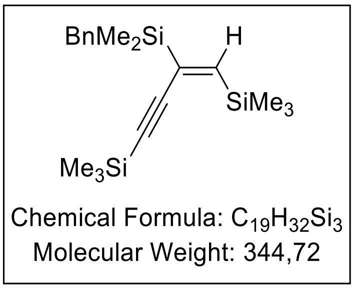

^{1}H NMR ($CDCl_3$, 300 MHz, δ, ppm): 7.26-7.00 (m, 5H, Ph), 6.48 (s, 1H, =CH), 2.24 (s, 2H, CH_2Ph), 0.22 (s, 9H, $SiCH_3$), 0.20 (s, 9H, $SiCH_3$), 0.12 (s, 6H, $SiCH_3$). **^{13}C NMR** ($CDCl_3$, 151 MHz, δ, ppm): 157.3 (=CH), 143.3 (C=CH), 139.8, 128.5, 128.2, 124.2 (Ph), 107.1 (≡$CSiMe_3$), 104.2 (C≡$CSiMe_3$), 25.0 (CH_2Ph), 0.1, -0.9 ($Si(CH_3)_3$), -4.2 ($Si(CH_3)_2$). **^{29}Si NMR** ($CDCl_3$, 191 MHz, δ, ppm): -2.41 ($SiMe_2$Bn), -8.58 ($SiMe_3$), -18.75 ($SiMe_3$). **GC-MS** (EI, 70 eV) m/z (rel. int., %): 344.2 (M^+, 0.6), 253.1 (36.4), 165.0 (13.3), 156.1 (16.2), 155.0 (92.5), 149.0 (13.2), 121.0 (24.3), 91.0 (12.5), 73.0 (100.0). **FT-IR** (cm^{-1}): 3025, 2956, 2898, 1601, 1493, 1452, 1406, 1246, 1206, 1154, 830, 795, 758, 696, 508. **Elem. Anal.** calcd for $C_{19}H_{32}Si_3$: C, 66.20; H, 9.36; found C, 66.28; H, 9.40. Pale yellow oil. Isolated yield = 92% (163.1 mg).

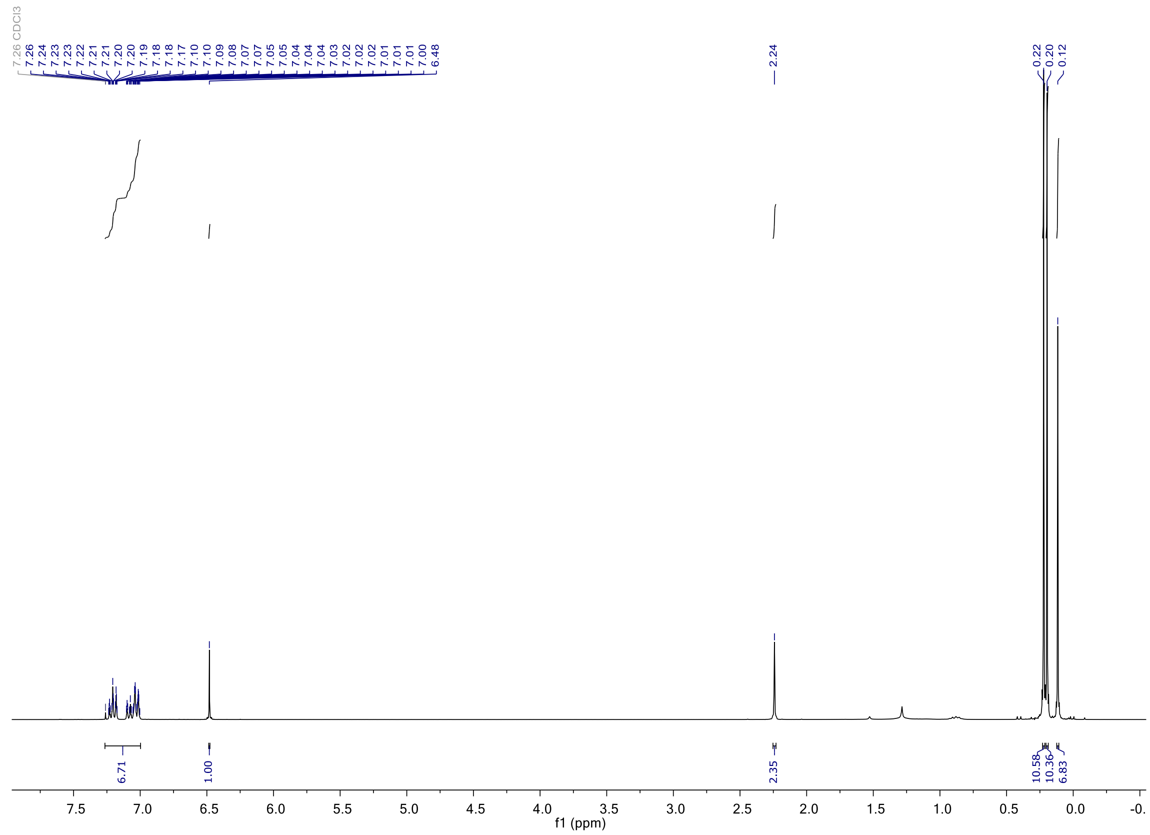

Figure S1. ^{1}H NMR spectrum of **3a**.

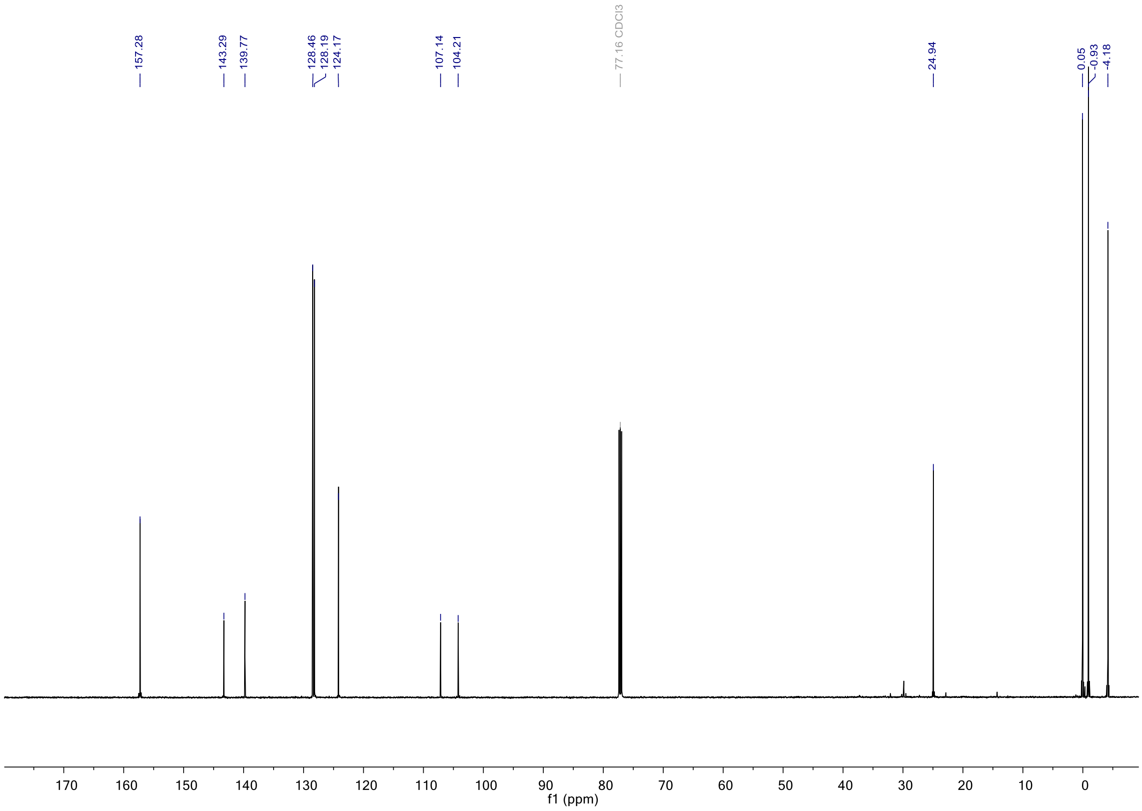

Figure S2. ^{13}C NMR spectrum of **3a**.

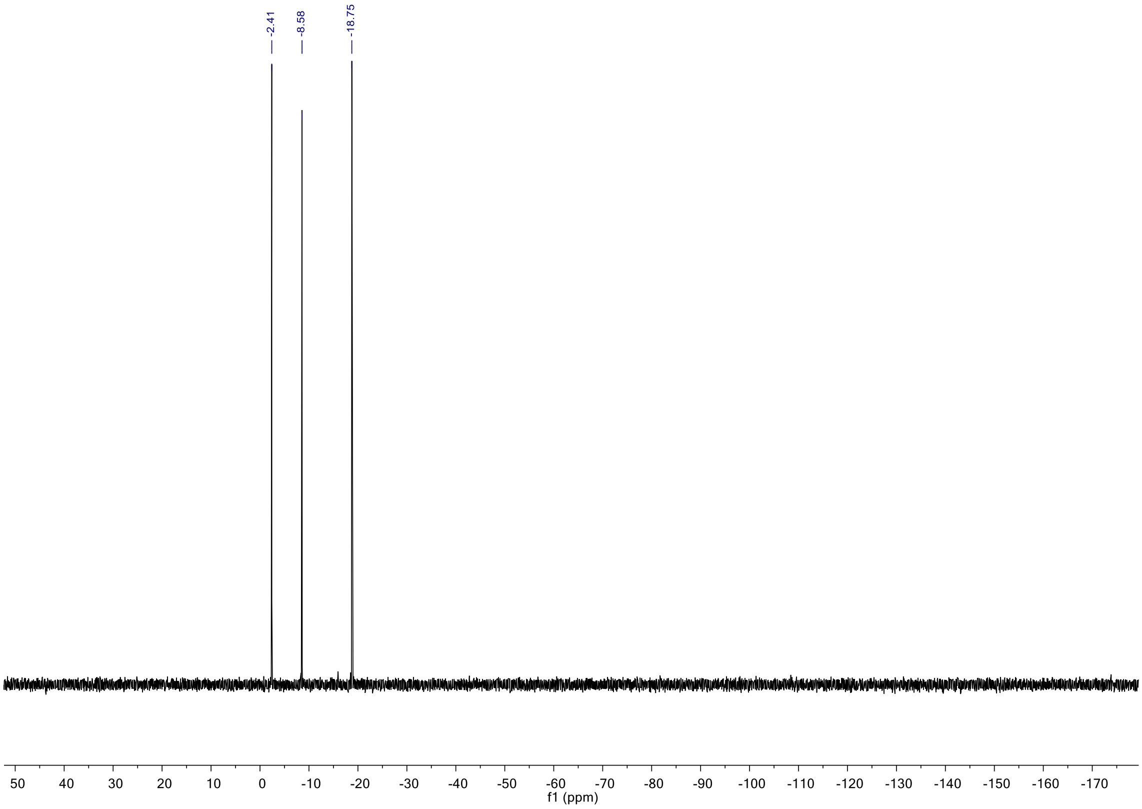

Figure S3. ^{29}Si NMR spectrum of **3a**.

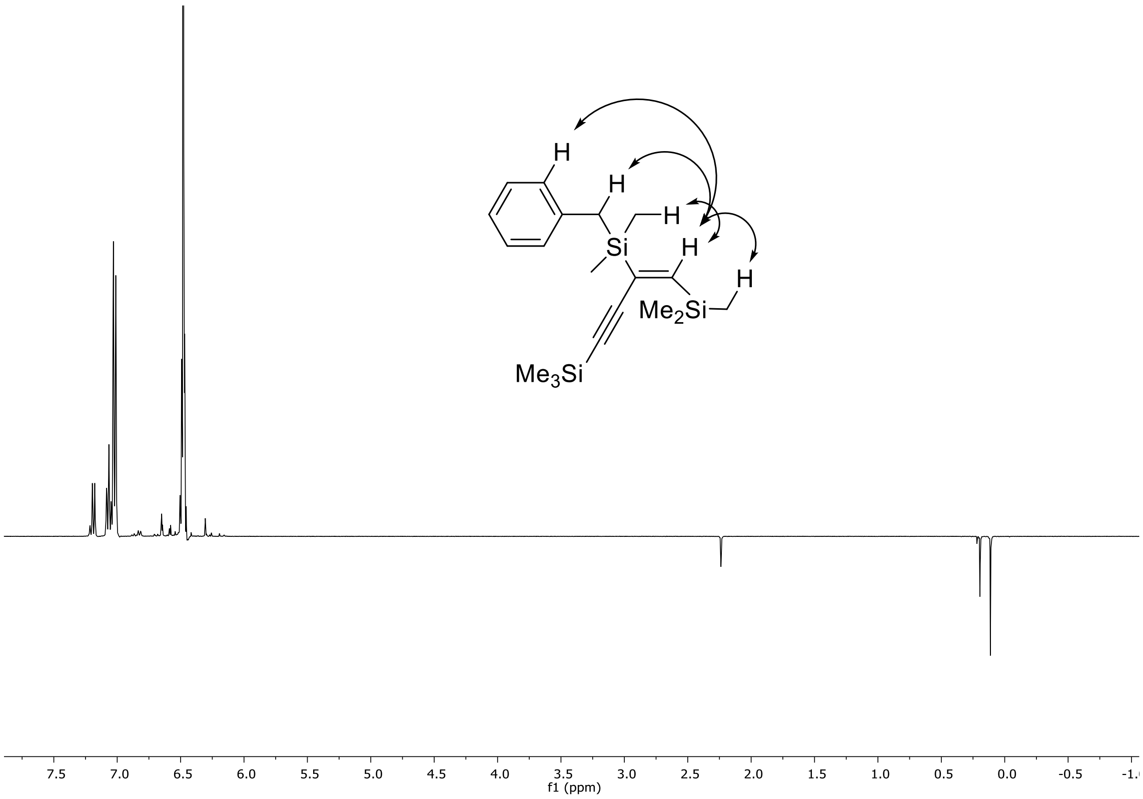

Figure S4. 1D selective gradient NOE NMR of compound **3a** directed at alkenyl proton at 6.48 ppm.

(*E*)-(2-(Triethylsilyl)but-1-en-3-yne-1,4-diyl)bis(trimethylsilane) (3b)

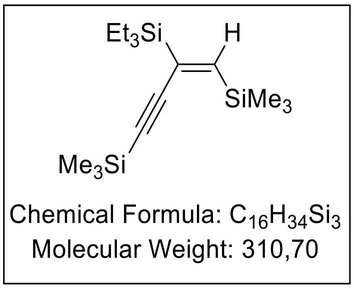

^{1}H NMR ($CDCl_3$, 300 MHz, δ, ppm): 6.53 (s, 1H, =C<u>H</u>), 0.96 (m, 9H, $CH_2C\underline{H}_3$), 0.66 (q, 6H, $J_{(H,H)}$= 7.93 Hz, $C\underline{H}_2CH_3$), 0.19 (s, 9H, $SiC\underline{H}_3$), 0.17 (s, 9H, $SiC\underline{H}_3$). **^{13}C NMR** ($CDCl_3$, 151 MHz, δ, ppm): 157.2 (=<u>C</u>H), 142.5 (<u>C</u>=CH), 107.9 (≡<u>C</u>SiMe3), 103.1 (<u>C</u>≡CSiMe3), 7.5 ($CH_2\underline{C}H_3$), 3.0 ($\underline{C}H_2CH_3$), -0.0, -0.8 ($Si\underline{C}H_3$). **^{29}Si NMR** ($CDCl_3$, 191 MHz, δ, ppm): 2.76 (<u>Si</u>Et3), -8.89 (<u>Si</u>Me3), -19.08 (<u>Si</u>Me3). **GC-MS** (EI, 70 eV) m/z (rel. int., %): 310.1 (M^+, 12.2), 282.1 (25.1), 281.1 (42.6), 254.1 (25.3), 253.0 (58.8), 225.0 (20.5), 184.1 (12.0), 183.0 (62.8), 179.0 (13.9), 168.9 (24.8), 164.9 (21.3), 154.9 (61.5), 126.9 (17.6), 114.9 (17.5), 86.9 (58.4), 72.9 (100.0), 58.9 (57.7). **FT-IR** (cm^{-1}): 2954, 2911, 2876, 1458, 1247, 1051, 835, 790, 735, 697, 630. Pale yellow oil. Isolated yield = 95% (151.8 mg). The compound **3b** has been previously described in the literature.[5-7]

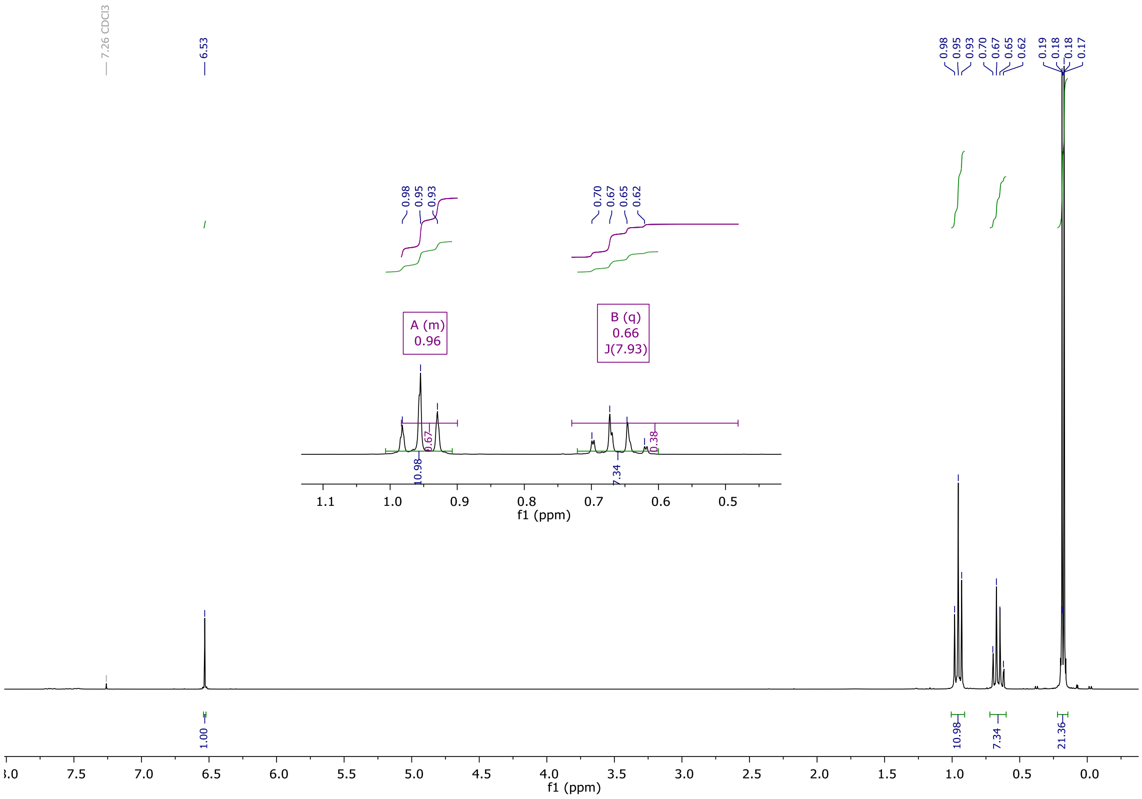

Figure S5. ^{1}H NMR spectrum of **3b**.

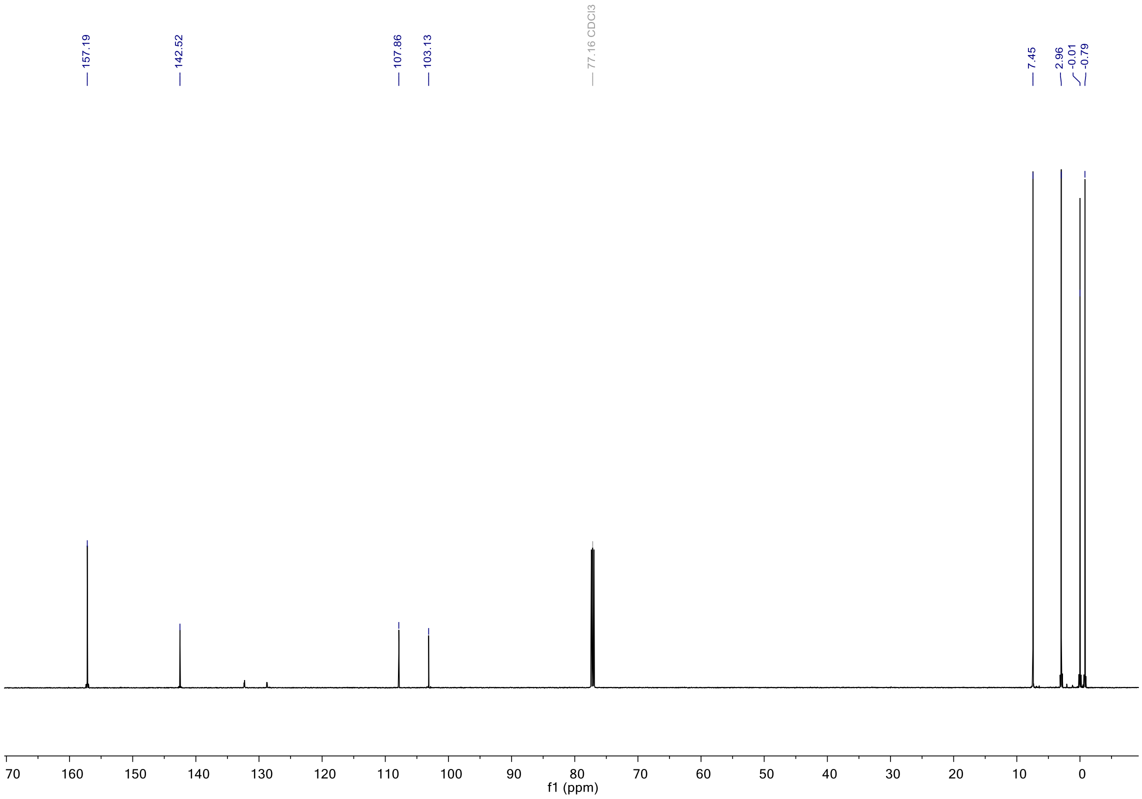

Figure S6. ^{13}C NMR spectrum of **3b**.

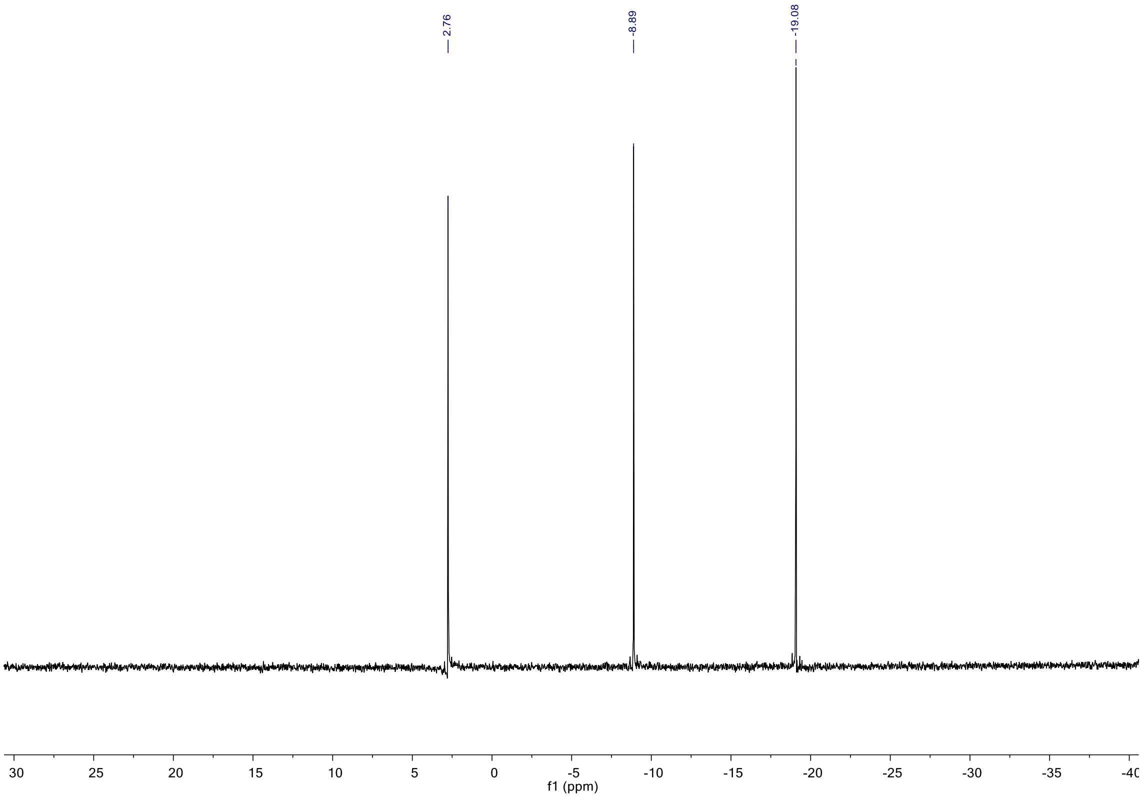

Figure S7. ^{29}Si NMR spectrum of **3b**.

(*E*)-(2-(Triisopropylsilyl)but-1-en-3-yne-1,4-diyl)bis(trimethylsilane) (3c)

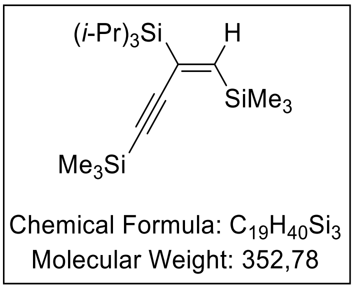

^{1}H NMR ($CDCl_3$, 300 MHz, δ, ppm): 6.55 (s, 1H, =CH), 1.19 – 1.31 (m, 3H, C(H)CH_3), 1.06 (s, 9H, C(H)CH_3), 1.08 (s, 9H, C(H)CH_3), 0.19 (s, 9H, SiCH_3), 0.15 (s, 9H, SiCH_3). **^{13}C NMR** ($CDCl_3$, 101 MHz, δ, ppm): 158.7 (=CH), 140.3 (C=CH), 108.9 (≡CSiMe$_3$), 102.8 (C≡CSiMe$_3$), 18.6 (C(H)CH_3), 11.0 (C(H)CH_3), -0.1, -0.8 (SiCH_3). **^{29}Si NMR** ($CDCl_3$, 191 MHz, δ, ppm): 1.54 (Si(*i*-Pr)$_3$), -9.03 (SiMe$_3$), -19.22 (SiMe$_3$). **GC-MS** (EI, 70 eV) m/z (rel. int., %): 351.9 ((M^+, 1.3), 310.0 (18.1), 308.9 (45.3), 268.0 (17.0), 267.0 (53.2), 225.0 (27.3), 212.1 (12.2), 211.0 (52.3), 183.0 (16.7), 169.0 (21.0), 154.9 (19.3), 140.9 (20.6), 86.9 (11.3), 72.9 (100.0), 58.9 (39.4). **FT-IR** (cm^{-1}): 2892, 2132, 1463, 1406, 1384, 1367, 1051, 1017, 996, 882, 788, 758, 739, 674, 630, 609, 562, 506, 488, 432, 425, 414. Colorless viscous oil. Isolated yield = 82% (148.8 mg). The compound **3c** has been previously described in the literature.[5-6]

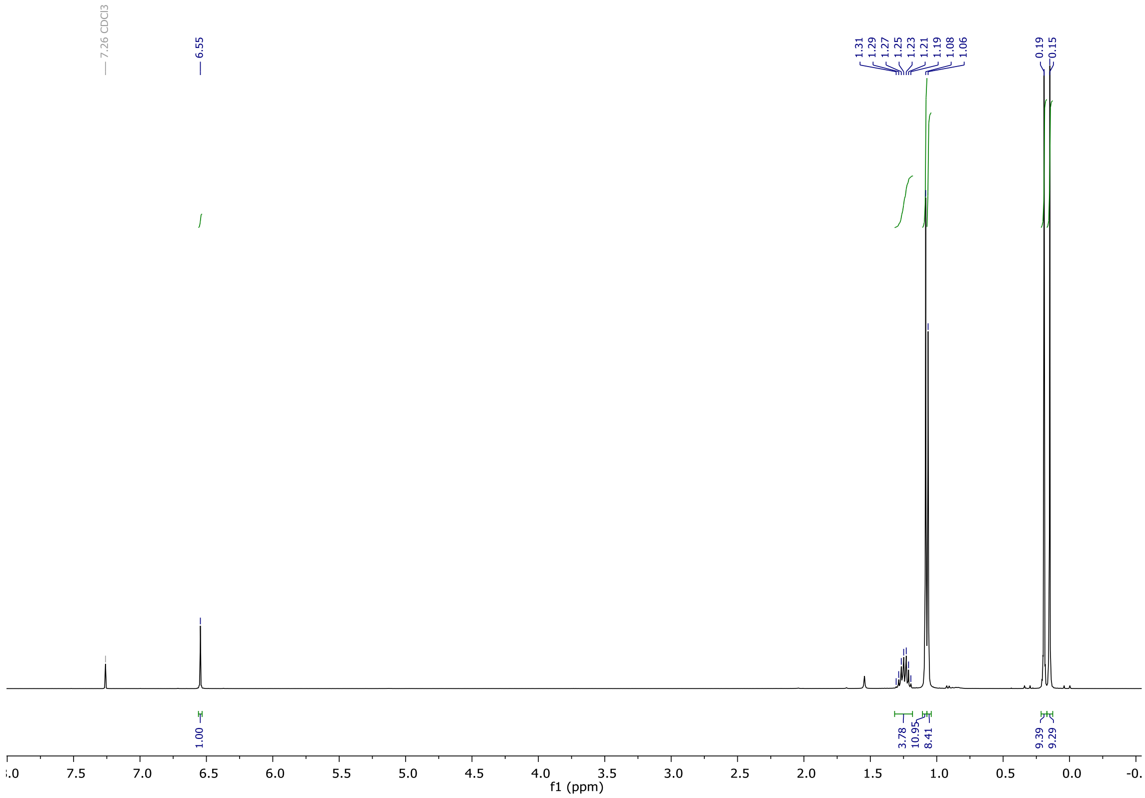

Figure S8. ^{1}H NMR spectrum of **3c**.

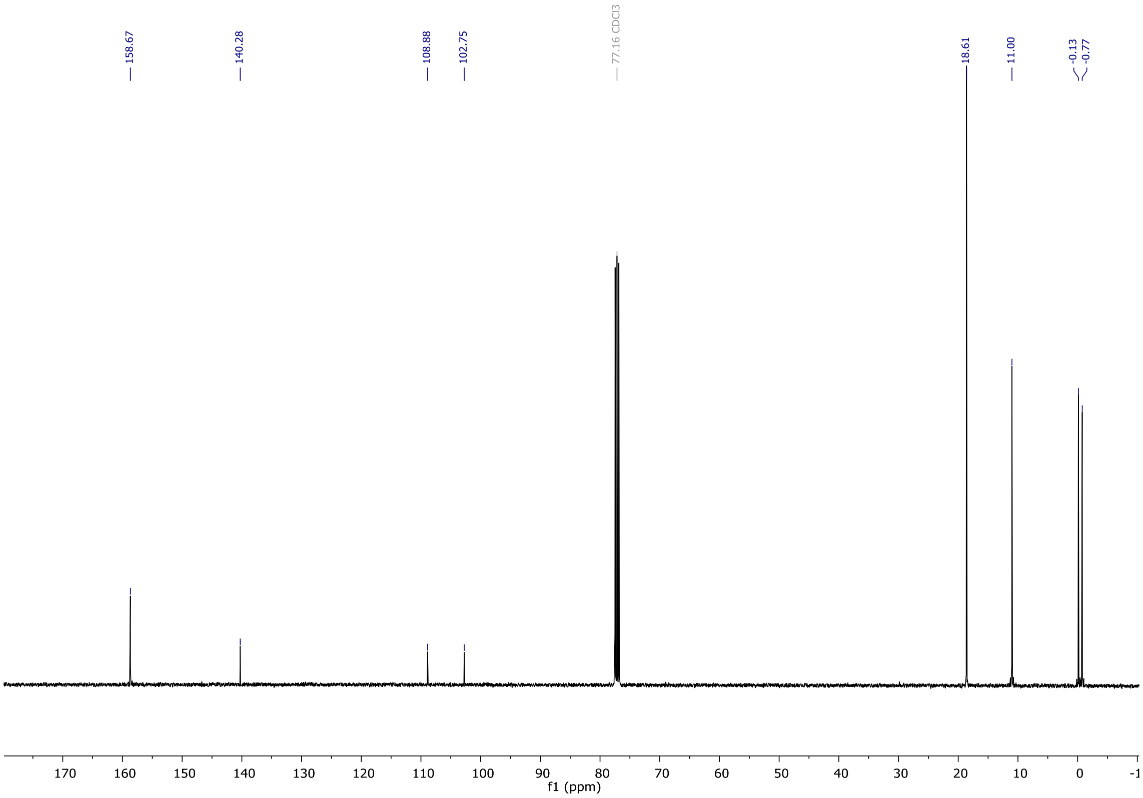

Figure S9. ^{13}C NMR spectrum of **3c**.

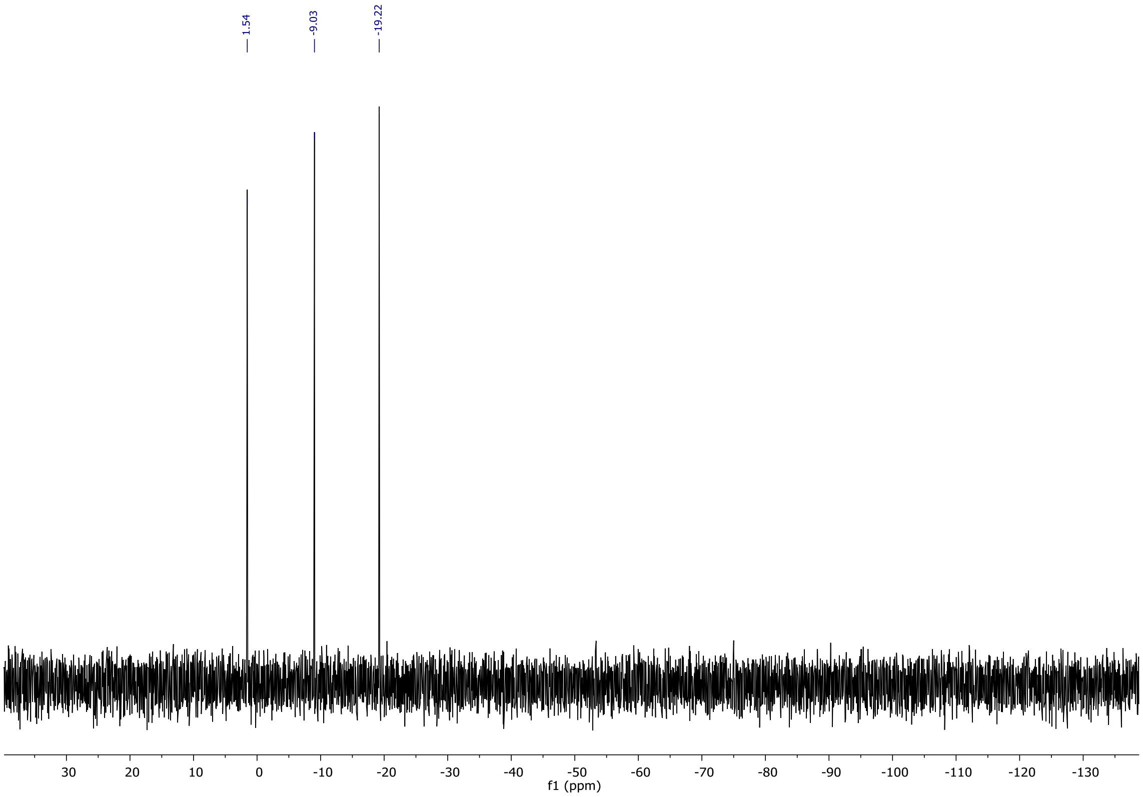

Figure S10. ^{29}Si NMR spectrum of **3c**.

(*E*)-(2-(Triethoxysilyl)but-1-en-3-yne-1,4-diyl)bis(trimethylsilane) (3d) – New compound

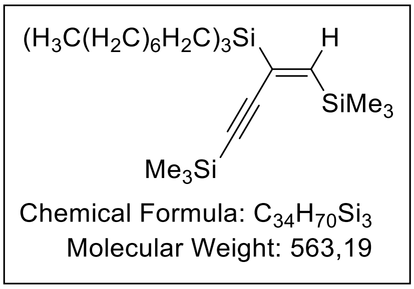

^{1}H NMR ($CDCl_3$, 300 MHz, δ, ppm): 6.53 (s, 1H, =CH), 1.28 - 1.31 (m, 36H, CH_2), 0.90 (t, 9H, $J_{(H,H)}$= 6.83 Hz, CH_2CH_3), 0.63 – 0.67 (m, 6H, CH_2), 0.19 (s, 9H, $SiCH_3$), 0.18 (s, 9H, $SiCH_3$). **^{13}C NMR** ($CDCl_3$, 101 MHz, δ, ppm): 156.8 (=CH), 143.6 (C=CH), 108.0 (≡CSiMe$_3$), 103.3 (C≡CSiMe$_3$), 33.9, 32.2, 29.4, 29.5, 23.8, 22.9 (CH_2), 14.3 (CH_2CH_3), 12.0 (CH_2), 0.0, -0.8 ($SiCH_3$).**^{29}Si NMR** ($CDCl_3$, 79 MHz, δ, ppm): -0.98 ($SiMe_3$), -8.95 ($SiMe_3$), -19.17 ($Si((CH_2)_7CH_3)_3$). **GC-MS** (EI, 70 eV) m/z (rel. int., %): 561.7 (M^+, 1.8), 339.4 (27.8), 338.3 (77.4), 337.1 (56.7), 227.7 (35.7), 226.7 (90.4), 225.4 (66.6), 143.7 (25.3), 99.0 (41.2), 97.7 (22.8), 85.6 (30.7), 84.6 (26.0), 73.7 (21.7)**,** 73.0 (100.0), 59.6 (36.6). **FT-IR** (cm^{-1}): 3048, 3022, 2999, 2897, 2134, 1486, 1428, 1190, 1049, 998, 789, 758, 631, 620, 485, 470, 457, 416, 410. **Elem. Anal.** calcd for $C_{34}H_{70}Si_3$: C, 72.51; H, 12.53; found C, 72.47; H, 12.50. Colorless oil. Isolated yield = 91% (263.6 mg).

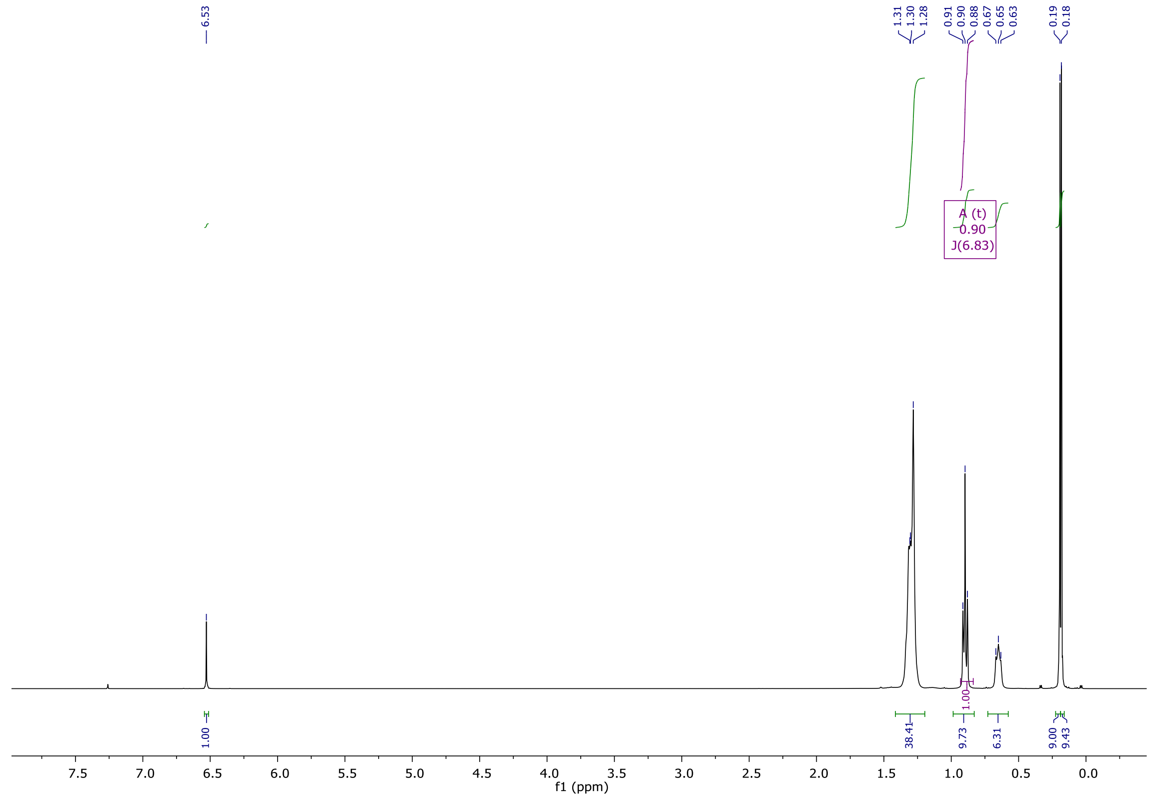

Figure S11. ^{1}H NMR spectrum of **3d**.

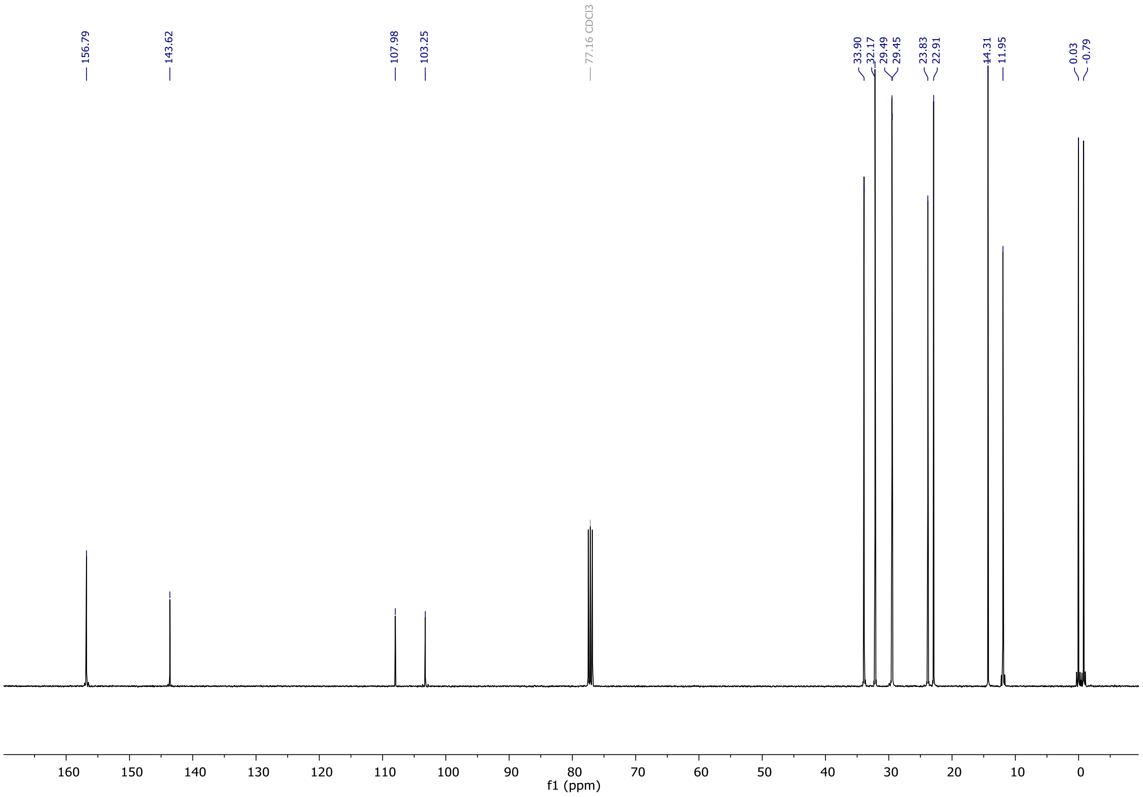

Figure S12. ^{13}C NMR spectrum of **3d**.

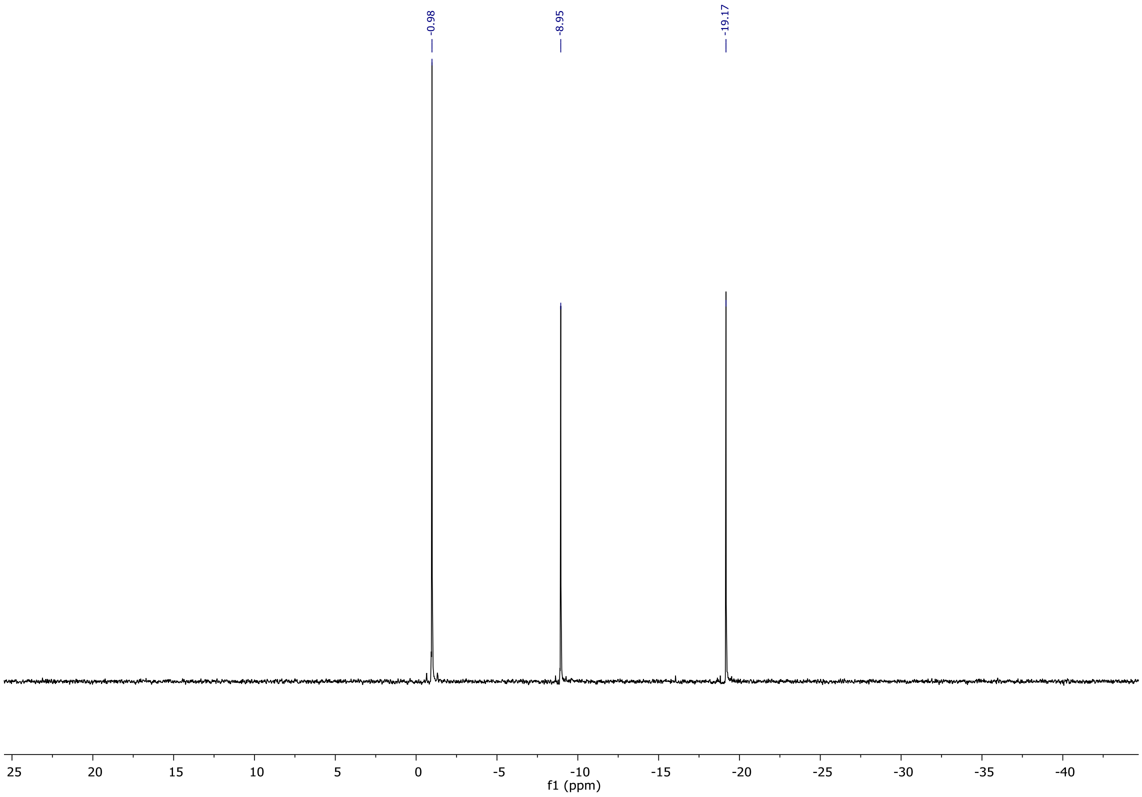

Figure S13. ^{29}Si NMR spectrum of **3d**.

(*E*)-(2-(Triethoxysilyl)but-1-en-3-yne-1,4-diyl)bis(trimethylsilane) (3e) – New compound

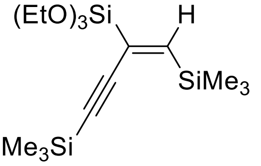

Chemical Formula: $C_{16}H_{34}O_3Si_3$
Molecular Weight: 358,70

^{1}H NMR ($CDCl_3$, 300 MHz, δ, ppm): 6.95 (s, 1H, =C<u>H</u>), 3.88 (q, 6H, $J_{(H,H)}$= 7.01 Hz, C<u>H</u>$_2$CH$_3$), 1.25 (t, 9H, $J_{(H,H)}$= 6.99 Hz, CH$_2$C<u>H</u>$_3$), 0.20 (s, 9H, SiC<u>H</u>$_3$), 0.17 (s, 9H, SiC<u>H</u>$_3$). **^{13}C NMR** ($CDCl_3$, 101 MHz, δ, ppm): 162.2 (=<u>C</u>H), 135.0 (<u>C</u>=CH), 106.4 (≡<u>C</u>SiMe$_3$), 102.9 (<u>C</u>≡CSiMe$_3$), 59.3 (<u>C</u>H$_2$CH$_3$), 18.3 (CH$_2$<u>C</u>H$_3$), -0.1, -1.0 (Si<u>C</u>H$_3$). **^{29}Si NMR** ($CDCl_3$, 79 MHz, δ, ppm): -8.01 (<u>Si</u>Me$_3$), -18.70 (<u>Si</u>Me$_3$), -85.80 (<u>Si</u>(OEt)$_3$). **GC-MS** (EI, 70 eV) m/z (rel. int., %): 358.0 (M$^+$, 2.7), 240.9 (13.7), 208.1 (10.6), 206.9 (66.4), 192.9 (12.6), 162.9 (38.2), 150.9 (10.3), 136.0 (15.0), 134.8 (20.3), 132.9 (13.2), 118.9 (20.7), 106.8 (13.7), 78.8 (30.0), 72.9 (100.0). **FT-IR** (cm^{-1}): 2957, 2899, 1247, 1073, 833, 756, 696, 630, 521. **Elem. Anal.** calcd for $C_{16}H_{34}O_3Si_3$: C, 53.58; H, 9.55; found C, 53.49; H, 9.51. Pale yellow oil. Isolated yield = 79% (145.8 mg).

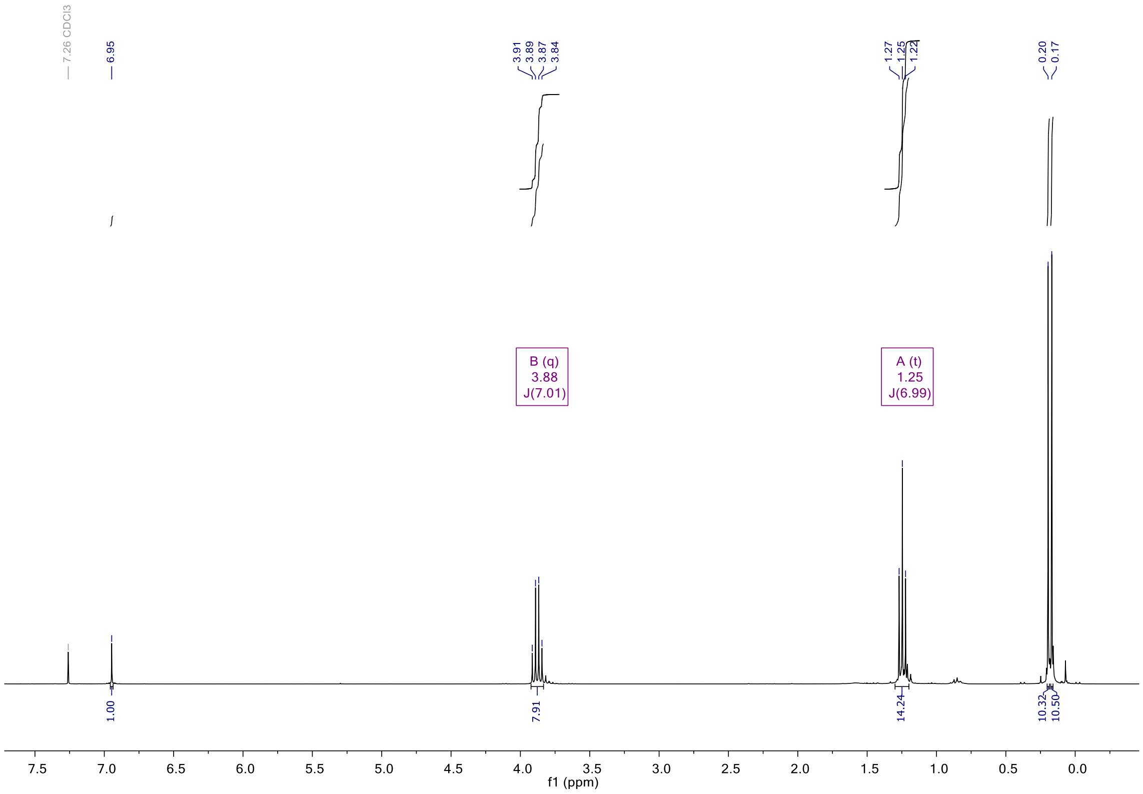

Figure S14. ^{1}H NMR spectrum of **3e**.

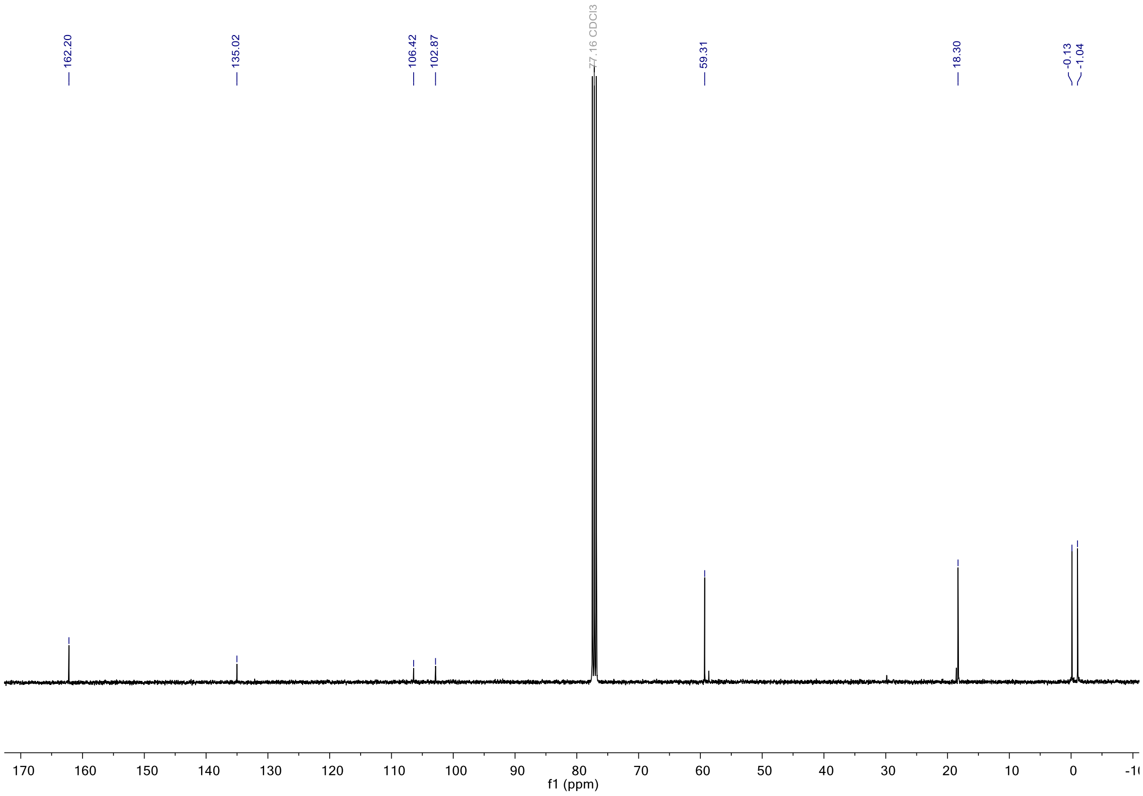

Figure S15. ^{13}C NMR spectrum of **3e**.

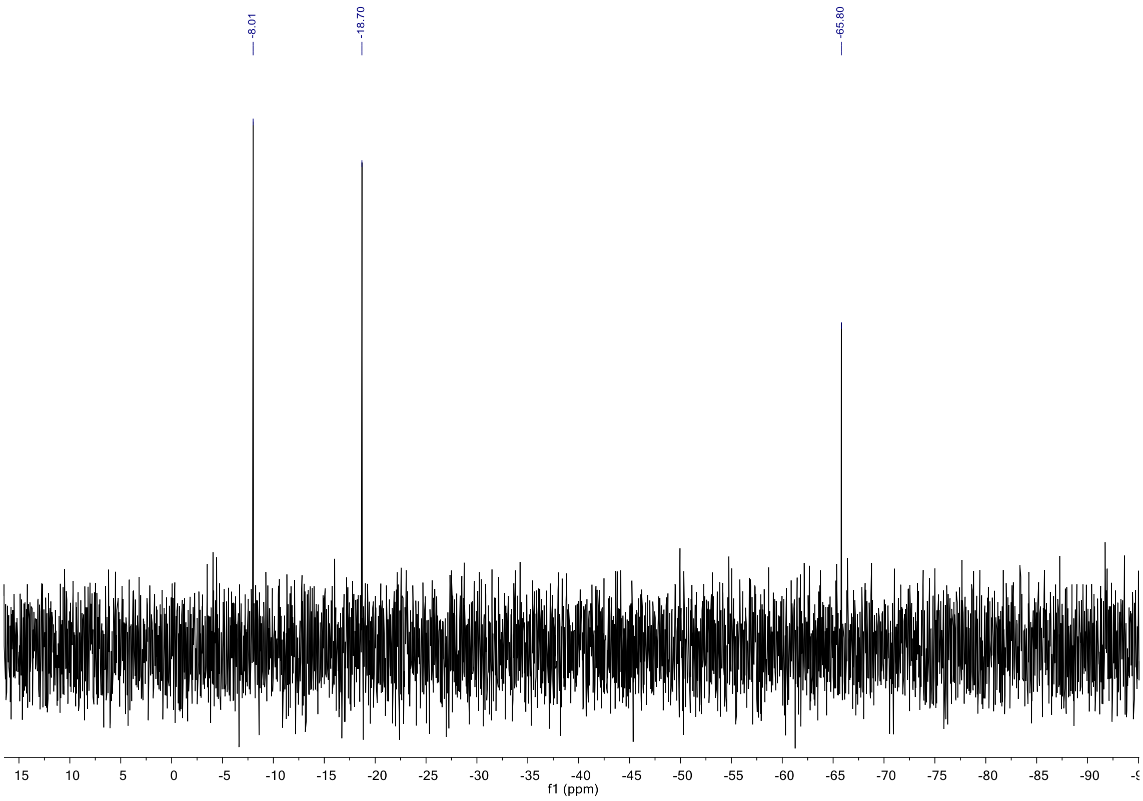

Figure S16. ^{29}Si NMR spectrum of **3e**.

(*E*)-3-(1,4-Bis(trimethylsilyl)but-1-en-3-yn-2-yl)-1,1,1,3,5,5,5-heptamethyltrisiloxane (3f) – New compound

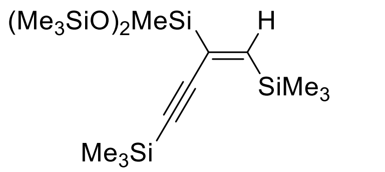

Chemical Formula: $C_{17}H_{40}O_2Si_5$
Molecular Weight: 416,93

^{1}H NMR ($CDCl_3$, 300 MHz, δ, ppm): 6.72 (s, 1H, =CH), 0.20 (s, 9H, $SiCH_3$), 0.19 (s, 9H, $SiCH_3$), 0.17 (s, 3H, $SiCH_3$), 0.13 (s, 18H, $OSiCH_3$). **^{13}C NMR** ($CDCl_3$, 101 MHz, δ, ppm): 157.3 (=CH), 143.2 (C=CH), 107.3 (≡$CSiMe_3$), 103.2 (C≡$CSiMe_3$), 1.9, 0.0, -1.0, -1.5 ($SiCH_3$). **^{29}Si NMR** ($CDCl_3$, 79 MHz, δ, ppm): 8.73 (($OSiMe_3$)$_3$), -8.52 ($SiMe_3$), -19.08 ($SiMe_3$), -40.04 ($SiMe(OSiMe_3)_2$). **GC-MS** (EI, 70 eV) m/z (rel. int., %): 415.8 (M^+, 6.5), 342.9 (15.2), 312.8 (15.1), 223.0 (11.6), 222.0 (20.2), 221.0 (85.9), 146.9 (10.3), 72.9 (100.0). **FT-IR** (cm^{-1}): 2899, 2137, 1407, 1249, 801, 788, 730, 695, 630, 592, 542, 445, 437, 420, 415. **Elem. Anal.** calcd for $C_{17}H_{40}O_2Si_5$: C, 48.97; H, 9.67; found C, 48.93; H, 9.65. Colorless oil. Isolated yield = 96% (205.9 mg).

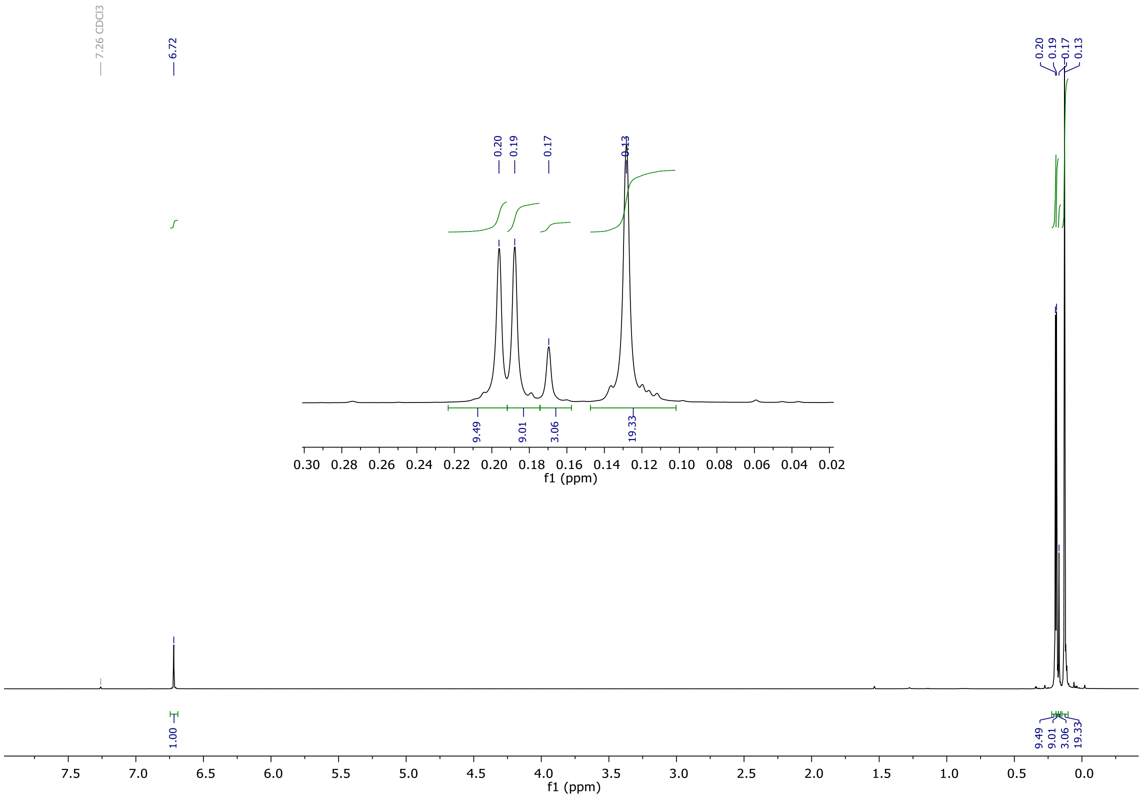

Figure S17. ^{1}H NMR spectrum of **3f**.

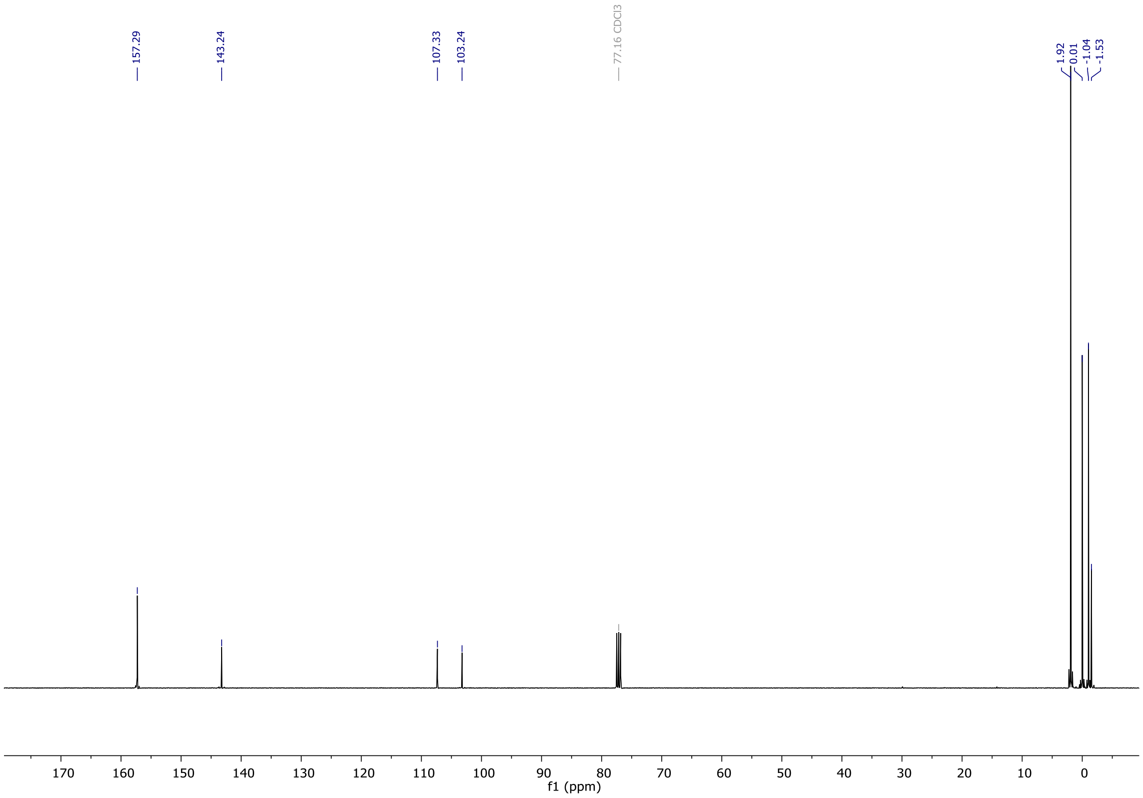

Figure S18. ^{13}C NMR spectrum of **3f**.

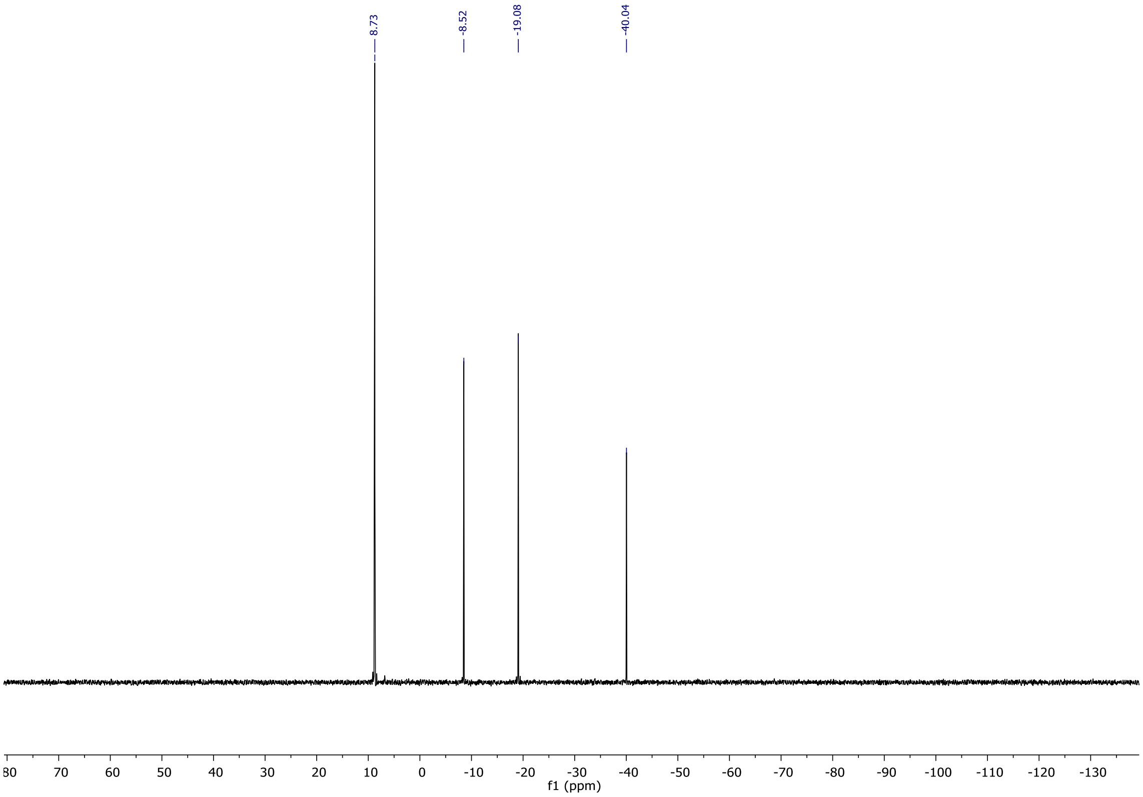

Figure S19. ^{29}Si NMR spectrum of **3f**.

(*E*)-3-(1,4-Bis(trimethylsilyl)but-1-en-3-yn-2-yl)-1,1,1,5,5,5-hexamethyl-3-((trimethylsilyl)oxy)trisiloxane (3g) – New compound

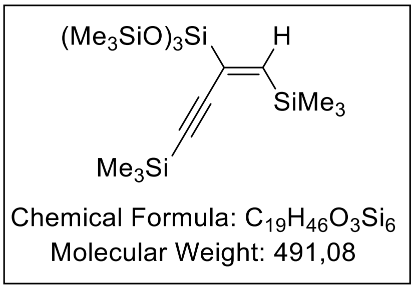

^{1}H NMR ($CDCl_3$, 300 MHz, δ, ppm): 6.79 (s, 1H, =C<u>H</u>), 0.19 (s, 9H, SiC<u>H</u>$_3$), 0.18 (s, 9H, SiC<u>H</u>$_3$), 0.14 (s, 27H, OSiC<u>H</u>$_3$). **^{13}C NMR** ($CDCl_3$, 75 MHz, δ, ppm): 159.0 (=<u>C</u>H), 139.7 (<u>C</u>=CH), 107.3 (≡<u>C</u>SiMe$_3$), 102.2 (<u>C</u>≡CSiMe$_3$), 1.8, -0.0, -1.2 (Si<u>C</u>H$_3$). **^{29}Si NMR** ($CDCl_3$, 80 MHz, δ, ppm): 9.12 ((O<u>Si</u>Me$_3$)$_3$), -8.70 (<u>Si</u>Me$_3$), -19.15 (<u>Si</u>Me$_3$), -84.59 (<u>Si</u>(OSiMe$_3$)$_3$). **GC-MS** (EI, 70 eV) m/z (rel. int., %): 490.0 (M^+, 2.7), 72.9 (100.0), 416.9 (8.8), 386.9 (5.1) 220.9 (6.4), 208.0 (6.3), 206.9 (29.4), 154.9 (9.4), 146.8 (12.2), 96.8 (5.6), 74.1 (6.5). **FT-IR** (cm^{-1}): 2958, 2899, 1249, 1056, 831, 753, 693, 630, 607, 559. **Elem. Anal.** calcd for $C_{19}H_{46}O_3Si_6$: C, 46.47; H, 9.44; found C, 46.60; H, 9.51. Pale yellow oil. Isolated yield = 90% (227.3 mg).

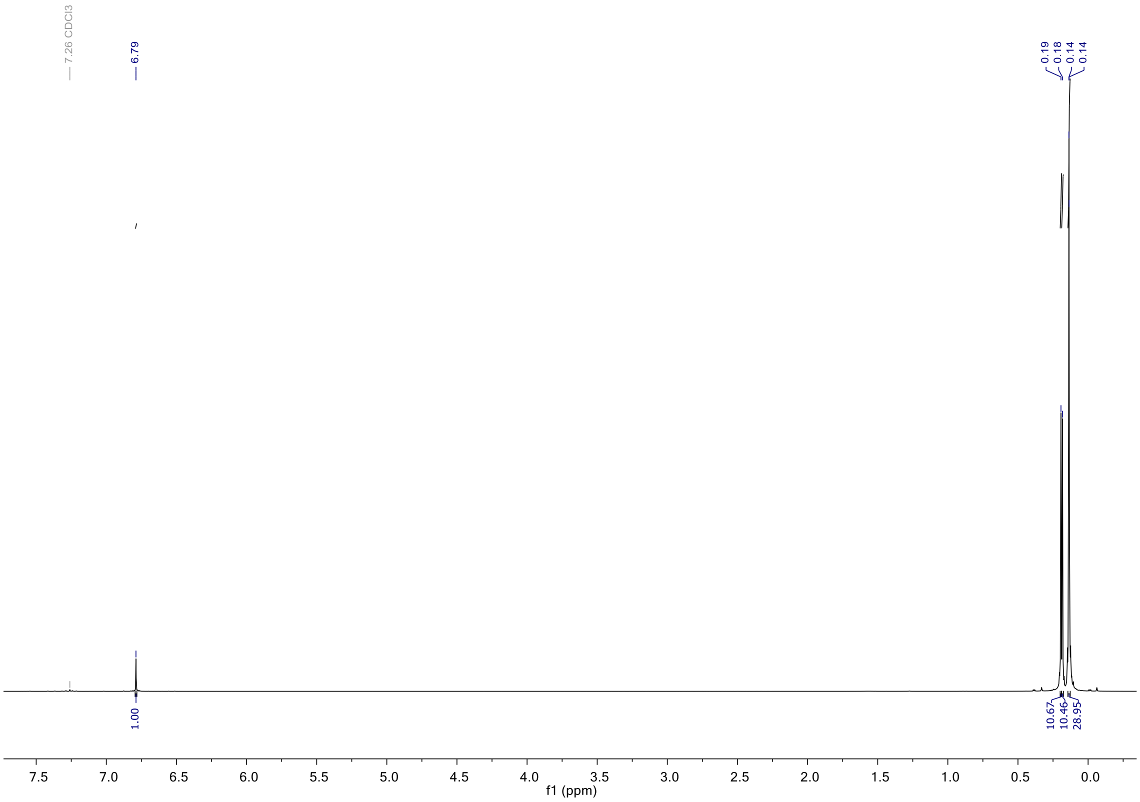

Figure S20. ^{1}H NMR spectrum of **3g**.

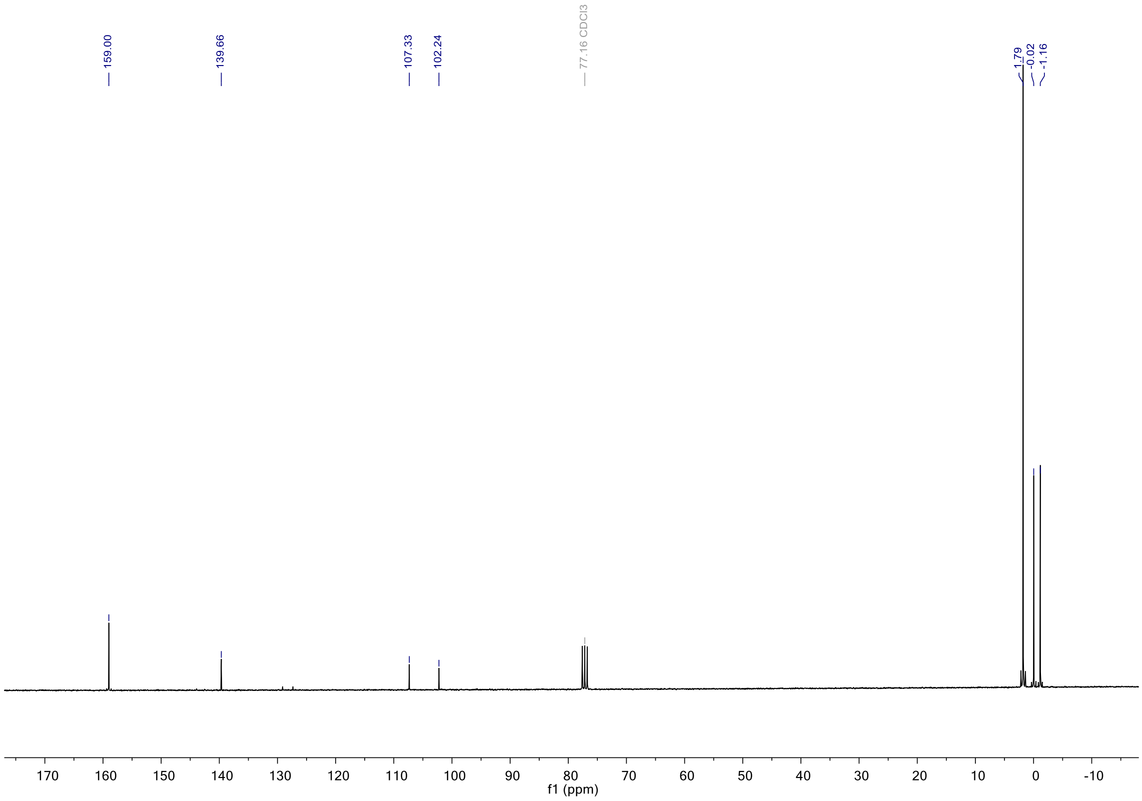

Figure S21. ^{13}C NMR spectrum of **3g**.

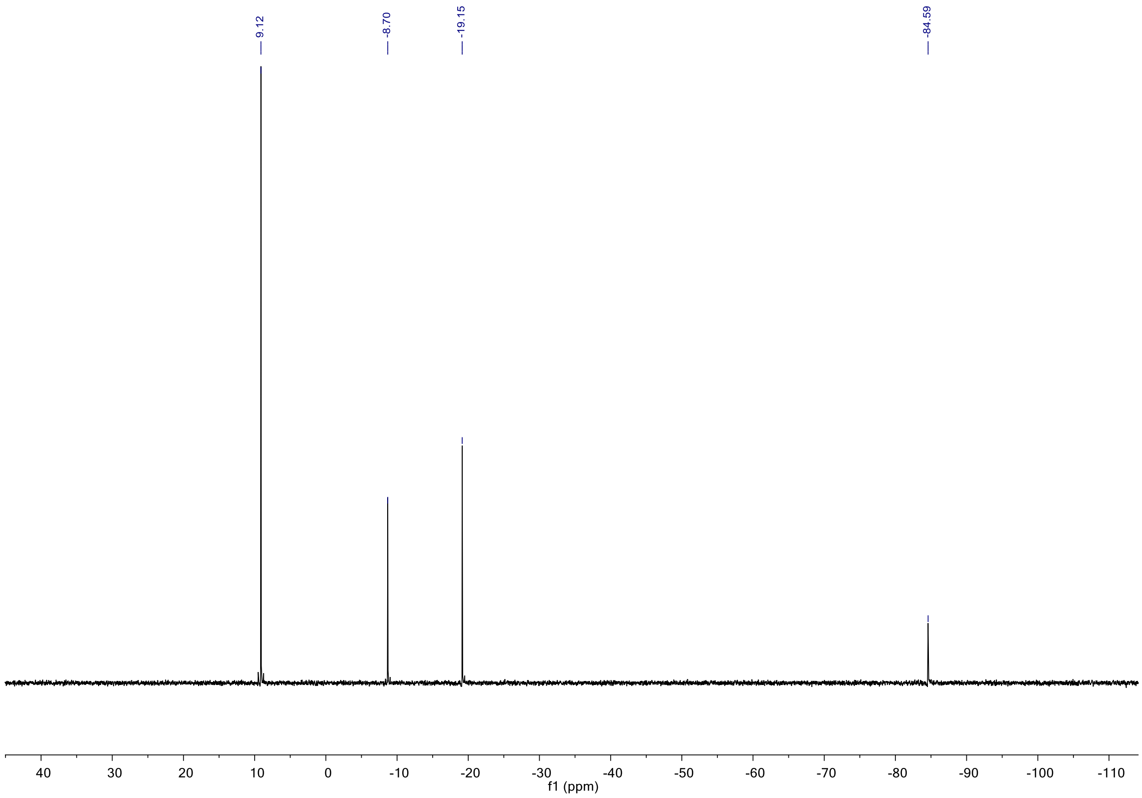

Figure S22. ^{29}Si NMR spectrum of **3g**.

(*E*)-(2-(Diphenylsilyl)but-1-en-3-yne-1,4-diyl)bis(trimethylsilane) (3h)

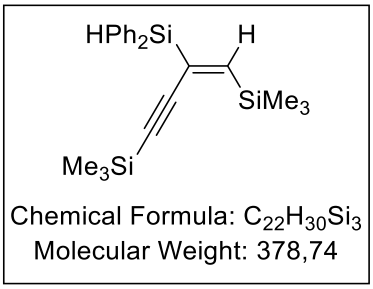

^{1}H NMR ($CDCl_3$, 300 MHz, δ, ppm): 7.66 - 7.38 (m, 10H, Ph), 6.88 (s, 1H, =C<u>H</u>), 5.12 (s, 1H, Si<u>H</u>), 0.22 (s, 9H, SiC<u>H</u>$_3$), 0.12 (s, 9H, SiC<u>H</u>$_3$). **^{13}C NMR** ($CDCl_3$, 101 MHz, δ, ppm): 162.2 (=<u>C</u>H), 138.1 (<u>C</u>=CH), 136.0, 135.8, 133.0, 130.0, 130.0, 128.2, 128.0 (Ph), 106.9 (≡<u>C</u>SiMe$_3$), 105.2 (<u>C</u>≡CSiMe$_3$), -0.2, -0.9 (Si<u>C</u>H$_3$). **^{29}Si NMR** ($CDCl_3$, 79 MHz, δ, ppm): -7.80 (<u>Si</u>Me$_3$), -16.69 (<u>Si</u>HPh$_2$), -18.52 (<u>Si</u>Me$_3$). **GC-MS** (EI, 70 eV) m/z (rel. int., %): 378.0 (M^+, 3.0), 363.0 (11.2), 305.0 (17.2), 290.0 (31.5), 288.8 (11.2), 285.0 (11.3), 228.0 (11.1), 227.0 (13.7), 196.9 (12.8), 182.9 (28.4), 180.9 (20.4), 154.9 (10.6), 134.9 (45.1), 104.8 (36.8), 72.9 (100.0). **FT-IR** (cm^{-1}): 3069, 3050, 2956, 2897, 2131, 1428, 1247, 1108, 1052, 835, 796, 758, 729, 694, 629, 580, 536, 518, 482. Pale yellow oil. Isolated yield = 76% (194.8 mg). The compound **3h** has been previously described in the literature.[5, 6 and 8] However, no NMR spectra were provided.

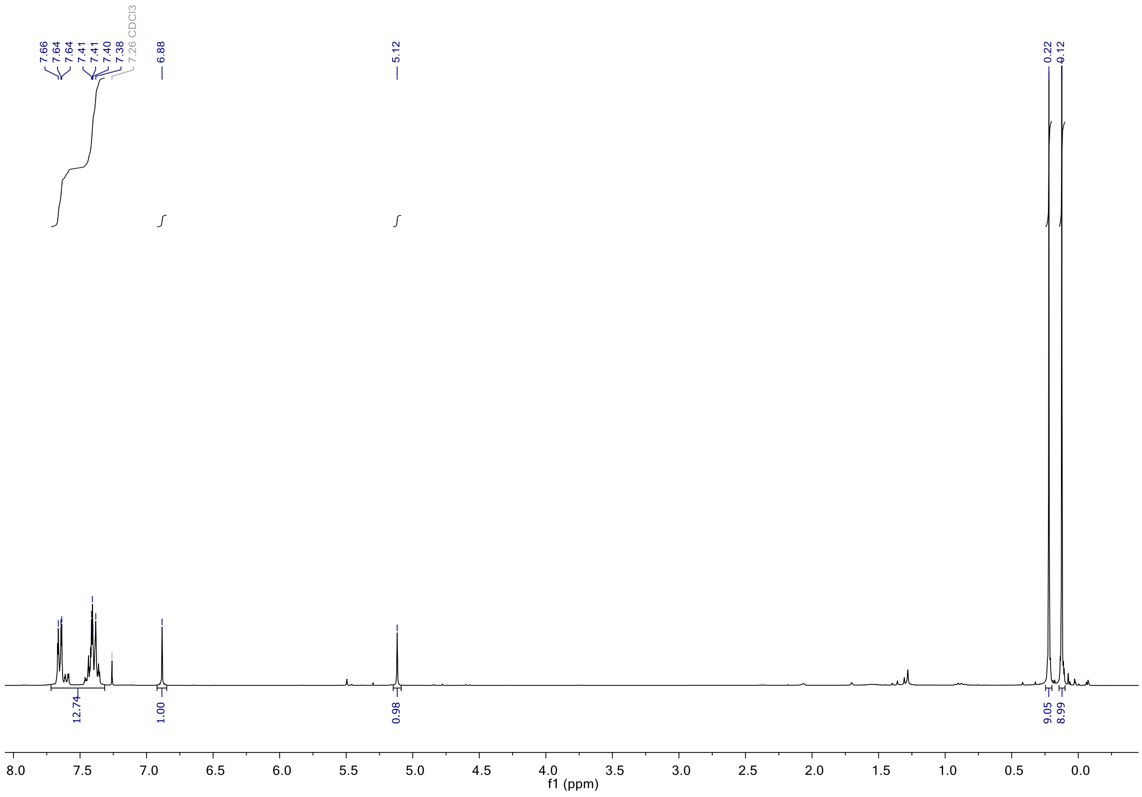

Figure S23. ^{1}H NMR spectrum of **3h**.

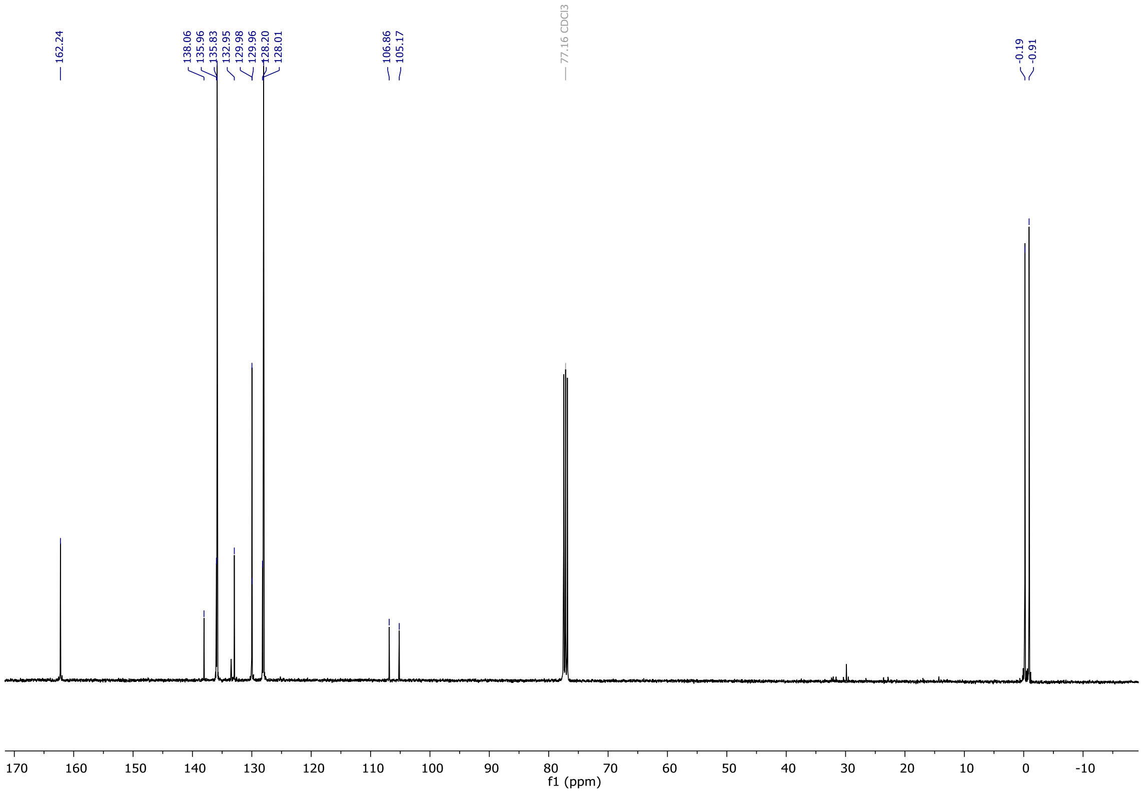

Figure S24. ^{13}C NMR spectrum of **3h**.

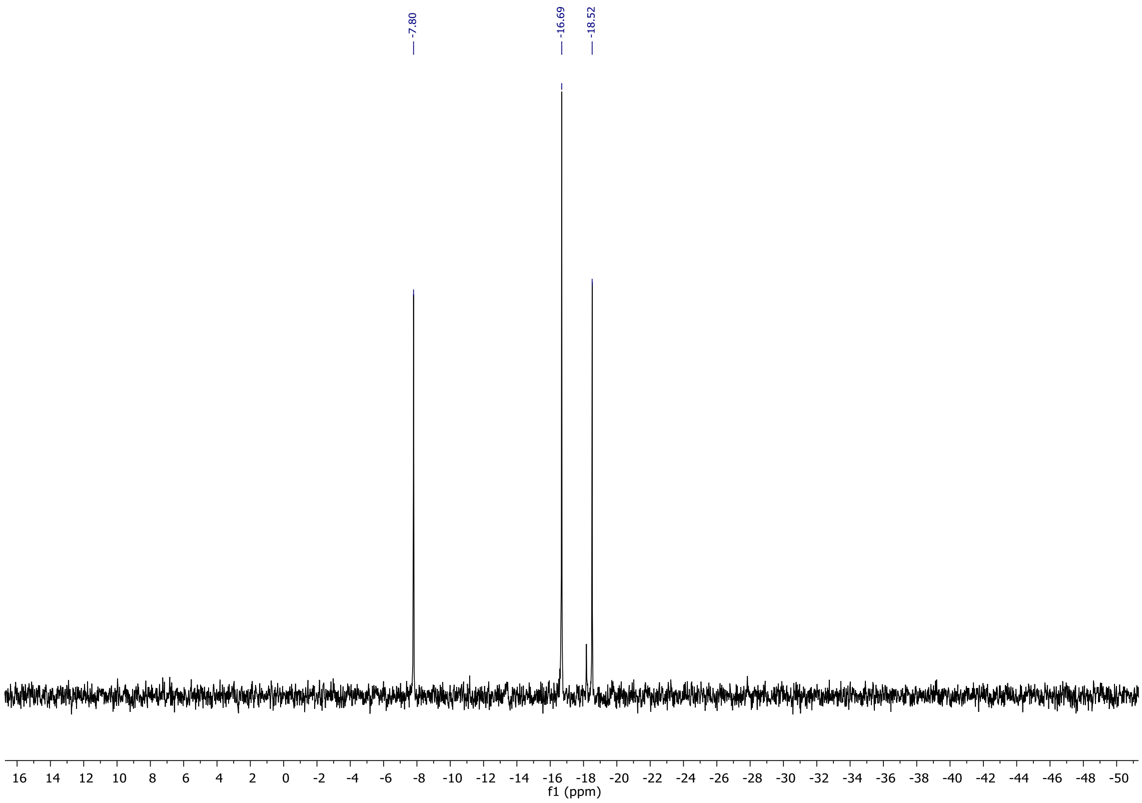

Figure S25. ^{29}Si NMR spectrum of **3h**.

(*E*)-(2-(Triphenylsilyl)but-1-en-3-yne-1,4-diyl)bis(trimethylsilane) (3i) – New compound

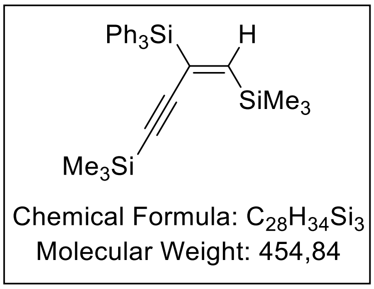

^{1}H NMR ($CDCl_3$, 300 MHz, δ, ppm): 7.71 - 7.42 (m, 15H, Ph), 6.86 (s, 1H, =CH), 0.31 (s, 9H, $SiCH_3$), 0.17 (s, 9H, $SiCH_3$). **^{13}C NMR** ($CDCl_3$, 101 MHz, δ, ppm): 162.7 (=CH), 139.2 (C=CH), 136.6, 133.5, 129.8, 127.8 (Ph), 108.0 (≡CSiMe$_3$), 105.4 (C≡CSiMe$_3$), -0.3, -0.8 ($SiCH_3$). **^{29}Si NMR** ($CDCl_3$, 79 MHz, δ, ppm): -7.92 ($SiMe_3$), -15.85 ($SiHPh_2$), -18.66 ($SiMe_3$). **GC-MS** (EI, 70 eV) m/z (rel. int., %): 454.1 (M^+, 5.7), 304.4 (15.1), 303.0 (13.5), 260.6 (23.4), 259.0 (100.0), 181.5 (33.0), 180.3 (23.6), 135.6 (10.0), 73.6 (13.1), 73.0 (24.1). **FT-IR** (cm^{-1}): 2956, 2872, 2130, 1467, 1408, 1378, 1175, 1050, 792, 698, 630, 513, 486, 477, 443, 434, 420. **Elem. Anal.** calcd for $C_{28}H_{34}Si_3$: C, 73.94; H, 7.54; found C, 73.89; H, 7.52. Colorless viscous oil. Isolated yield = 89% (208.2 mg).

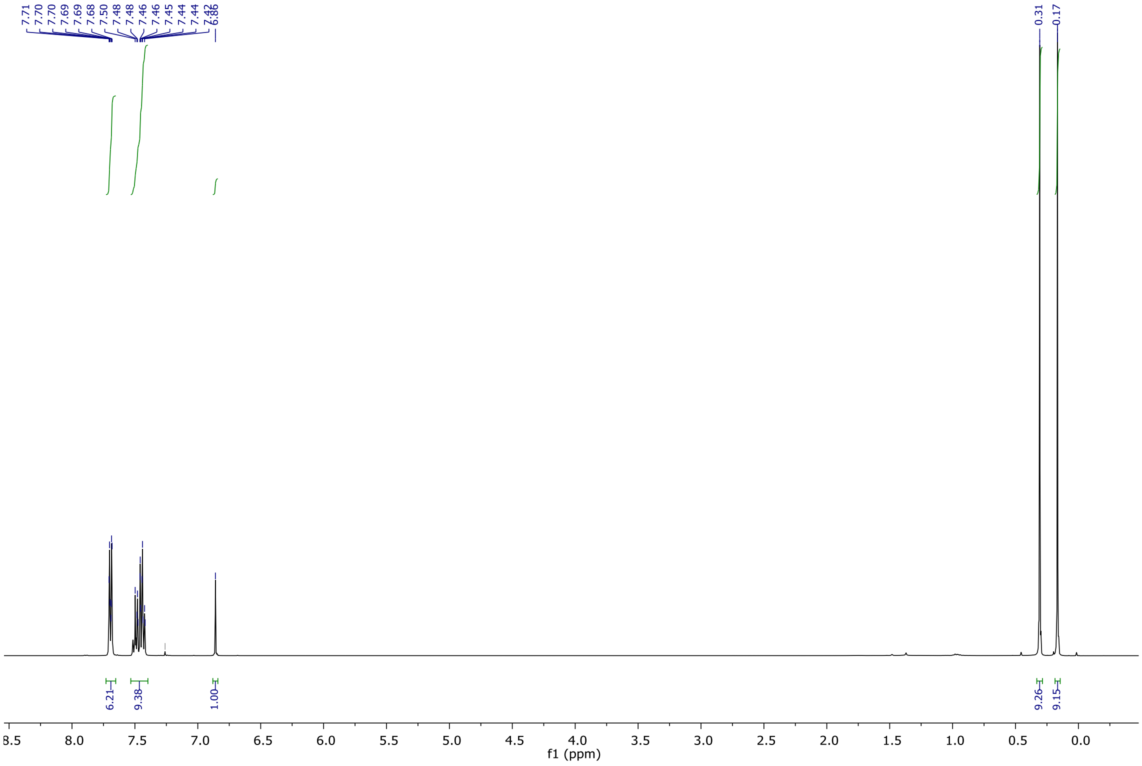

Figure S26. ^{1}H NMR spectrum of **3i**.

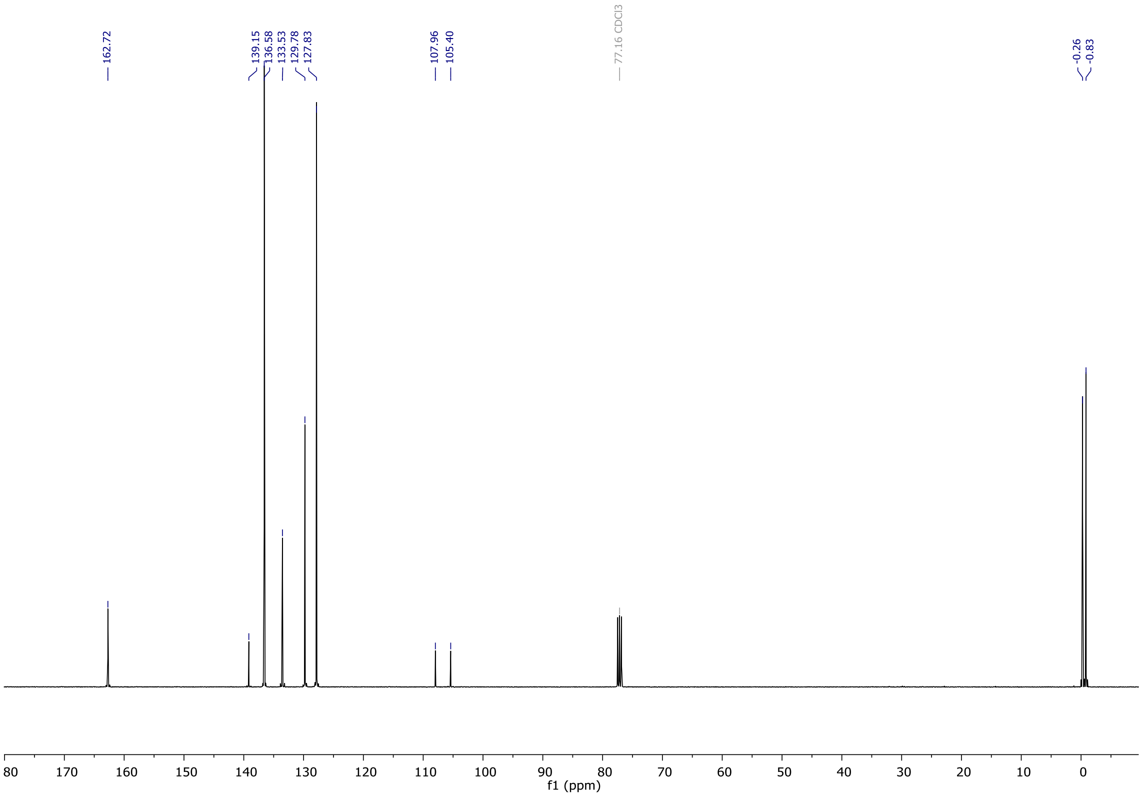

Figure S27. ^{13}C NMR spectrum of **3i**.

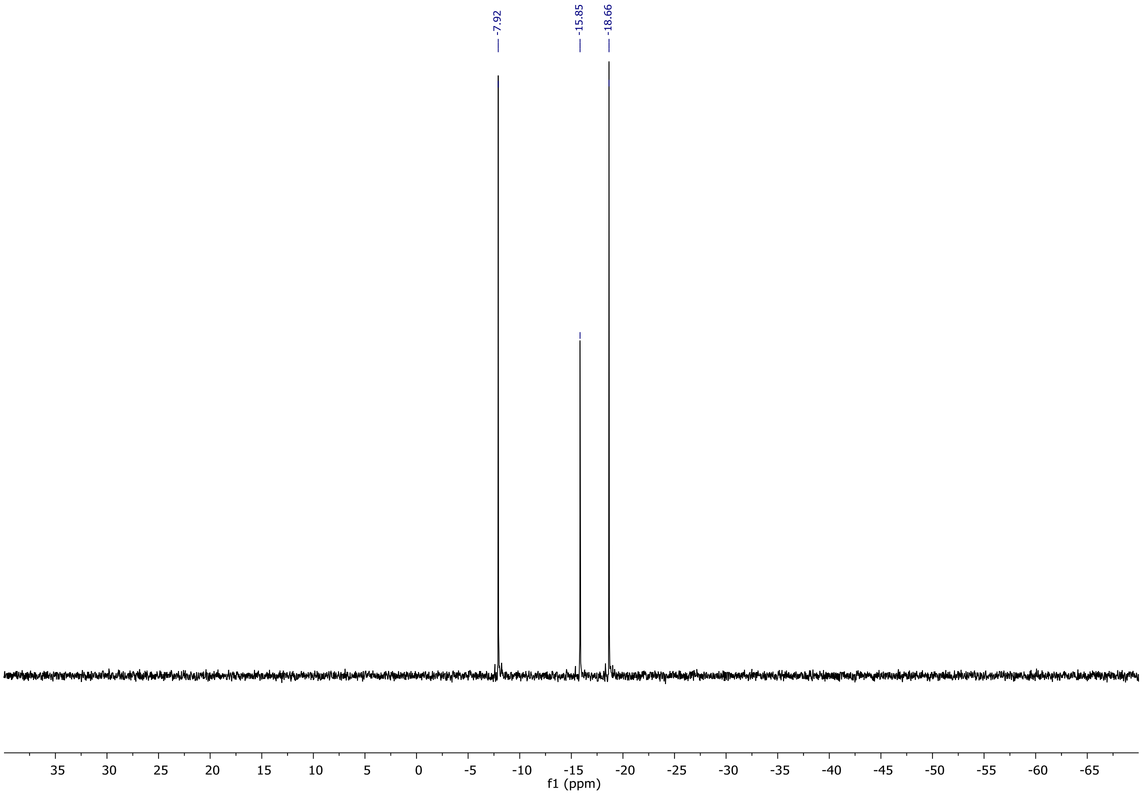

Figure S28. ^{29}Si NMR spectrum of **3i**.

(*E*)-(2-(Chlorodimethylsilyl)but-1-en-3-yne-1,4-diyl)bis(trimethylsilane) (3j) – New compound

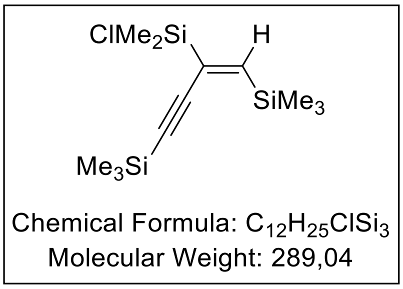

^{1}H NMR ($CDCl_3$, 300 MHz, δ, ppm): 6.86 (s, 1H, =CH), 0.54 (s, 6H, Si(CH_3)$_2$Cl), 0.21 (s, 9H, SiCH_3), 0.20 (s, 9H, SiCH_3). **^{13}C NMR** ($CDCl_3$, 101 MHz, δ, ppm): 158.9 (=CH), 140.2 (C=CH), 105.3 (≡CSiMe$_3$), 105.1 (C≡CSiMe$_3$), 1.1 (Si(CH_3)$_2$Cl), -0.1, -1.0 (SiCH_3). **^{29}Si NMR** ($CDCl_3$, 79 MHz, δ, ppm): 16.96 (SiMe$_2$Cl), -7.40 (SiMe$_3$), -18.26 (SiMe$_3$). **GC-MS** (EI, 70 eV) m/z (rel. int., %): 289.7 (M^+, 9.5), 288.2 (16.7), 181.7 (19.2), 180.6 (85.5), 179.6 (73.8), 166.7 (21.4), 165.6 (100.0), 164.6 (94.0), 156.7 (12.0), 155.6 (32.3), 155.0 (30.1), 97.5 (11.1), 96.8 (13.8), 93.5 (12.1), 73.6 (26.2), 72.9 (85.8). **Elem. Anal.** calcd for $C_{12}H_{25}ClSi_3$: C, 49.87; H, 8.72; found C, 49.96; H, 8.76. Pale yellow oil. Isolated yield = 82% (148.7 mg).

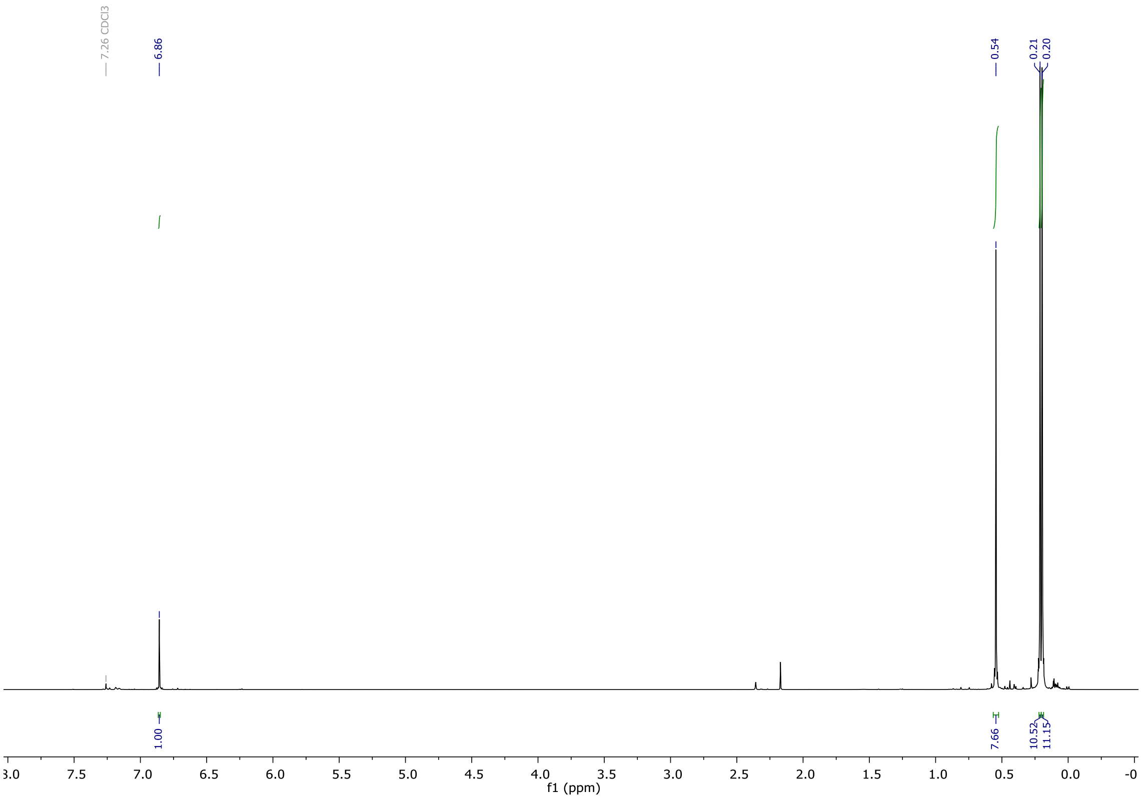

Figure S29. ^{1}H NMR spectrum of **3j**.

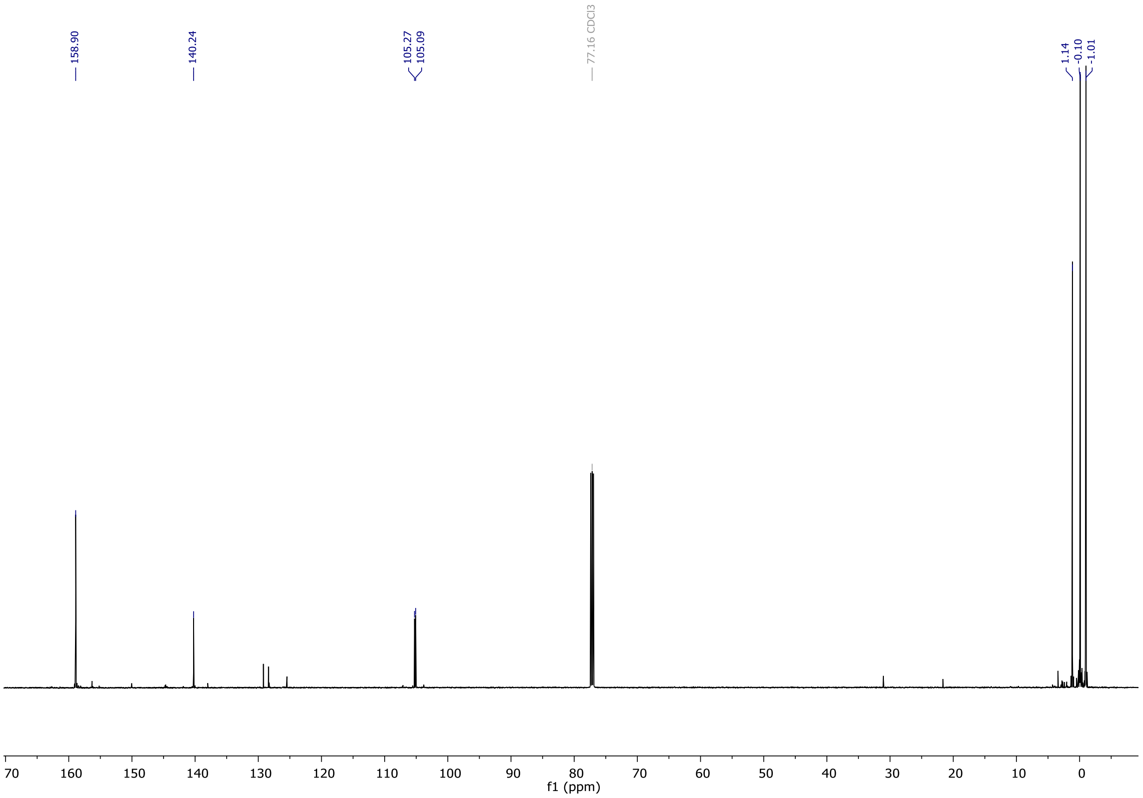

Figure S30. ^{13}C NMR spectrum of **3j**.

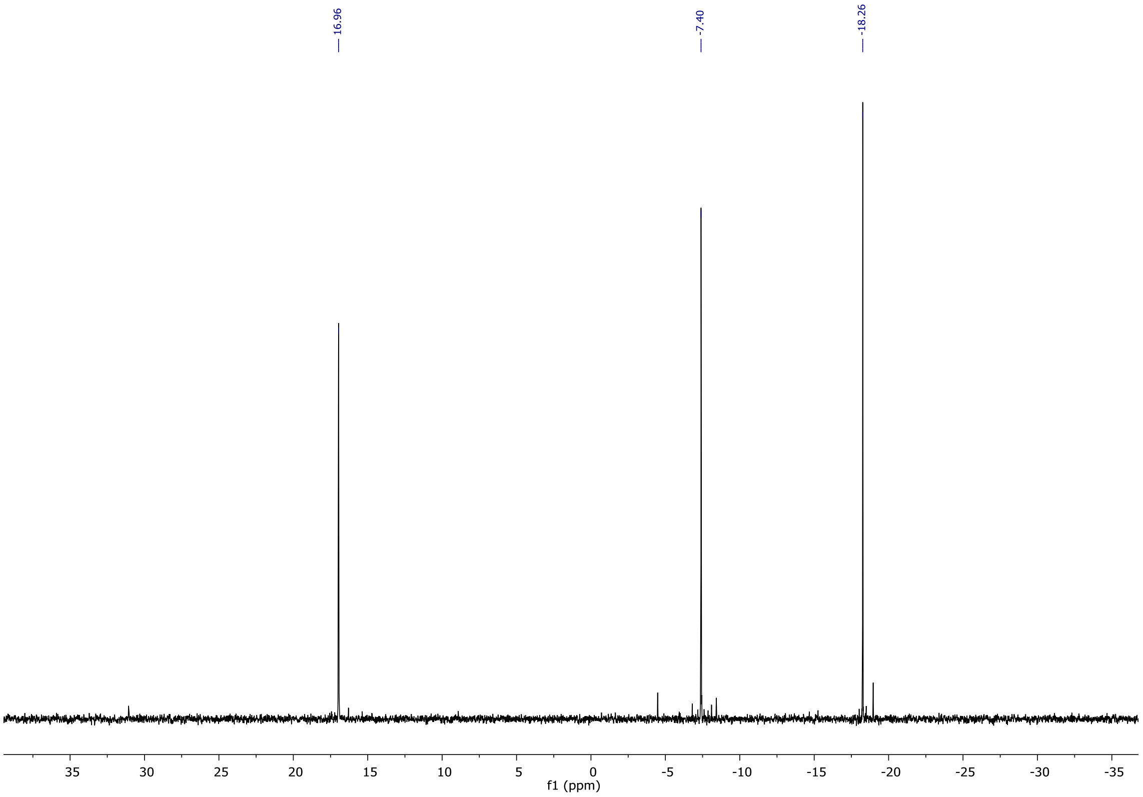

Figure S31. ^{29}Si NMR spectrum of **3j**.

(*E*)-Benzyldimethyl(1-(trimethylsilyl)but-1-en-3-yn-2-yl)silane (5) – New compound

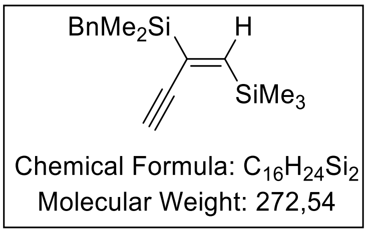

^{1}H NMR ($CDCl_3$, 400 MHz, δ, ppm): 7.21-7.01 (m, 5H, Ph), 6.55 (d, 1H, $J_{(H,H)}$= 0.93 Hz, =C<u>H</u>), 3.52 (d, 1H, $J_{(H,H)}$= 0.99 Hz, ≡C<u>H</u>), 2.25 (s, 2H, C<u>H</u>$_2$Ph), 0.20 (s, 9H, SiC<u>H</u>$_3$), 0.13 (s, 6H, SiC<u>H</u>$_3$). **^{13}C NMR** ($CDCl_3$, 101 MHz, δ, ppm): 158.5 (=<u>C</u>H), 142.1 (<u>C</u>=CH), 139.6, 128.5, 128.2, 124.2 (Ph), 86.9 (≡<u>C</u>H), 85.4 (<u>C</u>≡CH), 24.8 (<u>C</u>H$_2$Ph), -0.9, -4.3 (Si<u>C</u>H$_3$). **^{29}Si NMR** ($CDCl_3$, 79 MHz, δ, ppm): -1.75 (<u>Si</u>Me$_2$Bn), -8.60 (<u>Si</u>Me$_3$). **GC-MS** (EI, 70 eV) m/z (rel. int., %): 272.0 (M^+, 0.9), 199.1 (4.8), 197.9 (13.4), 184.0 (6.3), 183.0 (8.4), 182.0 (7.0), 180.9 (31.2), 148.8 (9.4), 120.8 (20.8), 90.8 (10.6), 82.8 (23.9), 74.1 (8.6), 72.8 (100). **FT-IR** (cm^{-1}): 3308, 3025, 2955, 2897, 1600, 1493, 1452, 1408, 1246, 1154, 1067, 822, 794, 760, 696, 612, 477, 449. **Elem. Anal.** calcd for $C_{16}H_{24}Si_2$: C, 70.51; H, 8.88; found C, 70.60; H, 8.92. Colorless oil. Isolated yield = 96% (227.7 mg).

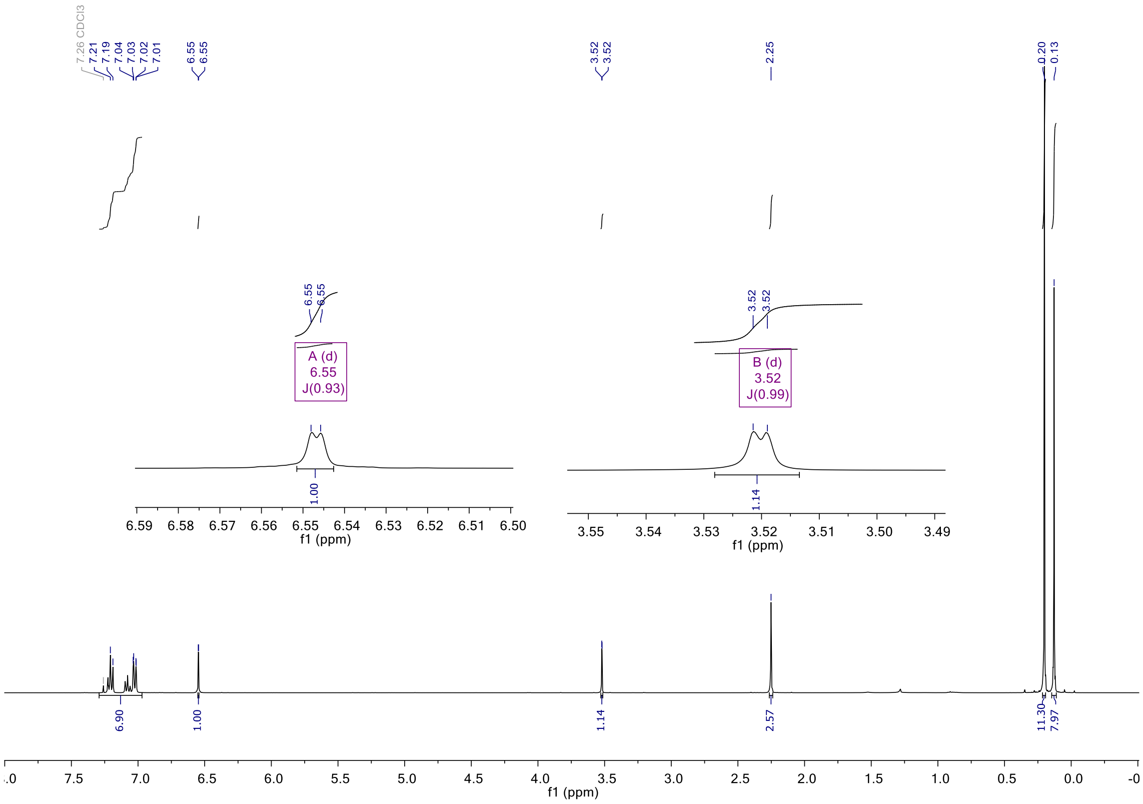

Figure S32. ^{1}H NMR spectrum of **5**.

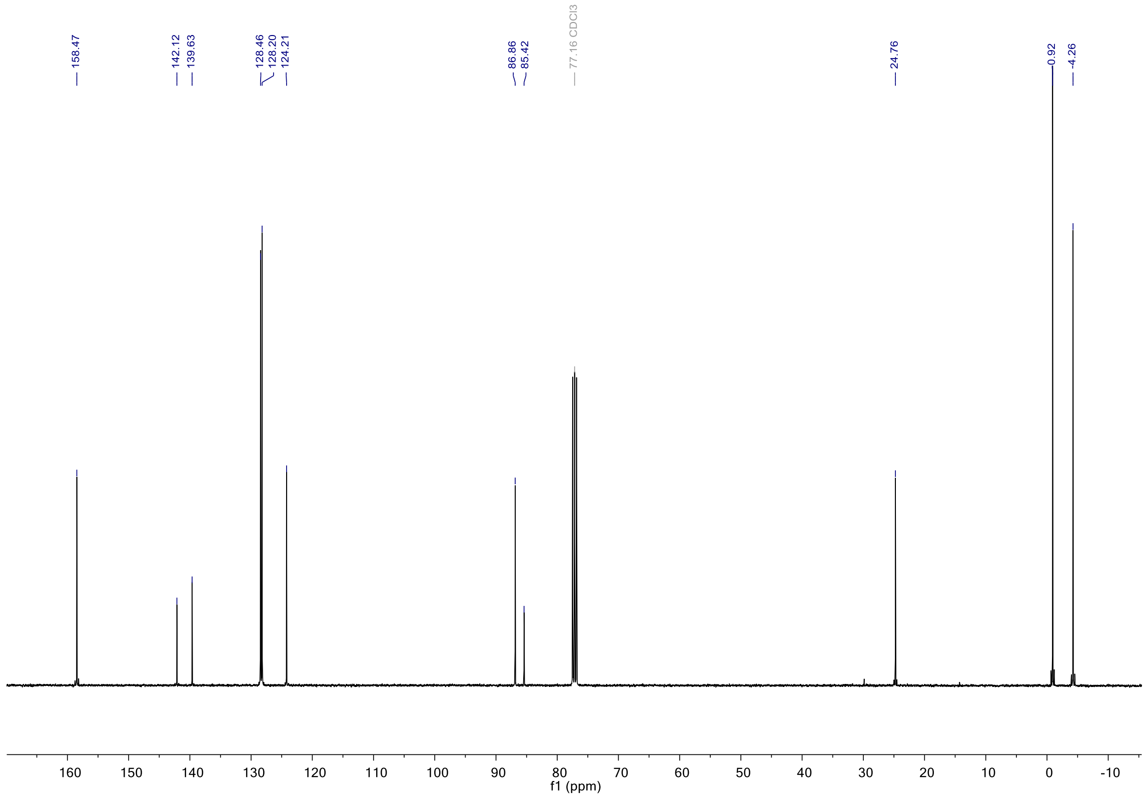

Figure S33. ^{13}C NMR spectrum of **5**.

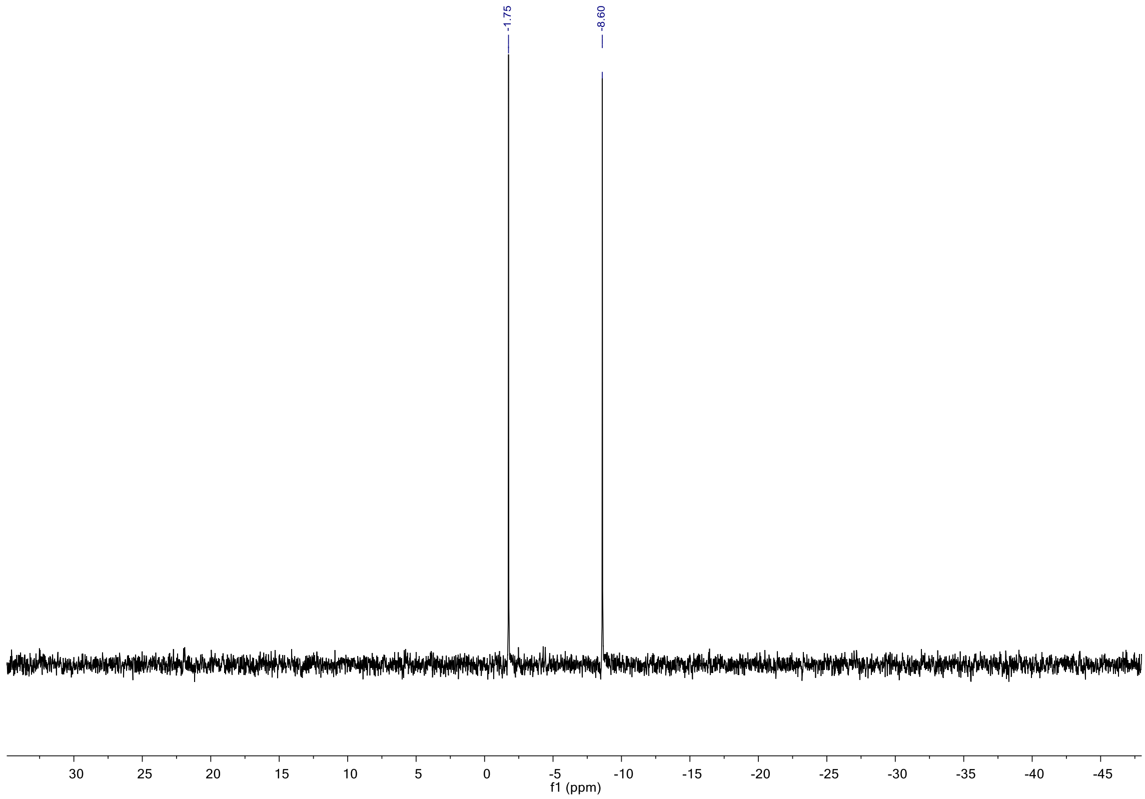

Figure S34. ^{29}Si NMR spectrum of **5**.

(*E*)-Benzyldimethyl(4-phenyl-1-(trimethylsilyl)but-1-en-3-yn-2-yl)silane (6) – New compound

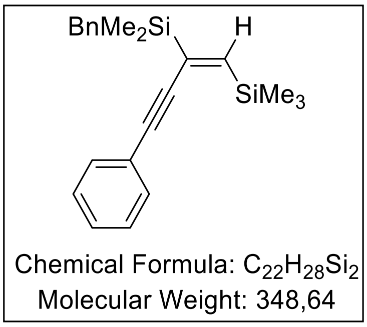

^{1}H NMR ($CDCl_3$, 400 MHz, δ, ppm): 7.39-6.98 (m, 10H, Ph), 6.46 (s, 1H, =C<u>H</u>), 2.24 (s, 2H, C<u>H</u>$_2$Ph), 0.19 (s, 9H, SiC<u>H</u>$_3$), 0.15 (s, 6H, SiC<u>H</u>$_3$). **^{13}C NMR** ($CDCl_3$, 101 MHz, δ, ppm): 156.0 (=<u>C</u>H), 142.7 (<u>C</u>=CH), 139.7, 131.3, 128.5, 128.2, 128.2, 128.1, 124.4, 124.2 (Ph), 99.1 (≡<u>C</u>Ph), 91.6 (<u>C</u>≡CPh), 25.1 (<u>C</u>H$_2$Ph), -0.7, -4.0 (Si<u>C</u>H$_3$). **^{29}Si NMR** ($CDCl_3$, 79 MHz, δ, ppm): -1.99 (<u>Si</u>Me$_2$Bn), -8.69 (<u>Si</u>Me$_3$). **GC-MS** (EI, 70 eV) m/z (rel. int., %): 348.2 (M^+, 3.6), 258.2 (15.5), 257.1 (59.9), 242.1 (12.5), 241.1 (49.2), 183.0 (11.6), 159.0 (26.1), 156.1 (17.2), 155.0 (100.0), 121.0 (27.5), 97.0 (23.6), 91.0 (57.7), 73.0 (91.4). **FT-IR** (cm^{-1}): 2954, 2895, 1598, 1492, 1451, 1407, 1245, 1205, 1153, 1056, 1029, 827, 791, 752, 688, 624, 522, 475. **Elem. Anal.** calcd for $C_{22}H_{28}Si_2$: C, 75.79; H, 8.10; found C, 75.63; H, 8.05. Pale yellow oil. Isolated yield = 80% (232.3 mg).

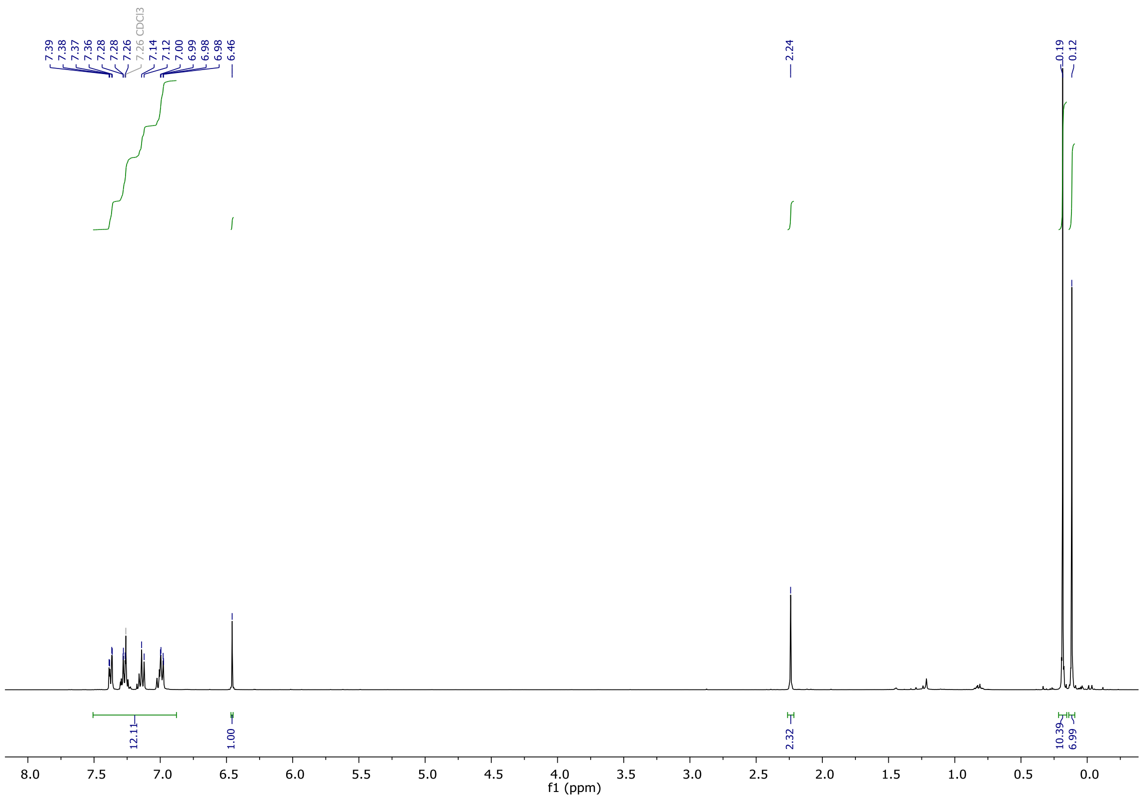

Figure S35. ^{1}H NMR spectrum of **6**.

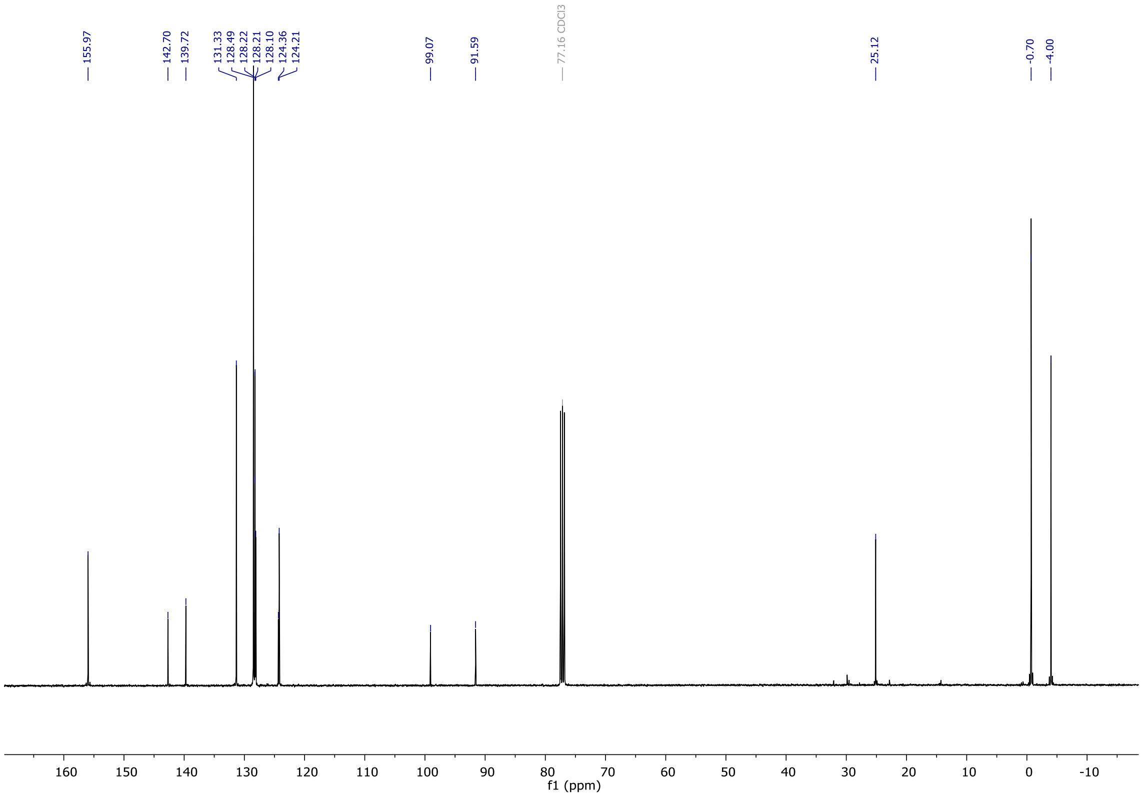

Figure S36. ^{13}C NMR spectrum of **6**.

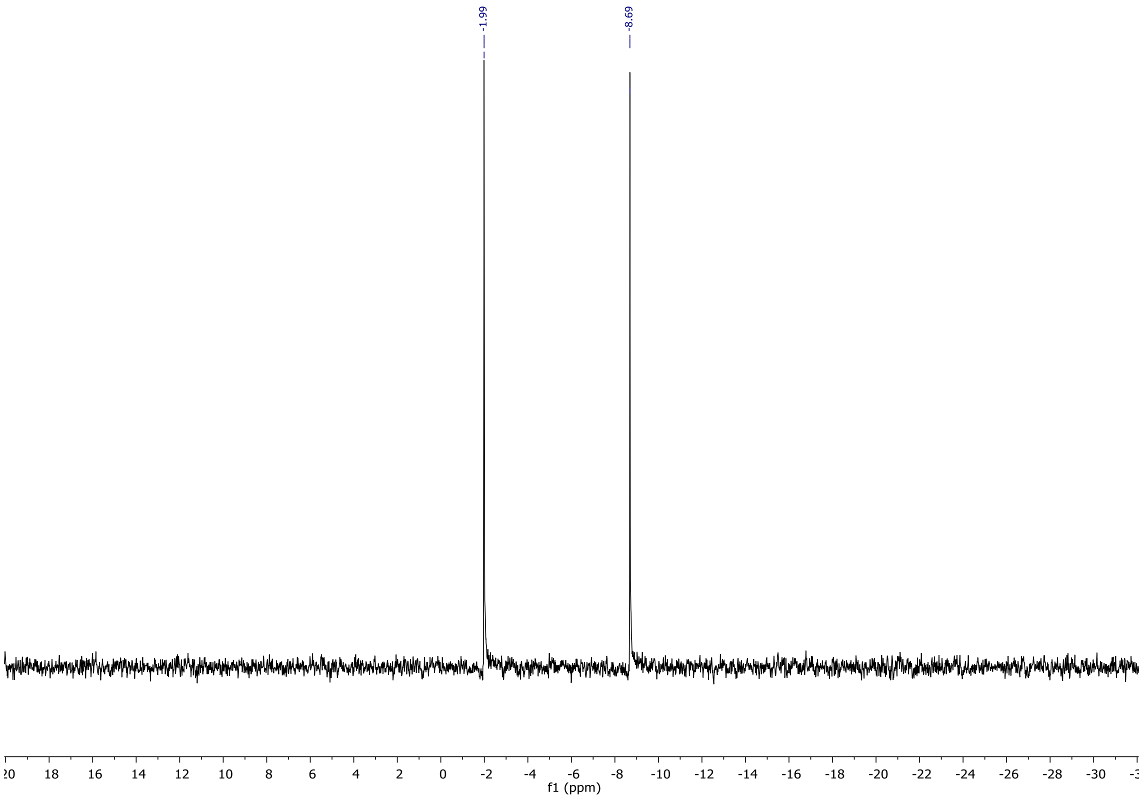

Figure S37. ^{29}Si NMR spectrum of **6**.

3-((1*E*,3*E*)-3-(Benzyldimethylsilyl)-1-phenyl-4-(trimethylsilyl)buta-1,3-dien-2-yl)-1,1,1,5,5,5-hexamethyl-3-((trimethylsilyl)oxy)trisiloxane (7) – New compound

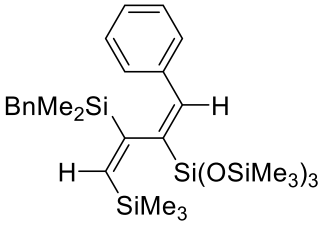

Chemical Formula: $C_{31}H_{56}O_3Si_6$
Molecular Weight: 645,30

^{1}H NMR ($CDCl_3$, 400 MHz, δ, ppm): 7.57-7.04 (m, 10H, Ph), 6.72 (s, 1H, =C<u>H</u>), 6.25 (s, 1H, =C<u>H</u>), 2.27 (s, 2H, C<u>H2</u>Ph), 0.28 (s, 27H, OSiC<u>H3</u>), 0.09 (s, 9H, SiC<u>H3</u>), 0.08 (s, 3H, SiC<u>H3</u>), 0.03 (s, 3H, SiC<u>H3</u>). **^{13}C NMR** ($CDCl_3$, 101 MHz, δ, ppm): 164.2, 145.6, 141.9, 140.5 (<u>C</u>=<u>C</u>), 138.4, 135.6, 128.9, 128.2, 128.0, 127.3, 124.0 (Ph), 26.2 (<u>C</u>H2Ph), 2.4 (OSi<u>C</u>H3), 0.0 ((Si<u>C</u>H3)3), -2.2, -2.8 ((Si<u>C</u>H3)2). **^{29}Si NMR** ($CDCl_3$, 79 MHz, δ, ppm): 8.03 (Si(O<u>Si</u>Me3)3), -5.13 (<u>Si</u>Me2Bn), -9.45 (<u>Si</u>Me3), -82.28 (<u>Si</u>(OSiMe3)3). **GC-MS** (EI, 70 eV) m/z (rel. int., %): 644.0 (M^+, 0.2), 554.2 (11.8), 533.1 (23.1), 390.9 (10.2), 302.9 (11.3), 206.9 (36.6), 148.8 (28.6), 146.9 (17.0), 120.8 (27.5), 72.9 (100.0). **FT-IR** (cm^{-1}): 3024, 2956, 2898, 1600, 14922, 1446, 1249, 1204, 1040, 832, 752, 691, 628, 588, 548, 529, 425. **Elem. Anal.** calcd for $C_{31}H_{56}O_3Si_6$: C, 57.70; H, 8.75; found C, 57.82; H, 8.80. Pale yellow oil. Isolated yield = 82% (352.1 mg).

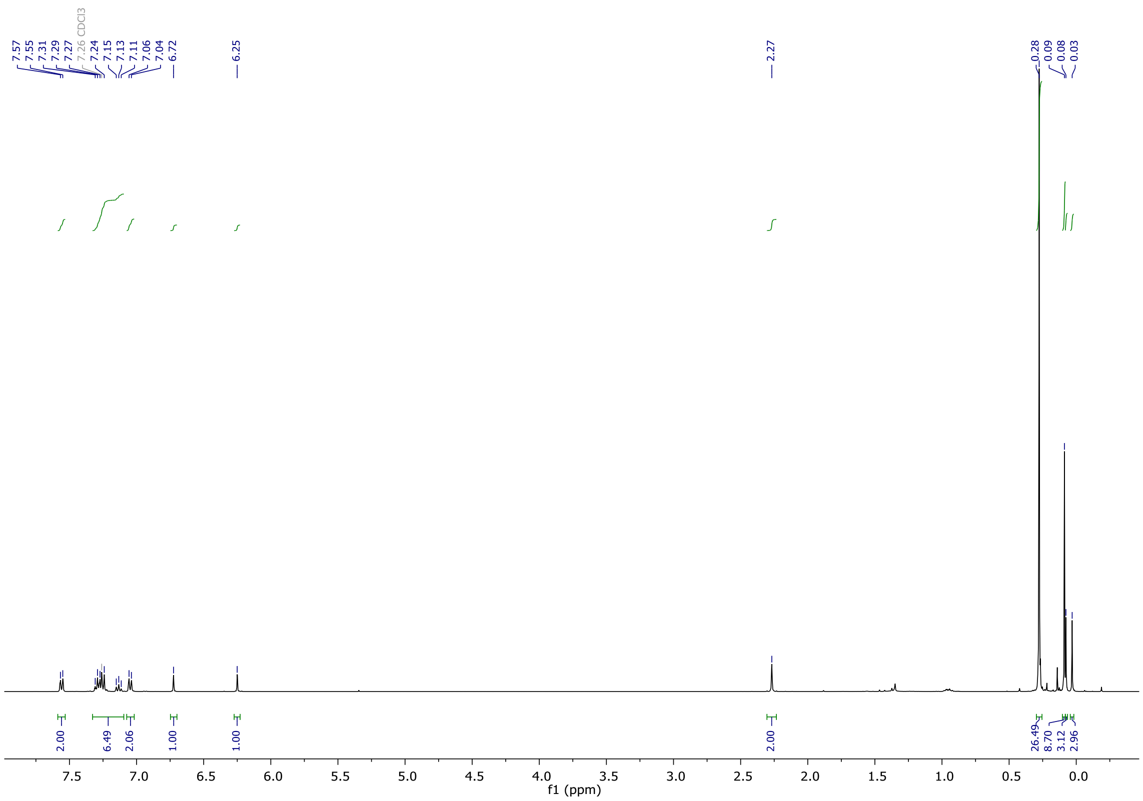

Figure S38. ^{1}H NMR spectrum of **7**.

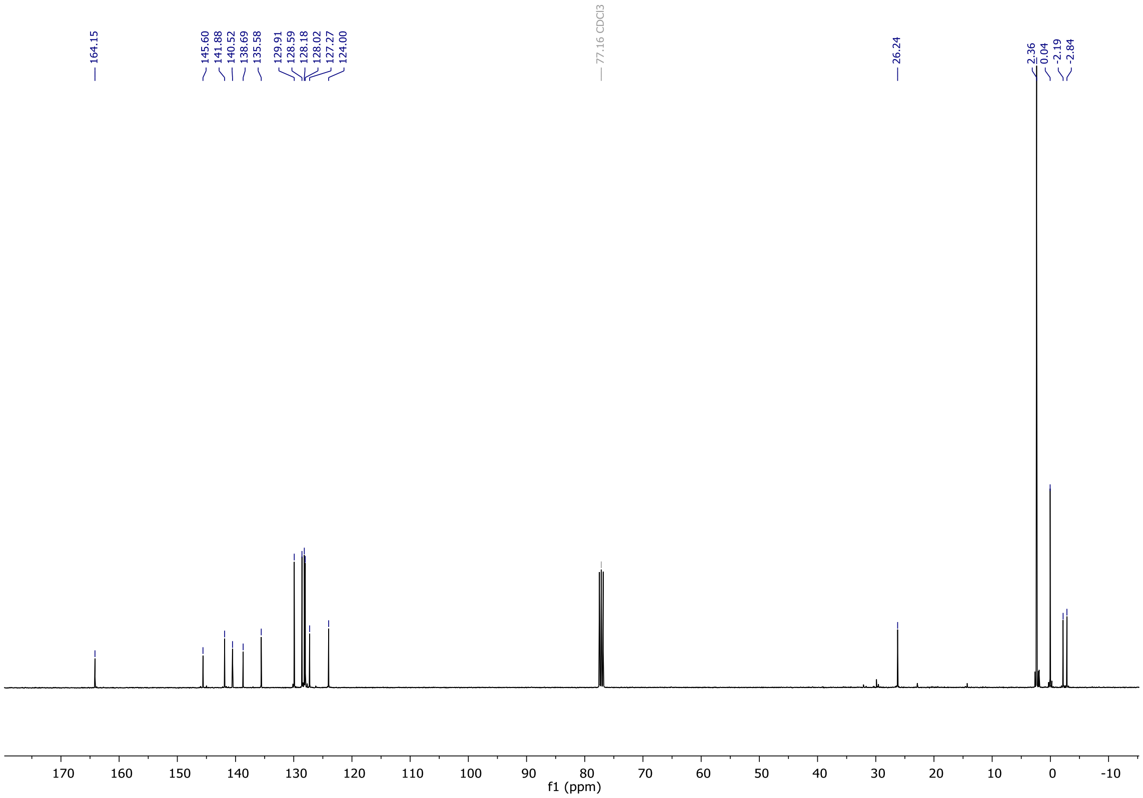

Figure S39. ^{13}C NMR spectrum of **7**.

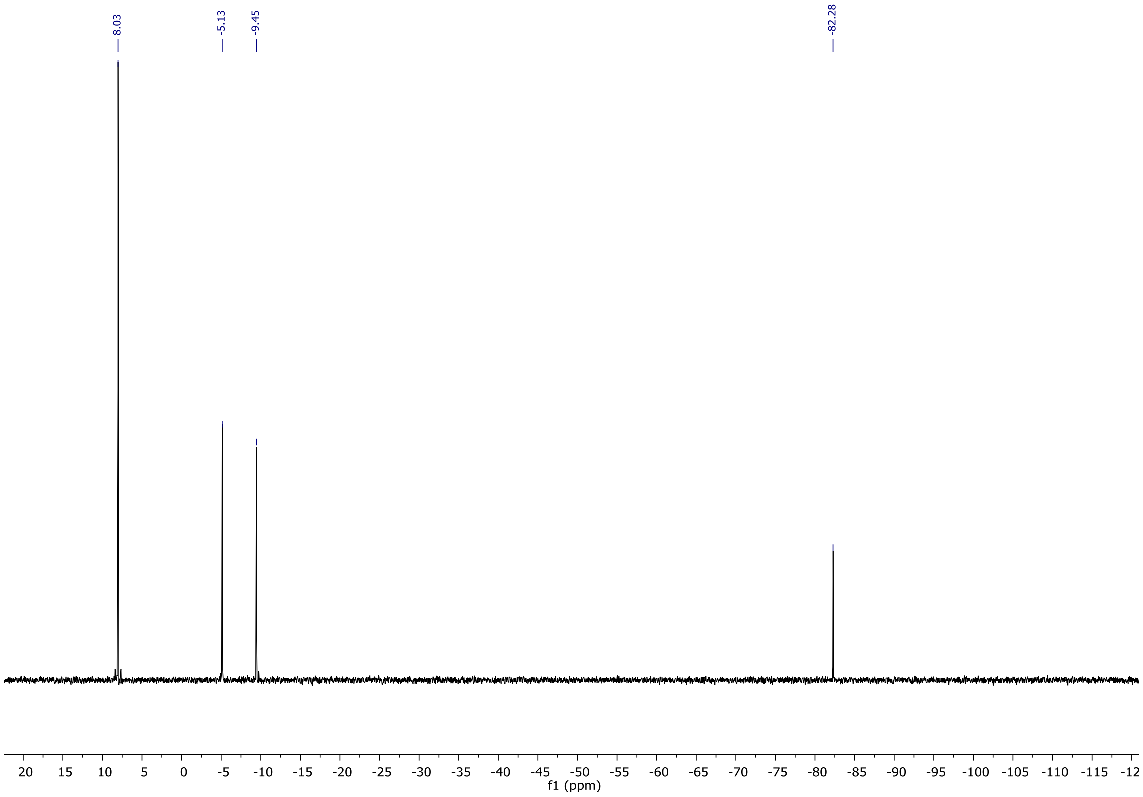

Figure S40. ^{29}Si NMR spectrum of **7**.

((1*E*,3*E*)-4-(Trimethylsilyl)buta-1,3-diene-1,3-diyl)bis(benzyldimethylsilane) (8) – New compound

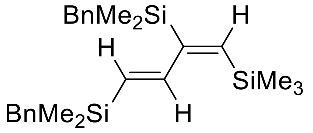

Chemical Formula: $C_{25}H_{38}Si_3$
Molecular Weight: 422,83

[1]**H NMR** ($CDCl_3$, 300 MHz, δ, ppm): 7.34-7.04 (m, 10H, Ph), 6.92 (dd, 1H, $J_{(H,H)}$= 19.36, 1.12 Hz, =C<u>H</u>), 6.20 (s, 1H, =C<u>H</u>), 5.81 (d, 1H, $J_{(H,H)}$= 19.38 Hz, =C<u>H</u>), 2.27 (s, 2H, C<u>H</u>$_2$Ph), 2.24 (s, 2H, C<u>H</u>$_2$Ph), 0.18 (s, 9H, SiC<u>H</u>$_3$), 0.17 (s, 6H, SiC<u>H</u>$_3$), 0.16 (s, 6H, SiC<u>H</u>$_3$). [13]**C NMR** ($CDCl_3$, 101 MHz, δ, ppm): 160.7 (<u>C</u>=<u>C</u>), 149.0, 148.2 (Ph), 140.2, 140.0, 130.9 (<u>C</u>=<u>C</u>), 128.4, 128.4, 128.3, 128.2, 128.1, 124.2, 124.1 (Ph), 26.3, 26.3 (<u>C</u>H_2Ph), 0.5 (Si(<u>C</u>H_3)$_3$), -2.4, -3.2 (Si(<u>C</u>H_3)$_2$). [29]**Si NMR** ($CDCl_3$, 79 MHz, δ, ppm): -4.63, -6.06 (<u>Si</u>Me_2Bn), -10.88 (<u>Si</u>Me_3). **GC-MS** (EI, 70 eV) m/z (rel. int., %): 422.0 (M^+, 0.4), 332.1 (10.1), 331.0 (29.2), 243.0 (19.5), 148.9 (100.0), 166.9 (45.0), 150.1 (13.9), 120.8 (59.5), 90.9 (11.9), 82.8 (10.7), 72.9 (83.8). **FT-IR** (cm^{-1}): 3060, 3024, 2954, 2894, 1600, 1492, 1451, 1406, 1246, 1205, 1153, 1055, 987, 902, 825, 758, 695, 621, 556, 475. **Elem. Anal.** calcd for $C_{25}H_{38}Si_3$: C, 71.01; H, 9.06; found C, 71.12; H, 9.11. Pale yellow oil. Isolated yield = 88% (136.5 mg).

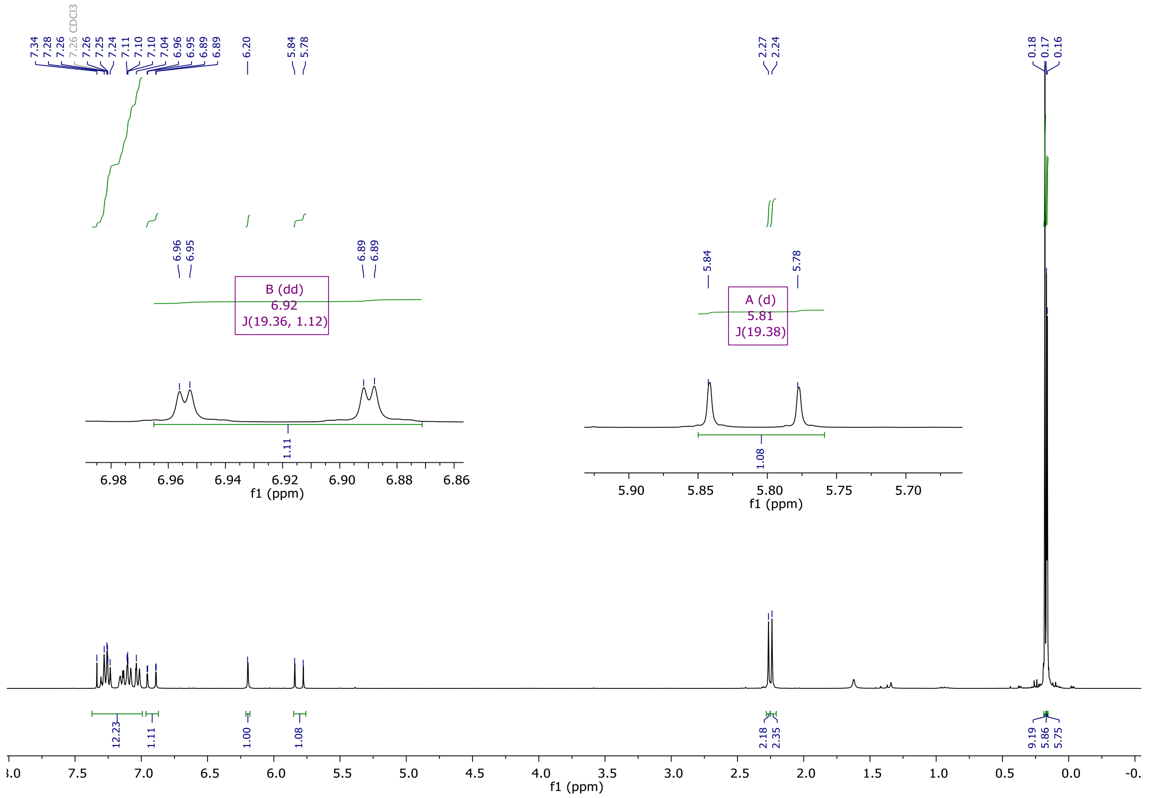

Figure S41. ^{1}H NMR spectrum of **8**.

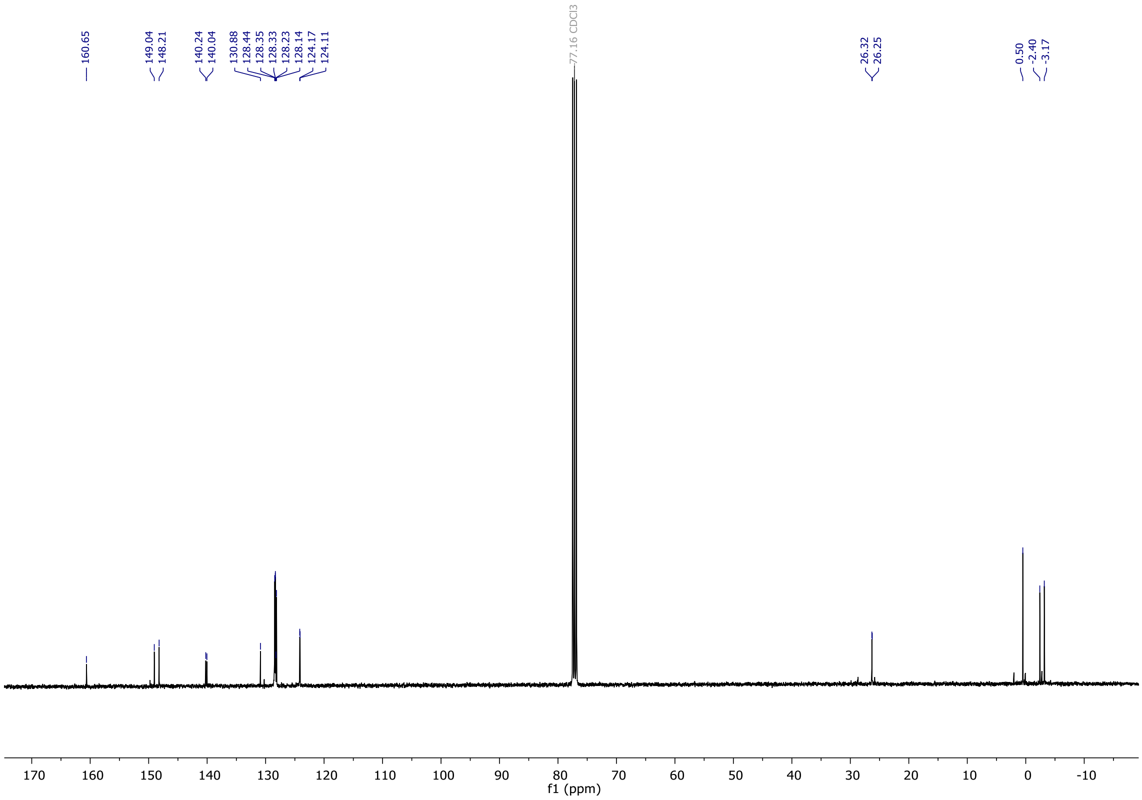

Figure S42. ^{13}C NMR spectrum of **8**.

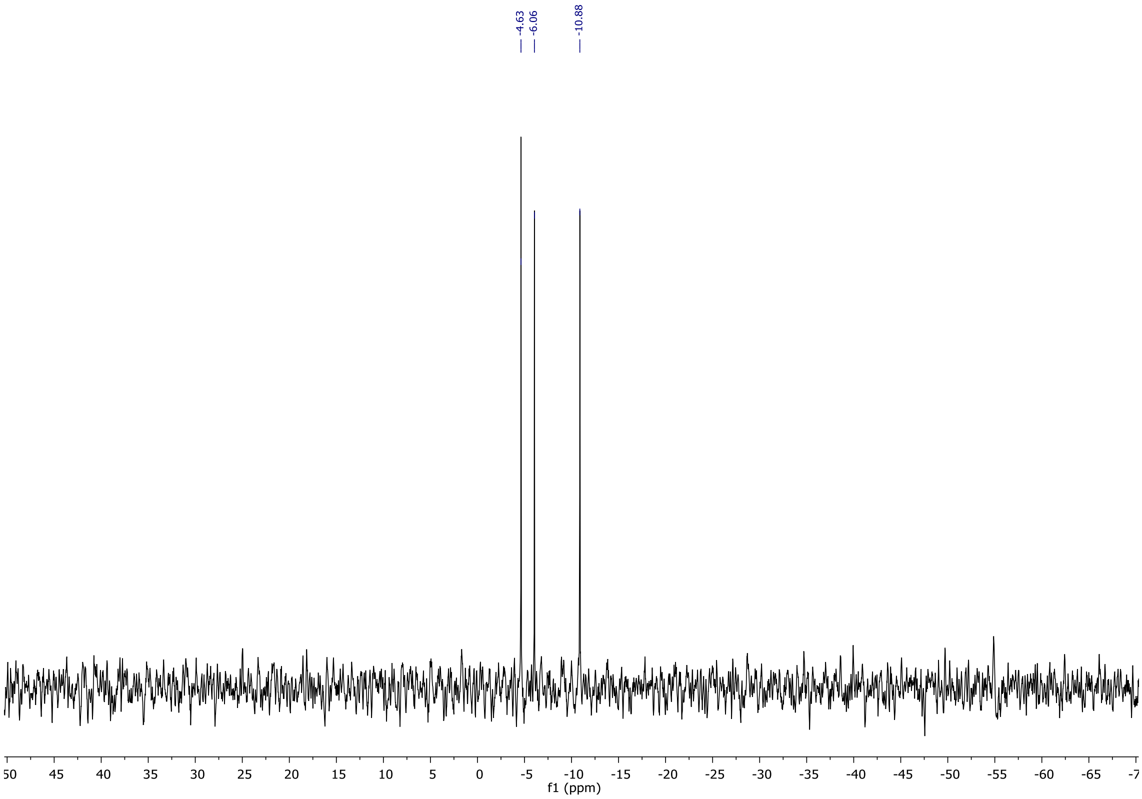

Figure S43. ^{29}Si NMR spectrum of **8**.

(*E*)-Benzyl(1-iodo-4-(trimethylsilyl)but-1-en-3-yn-2-yl)dimethylsilane (9a) – New compound

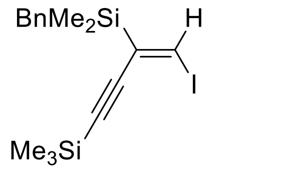

Chemical Formula: $C_{16}H_{23}ISi_2$
Molecular Weight: 398,43

^{1}**H NMR** ($CDCl_3$, 300 MHz, δ, ppm): 7.26-7.03 (m, 5H, Ph), 6.87 (s, 1H, =CH), 2.30 (s, 2H, CH_2Ph), 0.20 (s, 9H, $SiCH_3$), 0.19 (s, 6H, $SiCH_3$). ^{13}**C NMR** ($CDCl_3$, 101 MHz, δ, ppm): 140.5 (Ph), 138.7 (C=C), 128.4, 128.4, 124.6 (Ph), 107.4 (≡CSiMe$_3$), 106.7 (=CH), 97.9 (C≡CSiMe$_3$), 25.0 (CH_2Ph), 0.1, -3.8 ($SiCH_3$). ^{29}**Si NMR** ($CDCl_3$, 79 MHz, δ, ppm): 0.21 ($SiMe_2$Bn), -17.01 ($SiMe_3$). **GC-MS** (EI, 70 eV) m/z (rel. int., %): 271.0 (M^+ - 127.4, 32.9), 184.7 (25.2), 182.9 (18.9), 164.9 (9.4), 150.1 (10.5), 148.9 (72.1), 120.9 (43.3), 106.8 (40.6), 90.9 (21.0), 72.9 (100.0). **FT-IR** (cm^{-1}): 3024, 2957, 2897, 2140, 1599, 1519, 1492, 1451, 1407, 1248, 1205, 1155, 1056, 836, 757, 697, 627, 603, 498, 474. **Elem. Anal.** calcd for $C_{16}H_{23}ISi_2$: C, 48.23; H, 5.82; found C, 48.32; H, 5.87. Pale yellow oil. Isolated yield = 89% (308.6 mg).

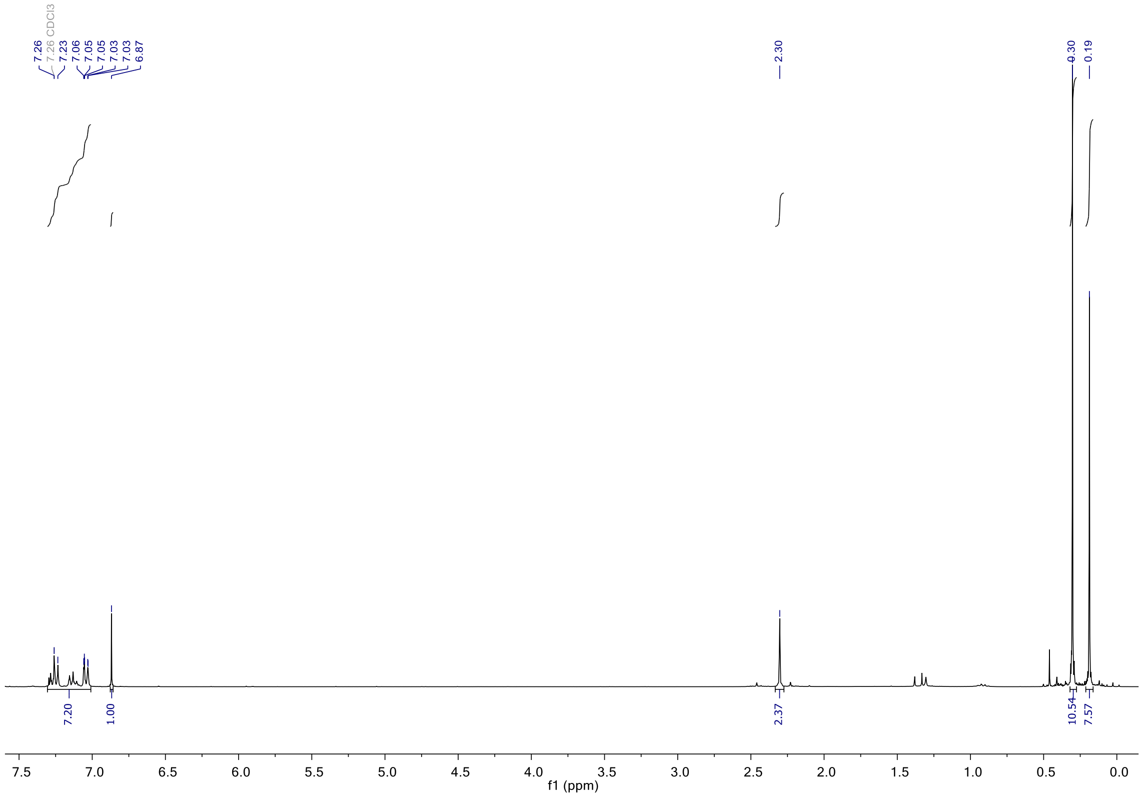

Figure S44. ^{1}H NMR spectrum of **9a**.

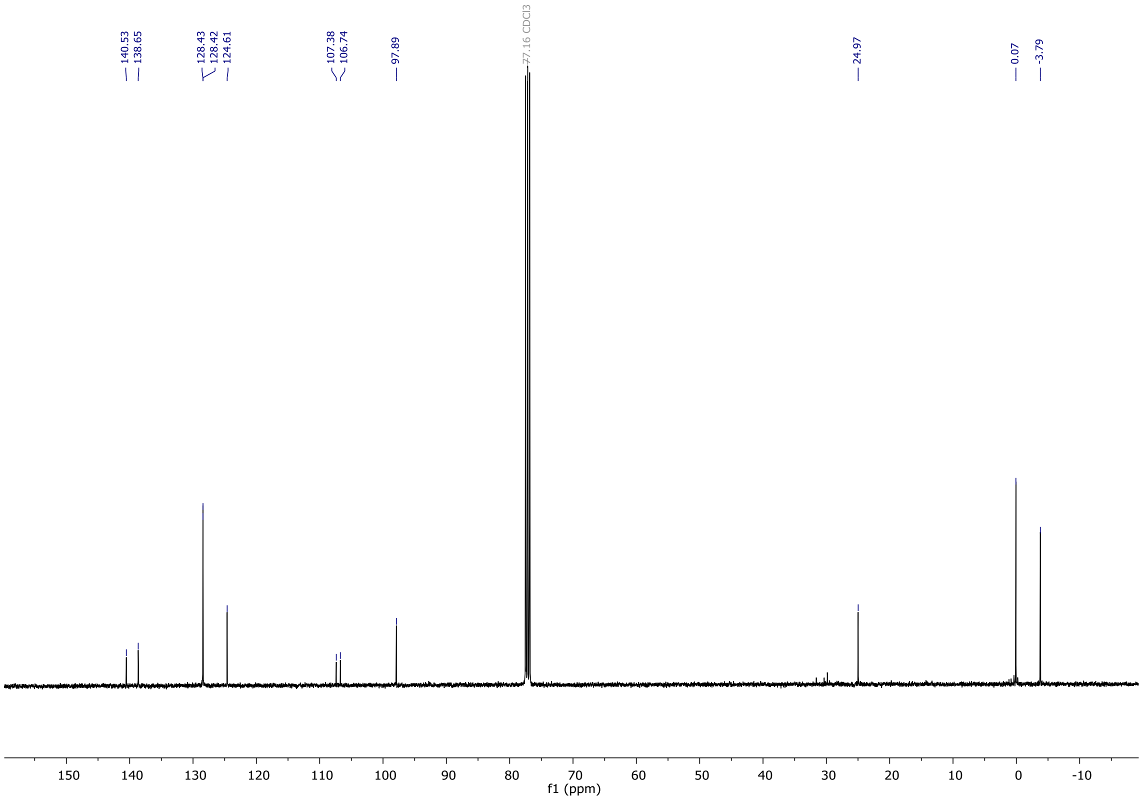

Figure S45. ^{13}C NMR spectrum of **9a**.

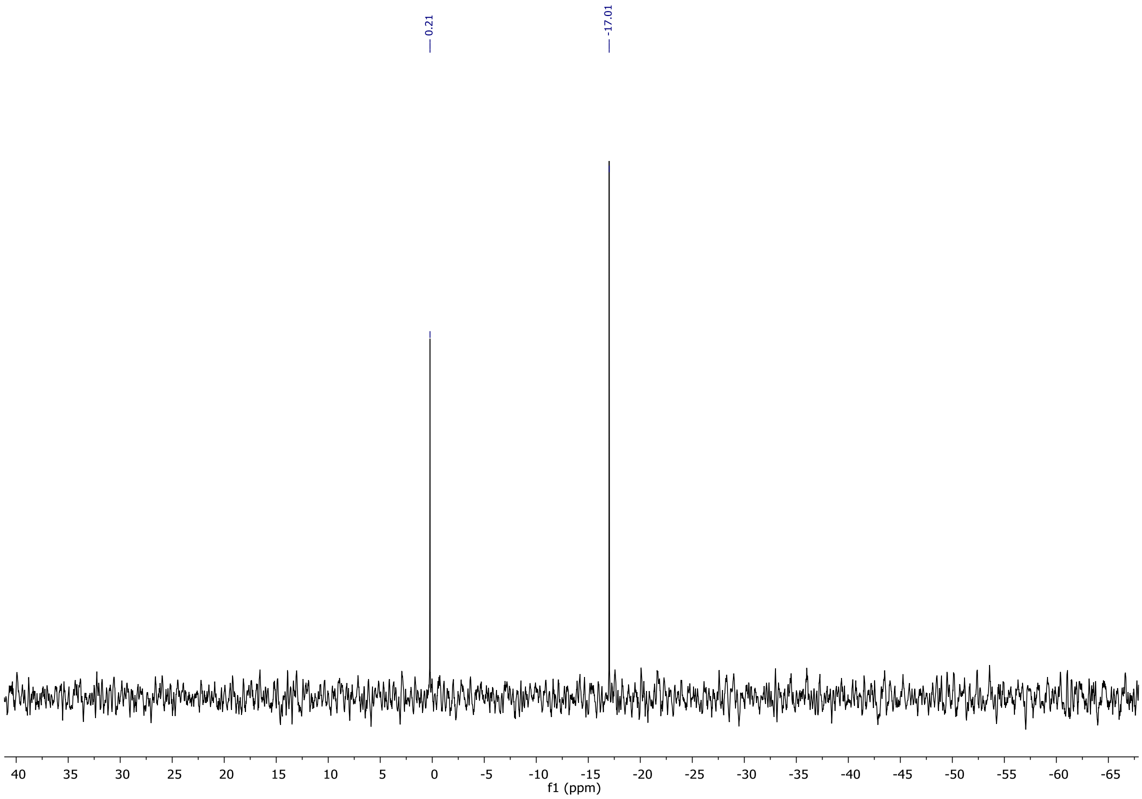

Figure S46. ^{29}Si NMR spectrum of **9a**.

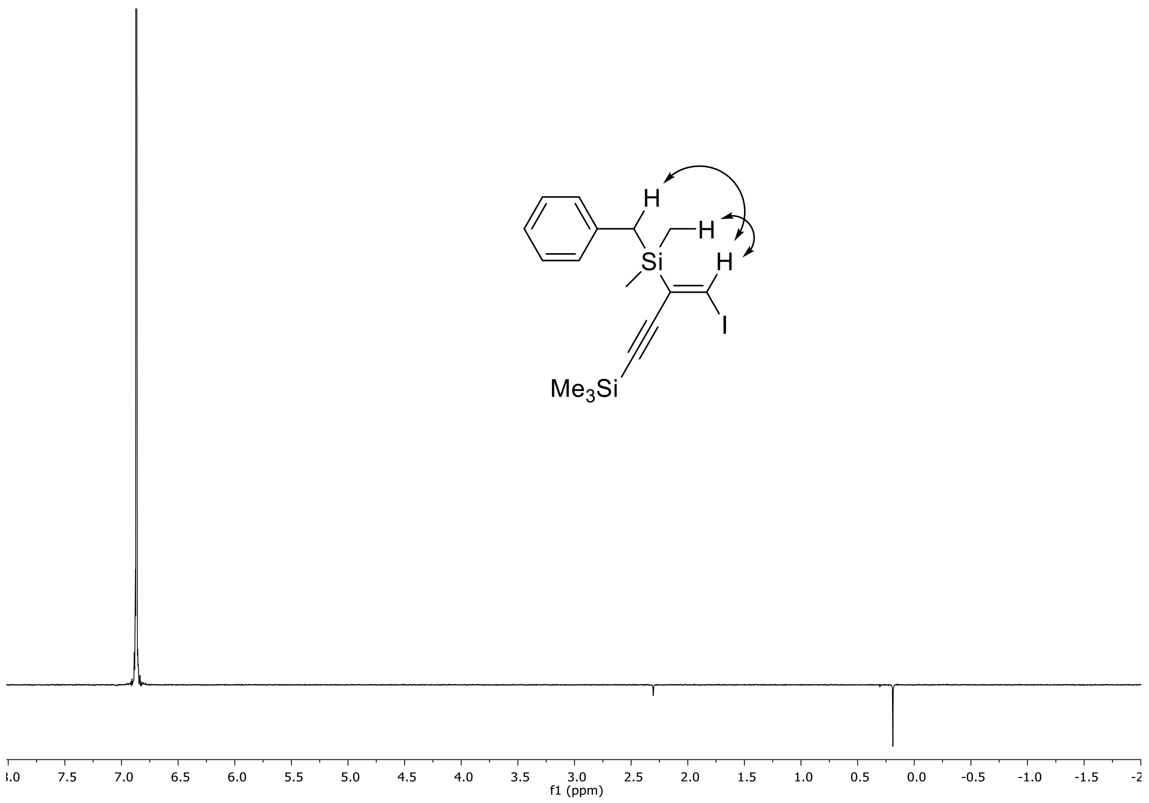

Figure S47. 1D selective gradient NOE NMR of compound **9a** directed at alkenyl proton at 6.87 ppm.

(*E*)-Benzyl(1-bromo-4-(trimethylsilyl)but-1-en-3-yn-2-yl)dimethylsilane (9b) – New compound

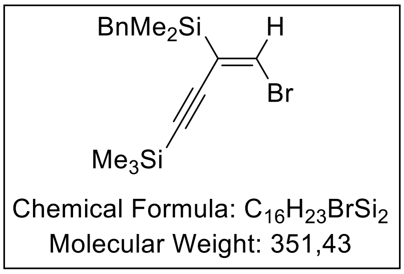

^{1}**H NMR** ($CDCl_3$, 300 MHz, δ, ppm): 7.30-7.04 (m, 5H, Ph), 6.54 (s, 1H, $=C\underline{H}$), 2.31 (s, 2H, $C\underline{H_2}Ph$), 0.29 (s, 9H, $SiC\underline{H_3}$), 0.19 (s, 6H, $SiC\underline{H_3}$). ^{13}**C NMR** ($CDCl_3$, 101 MHz, δ, ppm): 138.6 (Ph), 131.5 ($\underline{C}$=C), 128.5, 128.4, 124.6 (Ph), 121.1 (=$\underline{C}$H), 107.5 (≡$\underline{C}SiMe_3$), 102.8 ($\underline{C}$≡$CSiMe_3$), 24.8 ($\underline{C}H_2Ph$), 0.1, -4.0 ($Si\underline{C}H_3$). ^{29}**Si NMR** ($CDCl_3$, 79 MHz, δ, ppm): 0.05 ($\underline{Si}Me_2Bn$), -17.26 ($\underline{Si}Me_3$). **GC-MS** (EI, 70 eV) m/z (rel. int., %): 272.1 (M^+ - 79.33, 7.0), 270.9 (25.3), 148.9 (16.3), 138.7 (17.8), 136.7 (18.0), 120.8 (24.5), 106.8 (53.0), 90.9 (18.5), 72.9 (100.0). **FT-IR** (cm^{-1}): 3025, 2959, 2897, 1600, 1534, 1493, 1452, 1407, 1249, 1206, 1156, 1067, 836, 758, 697, 674, 631, 499, 475. **Elem. Anal.** calcd for $C_{16}H_{23}BrSi_2$: C, 54.58; H, 6.60; found C, 54.51; H, 6.53. Pale yellow oil. Isolated yield = 82% (250.8 mg).

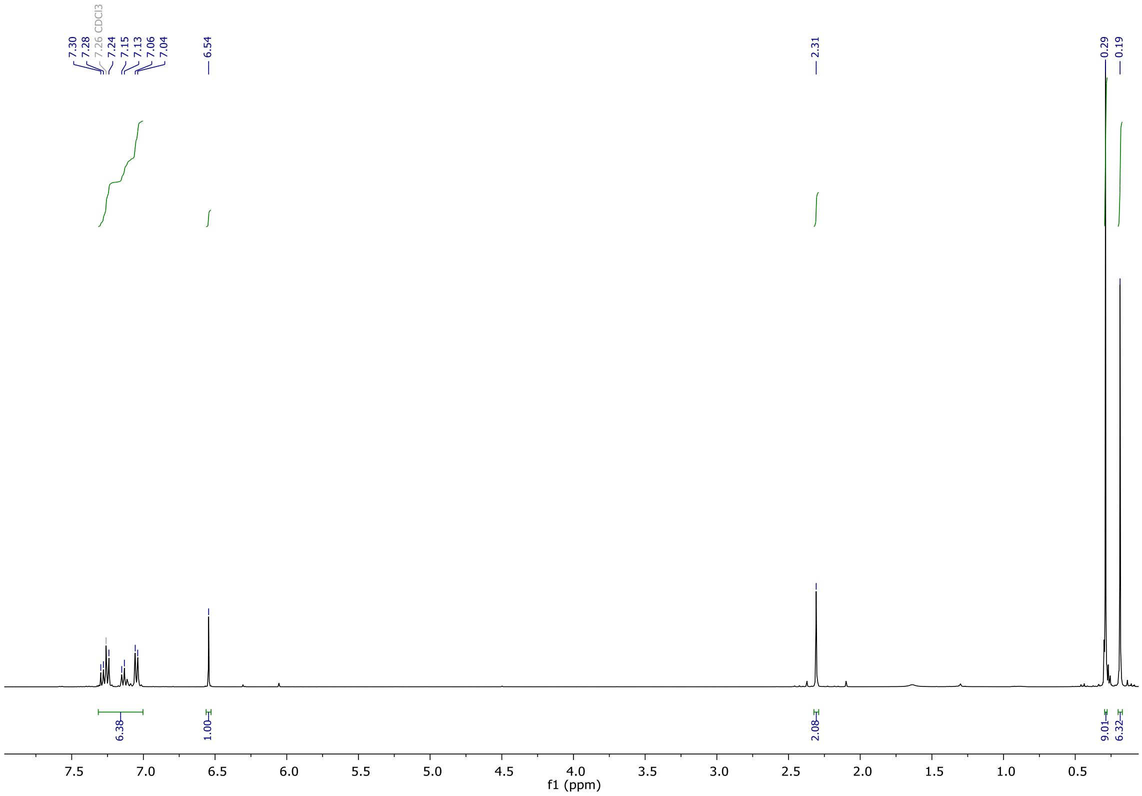

Figure S48. ^{1}H NMR spectrum of **9b**.

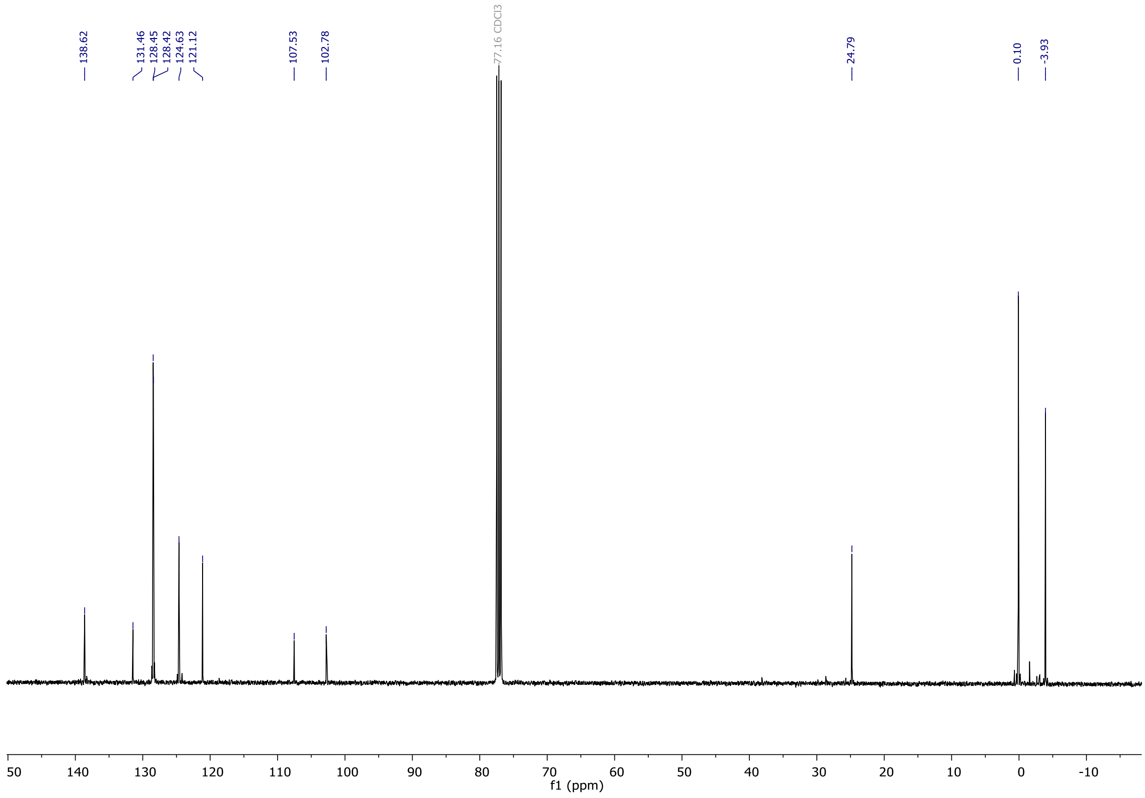

Figure S49. ^{13}C NMR spectrum of **9b**.

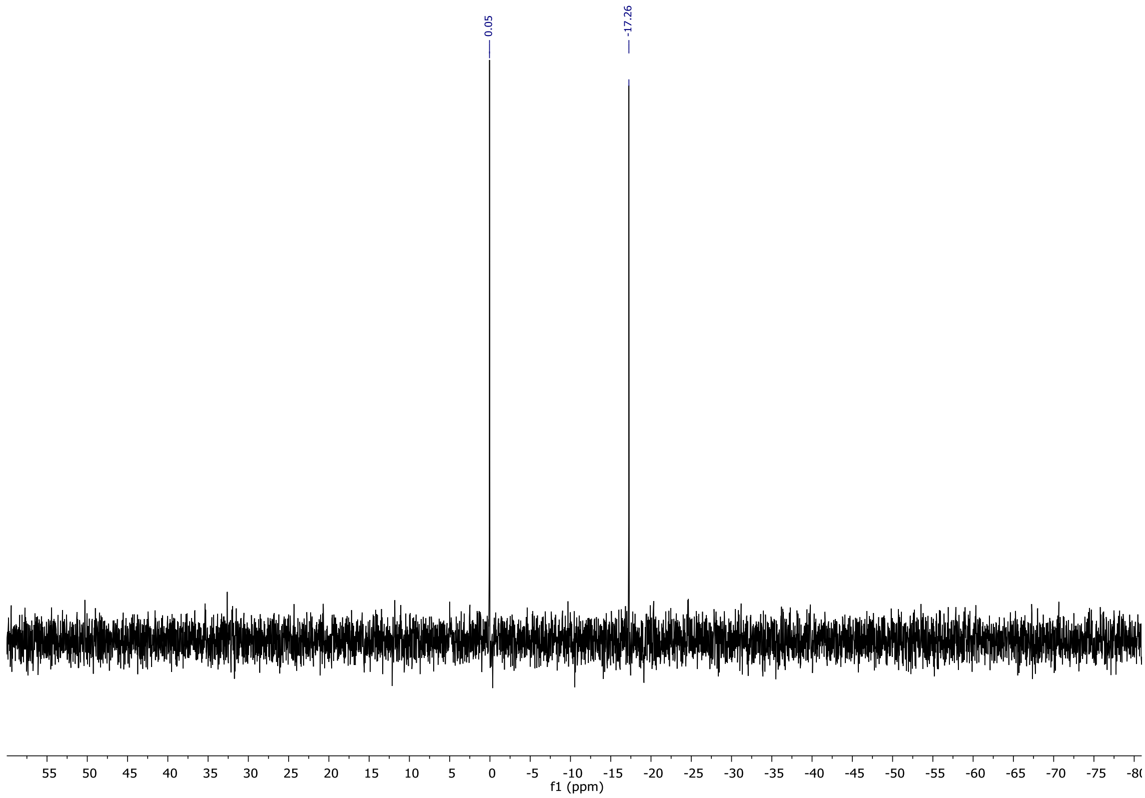

Figure S50. ^{29}Si NMR spectrum of **9b**.

(*E*)-benzyldimethyl(1-phenyl-4-(trimethylsilyl)but-1-en-3-yn-2-yl)silane (10) – New compound

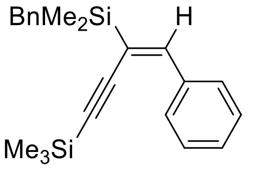

Chemical Formula: $C_{22}H_{28}Si_2$
Molecular Weight: 348,64

[1]**H NMR** ($CDCl_3$, 300 MHz, δ, ppm): 7.87-6.93 (m, 10H, Ph), 6.60 (s, 1H, =C$\underline{H}$), 2.22 (s, 2H, C$\underline{H_2}$Ph), 0.15 (s, 9H, SiC$\underline{H_3}$), 0.08 (s, 6H, SiC$\underline{H_3}$). [13]**C NMR** ($CDCl_3$, 151 MHz, δ, ppm): 146.2 (=$\underline{C}$H), 139.6 ($\underline{C}$=CH), 137.6, 129.0, 128.9, 128.5, 128.3, 128.3, 124.3, 122.3 (Ph), 107.6 (≡$\underline{C}$SiMe$_3$), 106.0 ($\underline{C}$≡CSiMe$_3$), 24.9 ($\underline{C}$H$_2$Ph), 0.1, -4.0 (Si$\underline{C}$H$_3$). [29]**Si NMR** ($CDCl_3$, 191 MHz, δ, ppm): -0.40 ($\underline{Si}$Me$_2$Bn), -18.31 ($\underline{Si}$Me$_3$). **GC-MS** (EI, 70 eV) m/z (rel. int., %): 348.2 (M^+, 3.6), 258.2 (15.5), 257.1 (59.9), 242.1 (12.5), 241.1 (49.2), 183.0 (11.6), 159.0 (26.1), 156.1 (17.2), 155.0 (100.0), 121.0 (27.5), 97.0 (23.6), 91.0 (57.7), 73.0 (91.4). **FT-IR** (cm^{-1}): 3024, 2958, 2896, 1600, 1493, 1447, 1248, 1206, 1059, 860, 834, 754, 689, 634, 515. **Elem. Anal.** calcd for $C_{22}H_{28}Si_2$: C, 75.79; H, 8.10; found C, 75.67; H, 8.05. Pale yellow oil. Isolated yield = 76% (205 mg).

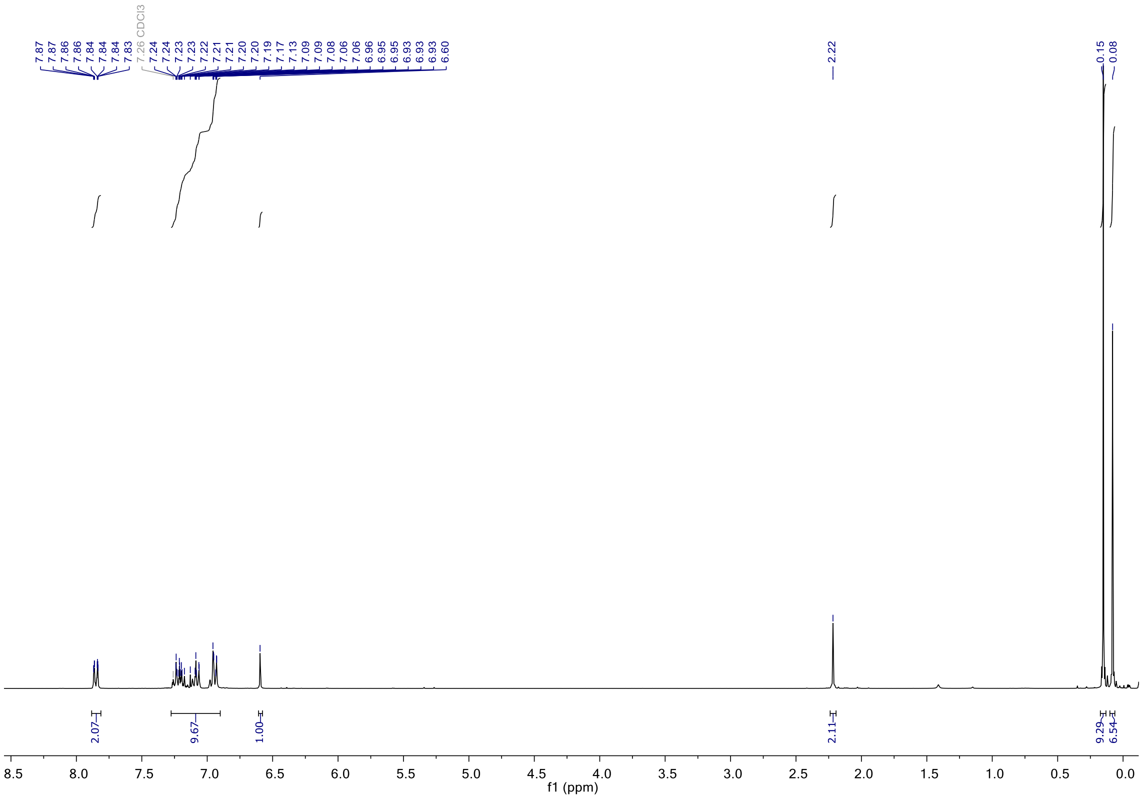

Figure S51. ^{1}H NMR spectrum of **10**.

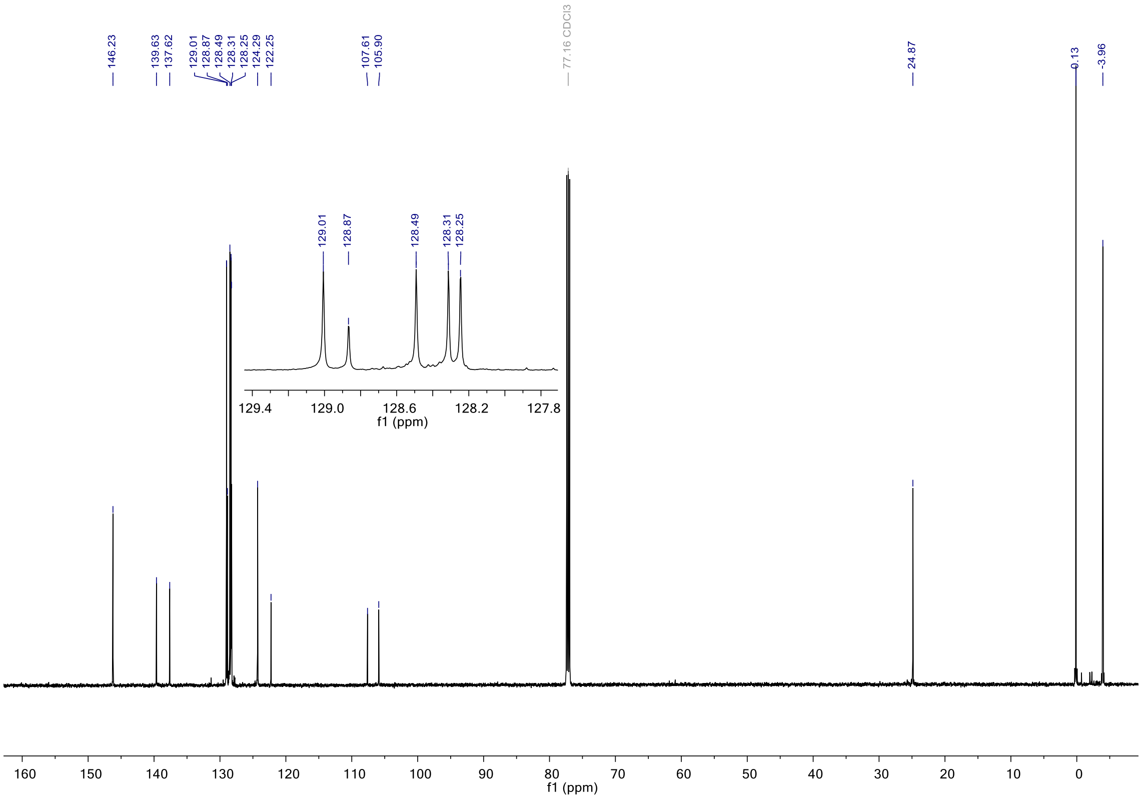

Figure S52. ^{13}C NMR spectrum of **10**.

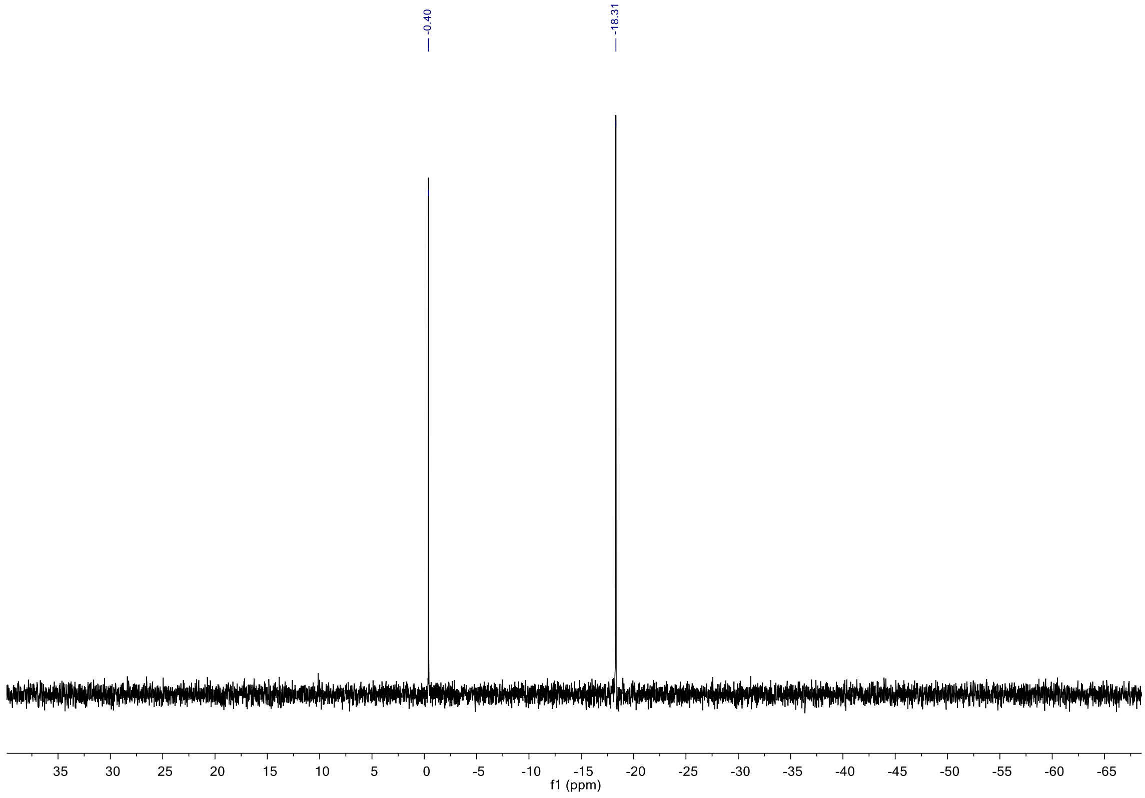

Figure S53. ^{29}Si NMR spectrum of **10**.

(*E*)-Benzyldimethyl(1-phenylbut-1-en-3-yn-2-yl)silane (11) – New compound

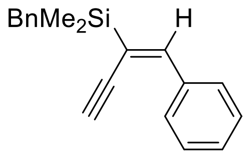

Chemical Formula: $C_{19}H_{20}Si$
Molecular Weight: 276,45

^{1}H NMR ($CDCl_3$, 400 MHz, δ, ppm): 7.88-6.99 (m, 10H, Ph), 6.73 (s, 1H, =C$\underline{H}$), 3.65 (d, 1H, $J_{(H,H)}$= 1.07 Hz, ≡C$\underline{H}$), 2.29 (s, 2H, C$\underline{H_2}$Ph), 0.16 (s, 6H, SiC$\underline{H_3}$). **^{13}C NMR** ($CDCl_3$, 101 MHz, δ, ppm): 147.5 (=$\underline{C}$H), 139.5 ($\underline{C}$=CH), 137.4, 129.0, 128.9, 128.5, 128.4, 128.3, 124.3, 121.2 (Ph), 89.3 (≡$\underline{C}$H), 84.1 ($\underline{C}$≡CH), 24.7 ($\underline{C}H_2$Ph), -4.0 (Si$\underline{C}H_3$). **^{29}Si NMR** ($CDCl_3$, 191 MHz, δ, ppm): 0.16 ($\underline{Si}Me_2$Bn). **GC-MS** (EI, 70 eV) m/z (rel. int., %): 276.0 (M^+, 6.5), 186.0 (17.3), 185.0 (100.0), 184.0 (19.6), 183.0 (94.3), 168.9 (40.4), 158.9 (14.7), 120.9 (11.8), 90.9 (21.9), 82.9 (65.0), 65.0 (11.8), 59.0 (11.7). **FT-IR** (cm^{-1}): 3303, 3060, 3024, 2956, 2924, 2853, 1599, 1562, 1493, 1447, 1407, 1248, 1206, 1155, 1057, 1022, 830, 792, 755, 689, 616, 594, 475, 458. **Elem. Anal.** calcd for $C_{19}H_{20}Si_2$: C, 82.55; H, 7.29; found C, 82.59; H, 7.32. Pale yellow oil. Isolated yield = 91% (147.9 mg).

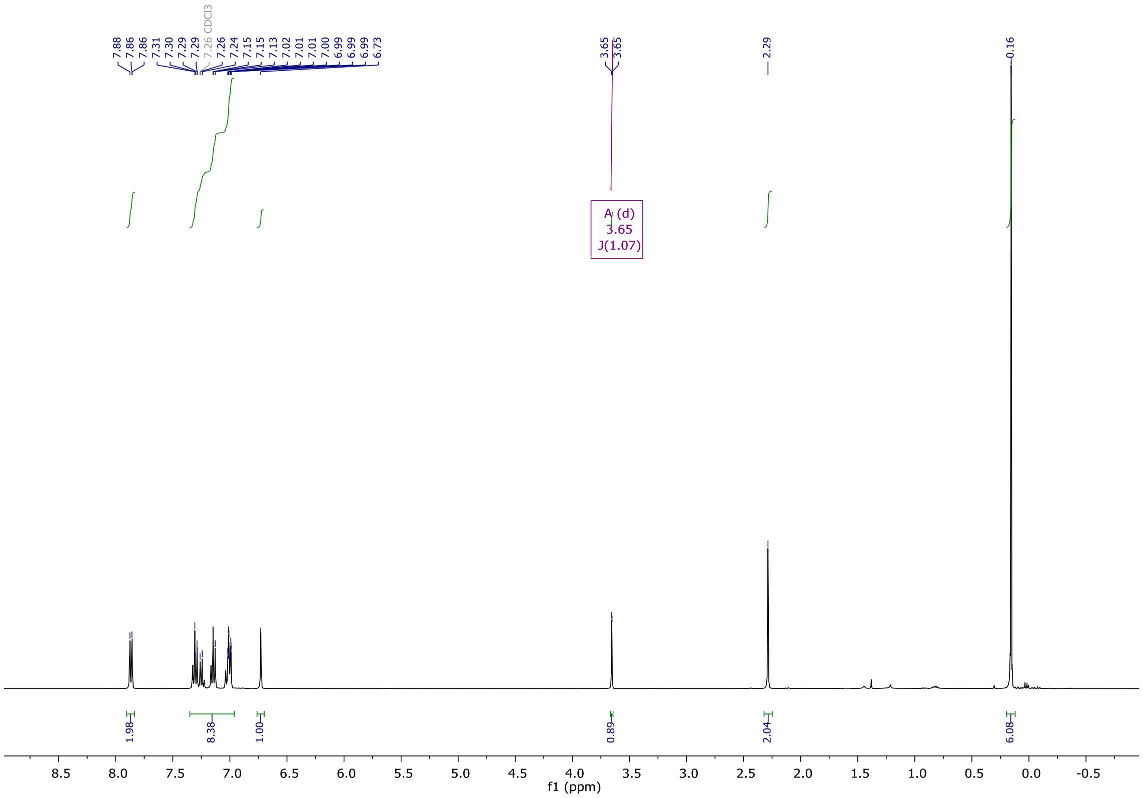

Figure S54. ^{1}H NMR spectrum of **11**.

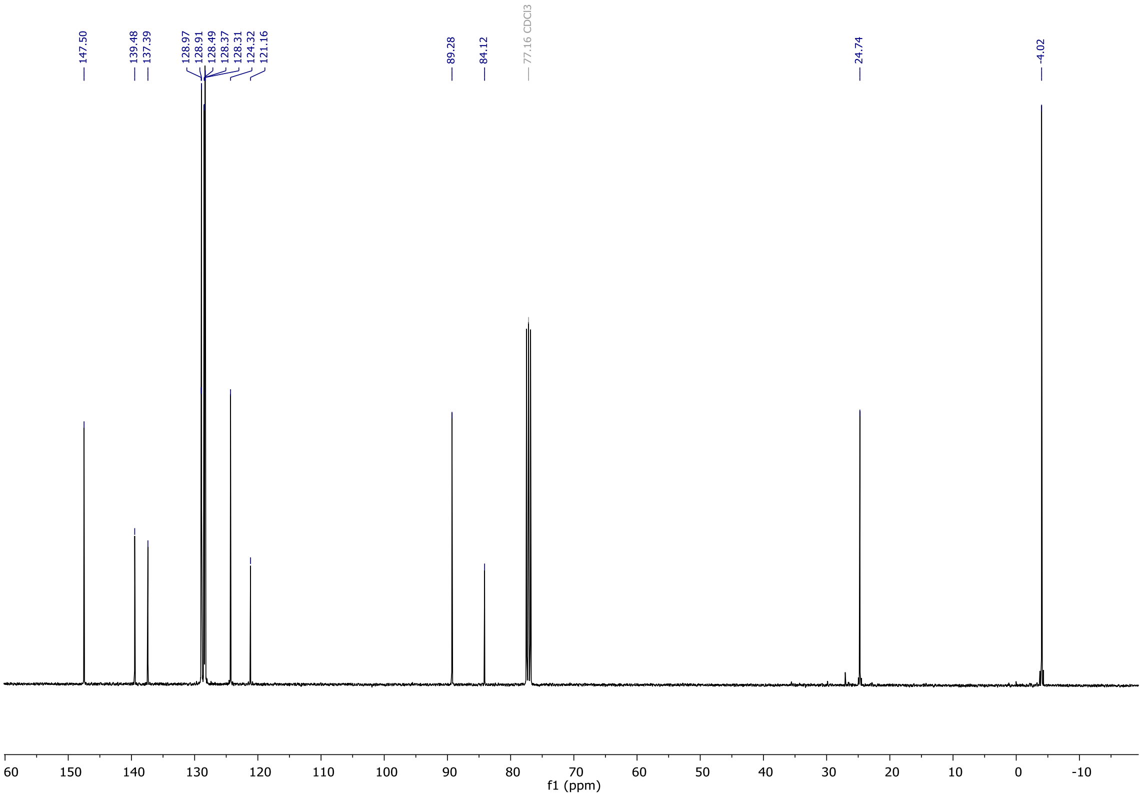

Figure S55. ^{13}C NMR spectrum of **11**.

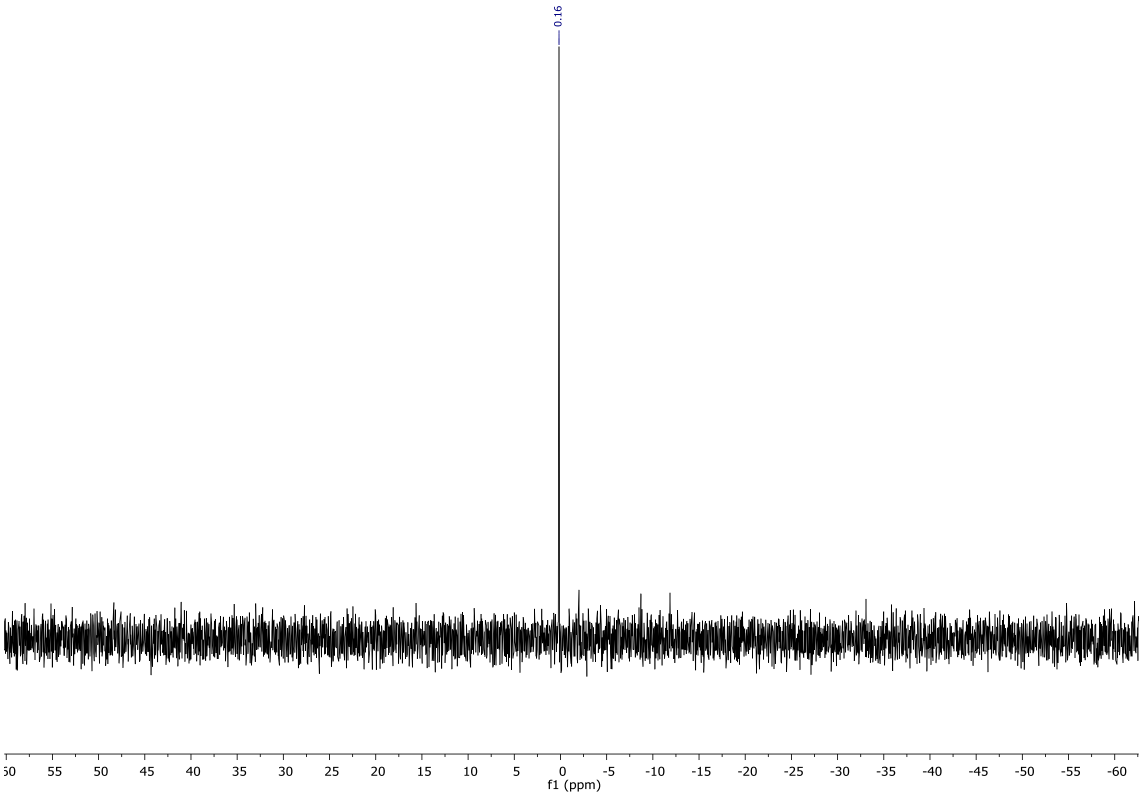

Figure S56. ^{29}Si NMR spectrum of **11**.

(*E*)-Benzyldimethyl(1-phenyl-4-(p-tolyl)but-1-en-3-yn-2-yl)silane (12) – New compound

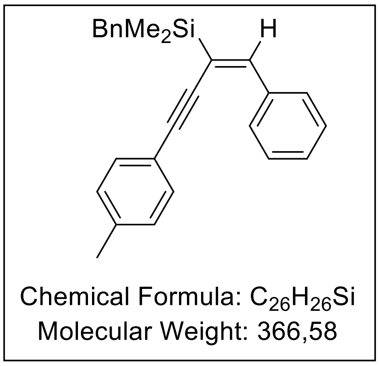

^{1}H NMR ($CDCl_3$, 400 MHz, δ, ppm): 8.07-7.12 (m, 14H, Ph), 6.83 (s, 1H, =CH), 2.47 (s, 2H, CH_2Ph), 2.42 (s, 3H, CH_3), 0.34 (s, 6H, SiCH_3). **^{13}C NMR** ($CDCl_3$, 101 MHz, δ, ppm): 144.8 (=CH), 139.6 (C=CH), 138.4, 137.8, 131.4, 129.3, 128.9, 128.6, 128.5, 128.4, 128.3, 124.3, 122.0, 121.3 (Ph), 101.7 (C≡CPh), 89.8 (C≡CPh), 25.1 (CH_2Ph), 21.6 (PhCH_3), -3.8 (SiCH_3). **^{29}Si NMR** ($CDCl_3$, 79 MHz, δ, ppm): -0.09 (SiMe$_2$Bn). **GC-MS** (EI, 70 eV) m/z (rel. int., %): 366.4 (M^+, 25.0), 365.6 (22.0), 275.4 (26.8), 260.6 (36.2), 259.6 (53.9), 259.1 (100.0), 174.7 (21.0), 173.7 (85.5), 172.8 (84.1), 149.6 (17.5), 144.9 (18.4), 121.6 (31.3), 120.8 (23.9), 91.6 (25.3), 59.6 (36.8), 58.8 (22.7). **FT-IR** (cm^{-1}): 3058, 3023, 2957, 2894, 1599, 1557, 1508, 1492, 1451, 1406, 1247, 1205, 1154, 1056, 1025, 906, 827, 814, 754, 732, 689, 630, 593, 525, 473. **Elem. Anal.** calcd for $C_{26}H_{26}Si$: C, 85.19; H, 7.15; found C, 85.13; H, 7.12. Pale yellow oil. Isolated yield = 74% (68.7 mg).

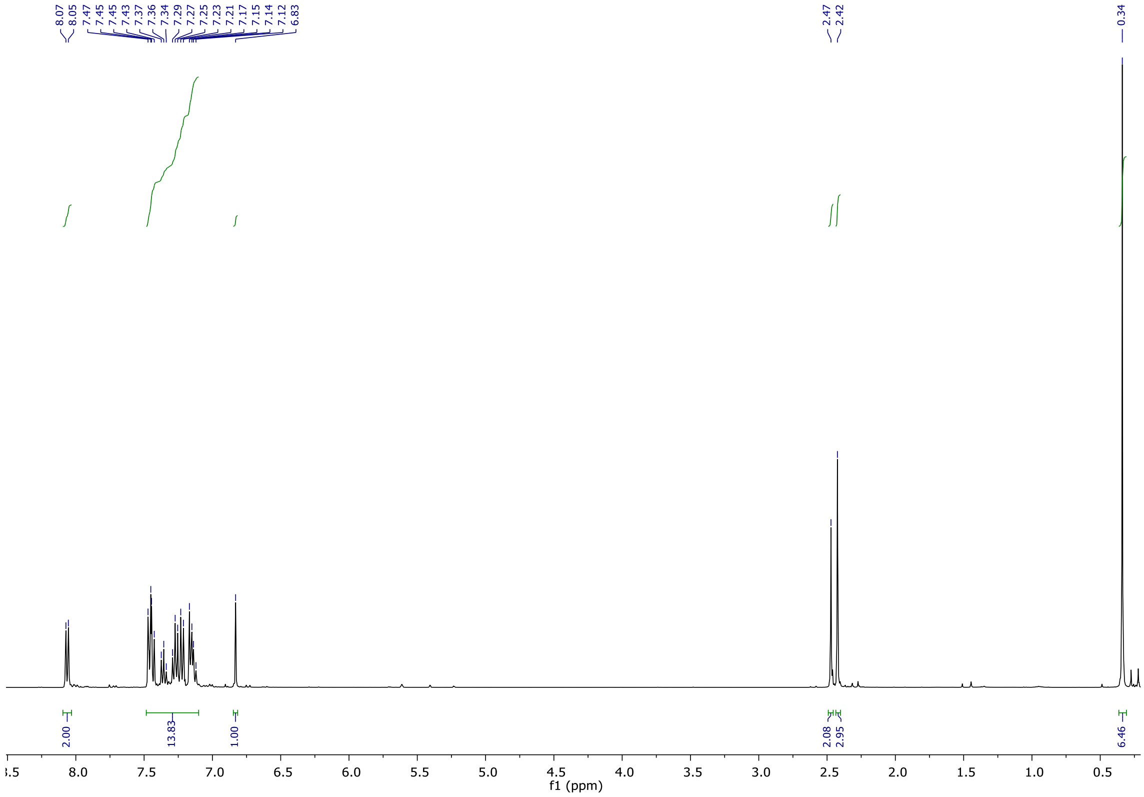

Figure S57. ^{1}H NMR spectrum of **12**.

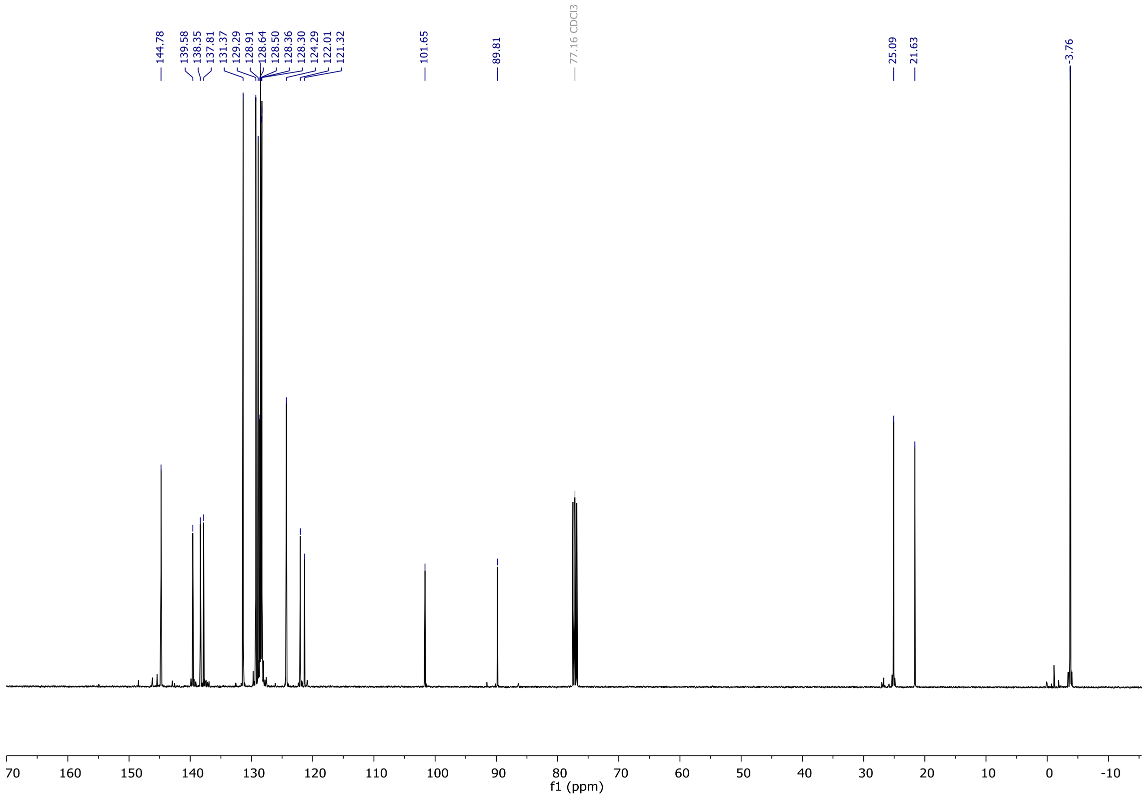

Figure S58. ^{13}C NMR spectrum of **12**.

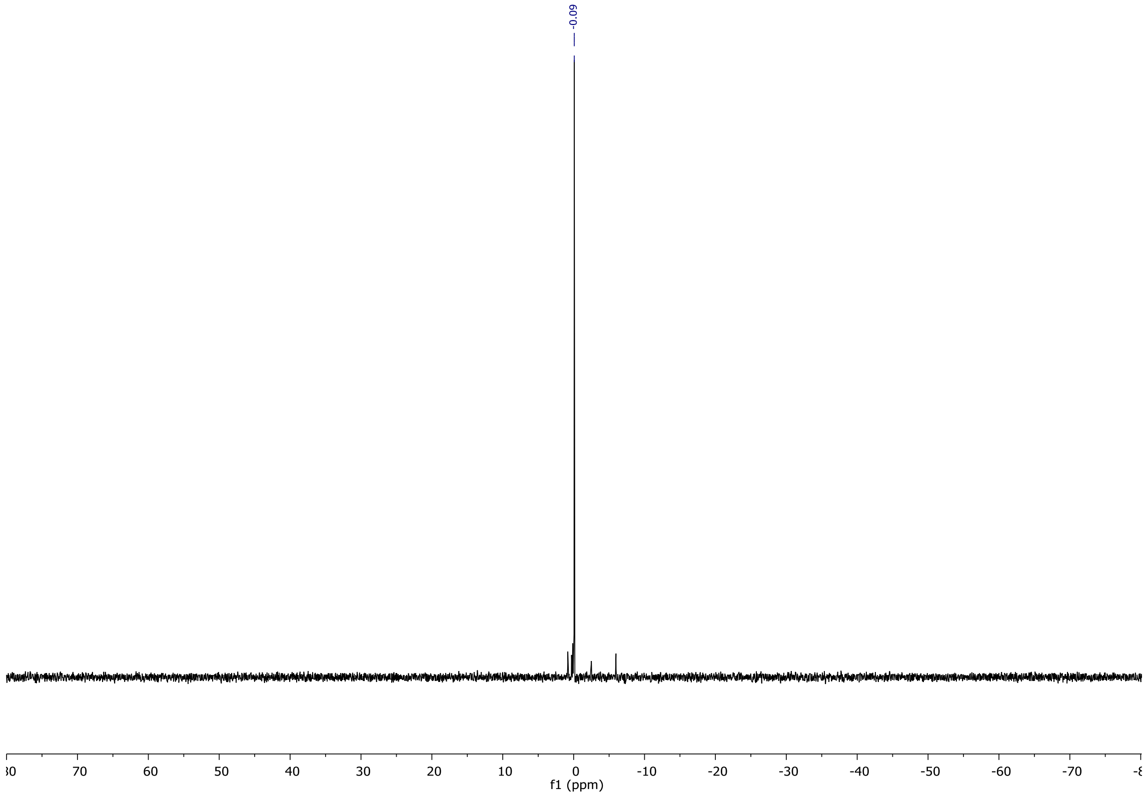

Figure S59. ^{29}Si NMR spectrum of **12**.

3-((1*E*,3*E*)-3-(benzyldimethylsilyl)-4-phenyl-1-(p-tolyl)buta-1,3-dien-2-yl)-1,1,1,5,5,5-hexamethyl-3-((trimethylsilyl)oxy)trisiloxane (13) – New compound

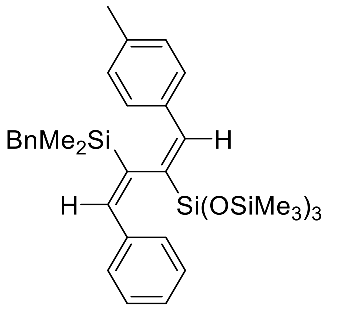

Chemical Formula: $C_{35}H_{54}O_3Si_5$
Molecular Weight: 663,24

^{1}H NMR ($CDCl_3$, 400 MHz, δ, ppm): 7.62-6.94 (m, 14H, Ph), 6.90 (s, 1H, =CH), 6.69 (s, 1H, =CH), 2.33 (s, 3H, CH_3), 2.17 (s, 2H, CH_2Ph), 0.08 (s, 27H, $SiCH_3$), -0.03 (s, 3H, CH_3), -0.06 (s, 3H, CH_3). **^{13}C NMR** ($CDCl_3$, 101 MHz, δ, ppm): 145.1, 141.9, 140.5, 139.4, 137.5, 137.4, 136.8, 136.7, 129.2, 129.0, 128.9, 128.6, 128.3, 128.1, 127.1, 124.0 (Ph, =CH, C=CH), 26.7 (CH_2Ph), 21.4 ($PhCH_3$), 2.0 ($OSiCH_3$), -2.4, -2.7 ($SiCH_3$). **^{29}Si NMR** ($CDCl_3$, 191 MHz, δ, ppm): 8.09 ($Si(OSiMe_3)_3$), -2.82 ($SiMe_2Bn$), -81.99 ($Si(OSiMe_3)_3$). **FT-IR** (cm^{-1}): 2957, 1600, 1492, 1451, 1250, 1205, 1043, 835, 790, 754, 696, 583. **Elem. Anal.** calcd for $C_{35}H_{54}O_3Si_5$: C, 63.38; H, 8.21; found C, 63.43; H, 8.24. Pale yellow oil. Isolated yield = 84% (60.8 mg).

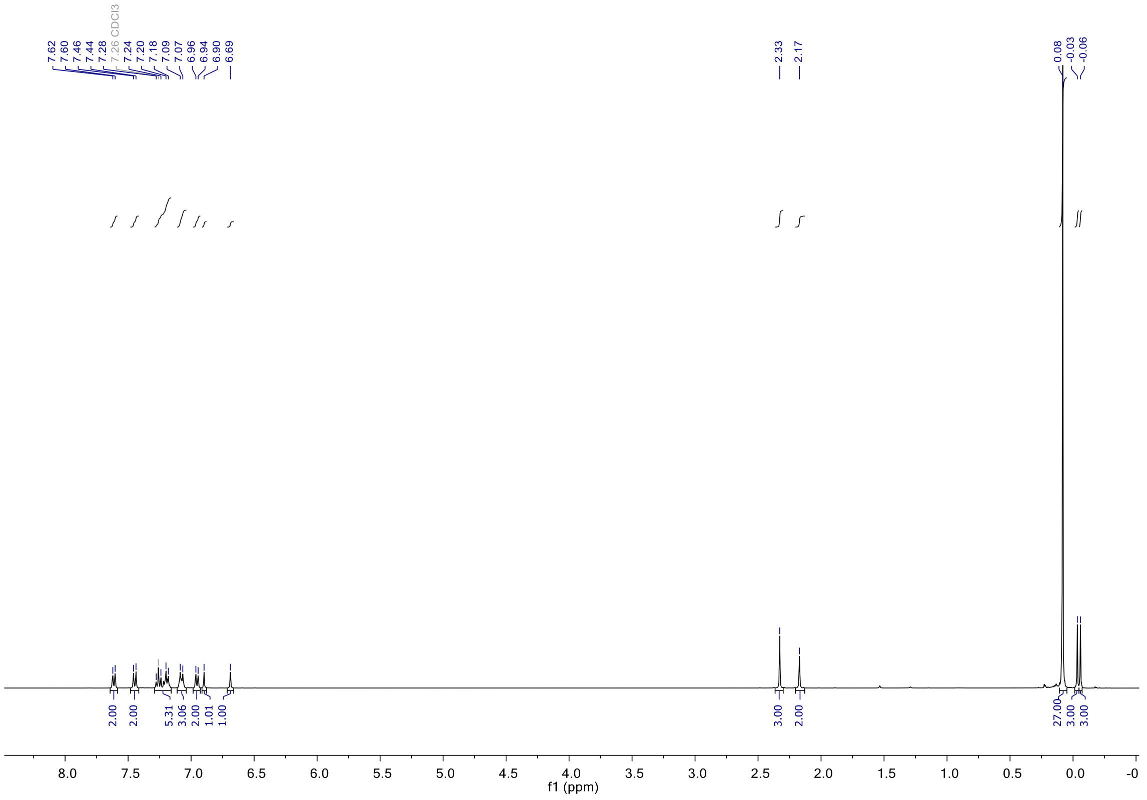

Figure S60. ^{1}H NMR spectrum of **13**.

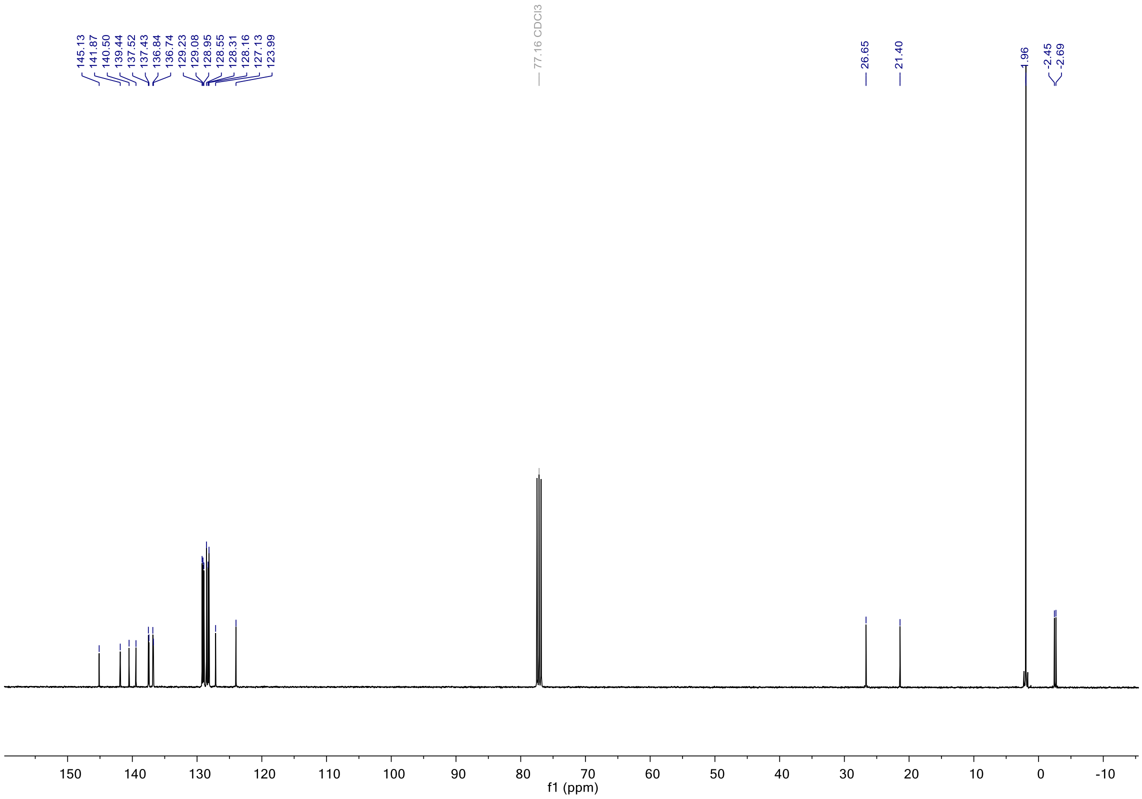

Figure S61. ^{13}C NMR spectrum of **13**.

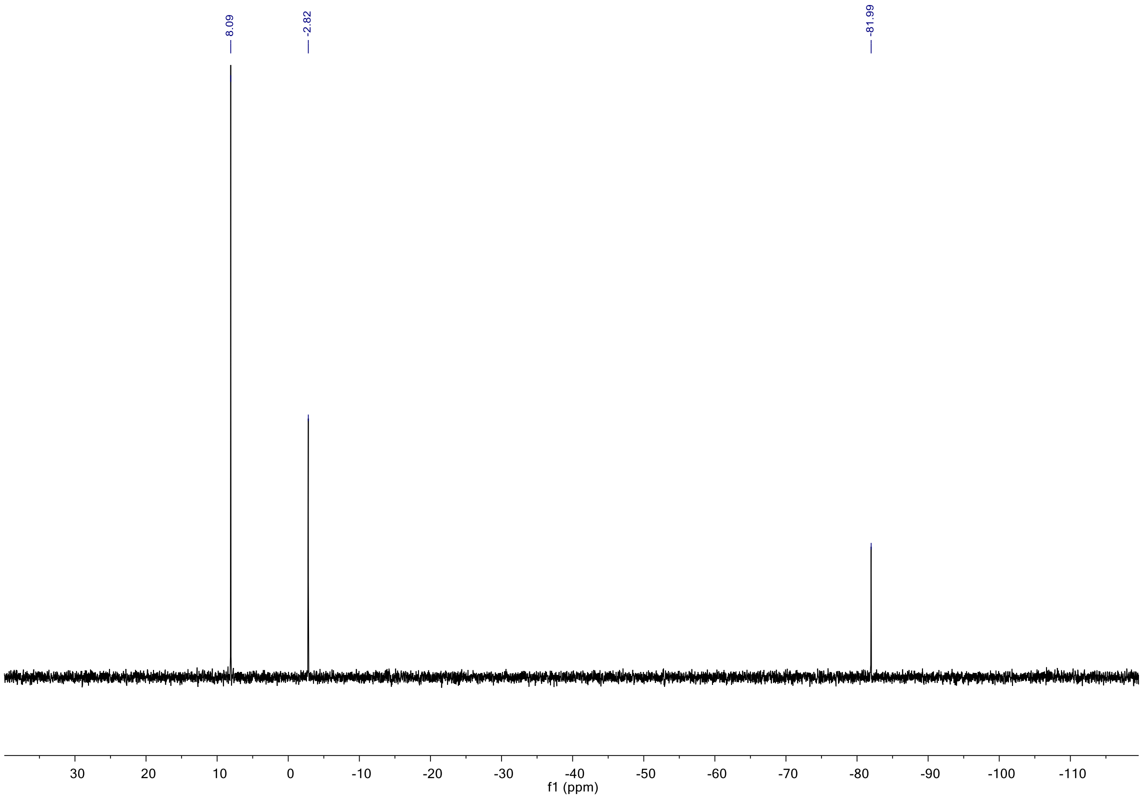

Figure S62. ^{29}Si NMR spectrum of **13**.

[1] S. Rogalski, N. Szymaszek, C. Pietraszuk, Organics, 4 (2023) 206.
[2] J. Szyling, A. Szymańska, A. Franczyk, J. Walkowiak, The Journal of Organic Chemistry, 87 (2022) 10651.
[3] K. Stefanowska-Kątna, M. Szymkowiak, J. Nagórny, A. Czapik, J. Walkowiak, A. Franczyk, Journal of Catalysis, 434 (2024) 115519.
[4] T. Sokolnicki, K. Stefanowska-Kątna, A. Czapik, J. Walkowiak, A. Franczyk, International Journal of Molecular Sciences, 25 (2024) 12208.
[5] T. Kusumoto, K. Ando, T. Hiyama, Bulletin of the Chemical Society of Japan, 65 (1992) 1280.
[6] T. Kusumoto, T. Hiyama, Chemistry Letters, 14 (1985) 1405.
[7] R.D. Adams, U. Bunz, B. Captain, W. Fu, W. Steffen, Journal of Organometallic Chemistry, 614-615 (2000) 75.
[8] A. Tillack, S. Pulst, W. Baumann, H. Baudisch, K. Kortus, U. Rosenthal, Journal of Organometallic Chemistry, 532 (1997) 117.
